# *Fam83h* null mice support a neomorphic mechanism for human ADHCAI

**DOI:** 10.1002/mgg3.178

**Published:** 2015-09-21

**Authors:** Shih‐Kai Wang, Yuanyuan Hu, Jie Yang, Charles E. Smith, Amelia S Richardson, Yasuo Yamakoshi, Yuan‐Ling Lee, Figen Seymen, Mine Koruyucu, Koray Gencay, Moses Lee, Murim Choi, Jung‐Wook Kim, Jan C‐C. Hu, James P. Simmer

**Affiliations:** ^1^ Department of Biologic and Materials SciencesUniversity of Michigan School of Dentistry1210 Eisenhower Pl.Ann ArborMichigan48108; ^2^ Department of Pediatric DentistrySchool and Hospital of StomatologyPeking University22 South Avenue ZhongguancunHaidian DistrictBeijing100081China; ^3^ Facility for Electron Microscopy ResearchDepartment of Anatomy and Cell Biology and Faculty of DentistryMcGill University3640 University StreetMontrealQuebecH3A 2C7Canada; ^4^ Department of Biochemistry and Molecular BiologySchool of Dental MedicineTsurumi University2‐1‐3 TsurumiTsurumi‐kuYokohama230‐8501Japan; ^5^ Graduate Institute of Clinical DentistryNational Taiwan UniversityNo. 1, Chang‐Te StTaipei10048Taiwan; ^6^ Department of PedodonticsFaculty of DentistryIstanbul UniversityIstanbulTurkey; ^7^ Department of Biomedical SciencesSeoul National University College of Medicine275‐1 Yongon‐dongChongno‐guSeoul110‐768Korea; ^8^ Department of Pediatric Dentistry & Dental Research InstituteSchool of DentistrySeoul National University275‐1 Yongon‐dongChongno‐guSeoul110‐768Korea

**Keywords:** Amelogenesis imperfecta, gain‐of‐function, hair defects, knockout mouse, skin defects, truncation mutation

## Abstract

Truncation mutations in FAM83H (family with sequence similarity 83, member H) cause autosomal dominant hypocalcified amelogenesis imperfecta (ADHCAI), but little is known about FAM83H function and the pathogenesis of ADHCAI. We recruited three ADHCAI families and identified two novel (p.Gln457*; p.Lys639*) and one previously documented (p.Q452*) disease‐causing *FAM83H* mutations. We generated and characterized *Fam83h*‐knockout/*lac*Z‐knockin mice. Surprisingly, enamel thickness, density, Knoop hardness, morphology, and prism patterns were similar in *Fam83h* ^+/+^, *Fam83h* ^+/−^, and *Fam83h* ^−/−^ mice. The histology of ameloblasts in all stages of development, in both molars and incisors, was virtually identical in all three genotypes and showed no signs of pathology, although the *Fam83h* ^−/−^ mice usually died after 2 weeks and rarely survived to 7 weeks. *LacZ* expression in the knockin mice was used to report *Fam83h* expression in the epithelial tissues of many organs, notably in skin and hair follicles, which manifested a disease phenotype. Pull‐down studies determined that FAM83H dimerizes through its N‐terminal phospholipase D‐like (PLD‐like) domain and identified potential FAM83H interacting proteins. Casein kinase 1 (CK1) interacts with the FAM83H PLD‐like domain via an F^270^‐X‐X‐X‐F^274^‐X‐X‐X‐F^278^ motif. CK1 can phosphorylate FAM83H in vitro*,* and many phosphorylation sites were identified in the FAM83H C‐terminus. Truncation of FAM83H alters its subcellular localization and that of CK1. Our results support the conclusion that FAM83H is not necessary for proper dental enamel formation in mice, but may act as a scaffold protein that localizes CK1. ADHCAI is likely caused by gain‐of‐function effects mediated by truncated FAM83H, which potentially mislocalizes CK1 as part of its pathological mechanism.

## Introduction

Amelogenesis imperfecta (AI) is a group of human inherited disorders characterized by enamel malformations with or without nondental phenotypes (Witkop and Sauk 1976). Hypocalcified AI is a special form of AI in which the malformed enamel is of normal thickness but cheesy‐soft, and may be lost soon after tooth eruption. The genetic etiology of human autosomal dominant hypocalcified AI (ADHCAI, OMIM *130900) had been long sought, but remained unknown until mutations in a previously uncharacterized gene, *FAM83H* (family with sequence similarity 83, member H; OMIM *611927), were first identified through a genome‐wide search (Mendoza et al. 2007; Kim et al. 2008). Subsequently, many disease‐causing *FAM83H* mutations were reported by different groups (Kim et al. 2008; Lee et al. 2008, 2011; Ding et al. 2009; Hart et al. 2009; Hyun et al. 2009; Wright et al. 2009, 2011; El‐Sayed et al. 2010; Chan et al. 2011; Haubek et al. 2011; Song et al. 2012; Wang et al. 2013), and *FAM83H*‐associated AI appeared to be the most prevalent form of AI in North America (Chan et al. 2011).

Twenty different disease‐causing *FAM83H* mutations had so far been identified, with some reported more than once (Fig. S1). Noticeably, all the reported mutations were either nonsense mutations or frameshifts leading to a premature stop codon. No other types of loss‐of‐function mutations, such as missense mutations, have been reported to be cause of the disease. More interestingly, all of the *FAM83H* mutations localized within a specific 5′ region of the last exon (Exon 5) of FAM83H generated a mutant transcript that would escape nonsense mediated decay (Shyu et al. 2008) and translate a truncated protein ending between amino acids 287 and 694, whereas the wild‐type protein has 1179 amino acids. This mutational homogeneity highly suggests a dominant negative effect or a gain of function in the pathogenesis of ADHCAI. In contrast, haploinsufficiency is a less plausible pathological mechanism, as other types of loss‐of‐function mutations have not been found to cause a disease phenotype.

Unlike the enamel matrix proteins and proteases important for enamel formation, FAM83H does not have a signal peptide and is an intracellular protein shown to be associated with the transGolgi network (Ding et al. 2009). The primary structure of FAM83H gives little indication of its potential function. Based upon bioinformatic structure and domain predictions, FAM83H has neither well‐defined structural characteristics nor known functional domains except an N‐terminal phospholipase D‐like (PLD‐like) domain (cd09188), which is the shared element among all members in FAM83 family and gives the group its identity. However, the homology between this domain and PLD is trace and probably only indicative of a conserved secondary structure and similar three‐dimensional fold. It is unlikely that FAM83H has PLD‐like enzymatic activity.

So far, mutational studies are the only evidence indicating the physiological significance of FAM83H in humans. However, the actual functions of this protein inside the cell and the pathogenesis of its associated enamel defects are largely unknown. In this study, we recruited three families with ADHCAI and identified two novel and one previously reported *FAM83H* mutations causing enamel defects. In order to investigate the roles of FAM83H in enamel formation, we generated *Fam83h*‐knockout/*lac*Z‐knockin mice. By analyzing the enamel phenotypes of the *Fam83h* ^*−/−*^ and *Fam83h* ^*+/−*^ mice in vivo and molecularly characterizing FAM83H protein in vitro, we identified potential intracellular functions of FAM83H and proposed a plausible pathological mechanism for human ADHCAI.

## Results

### Novel *FAM83H* mutations causing ADHCAI

**Figure 1:**
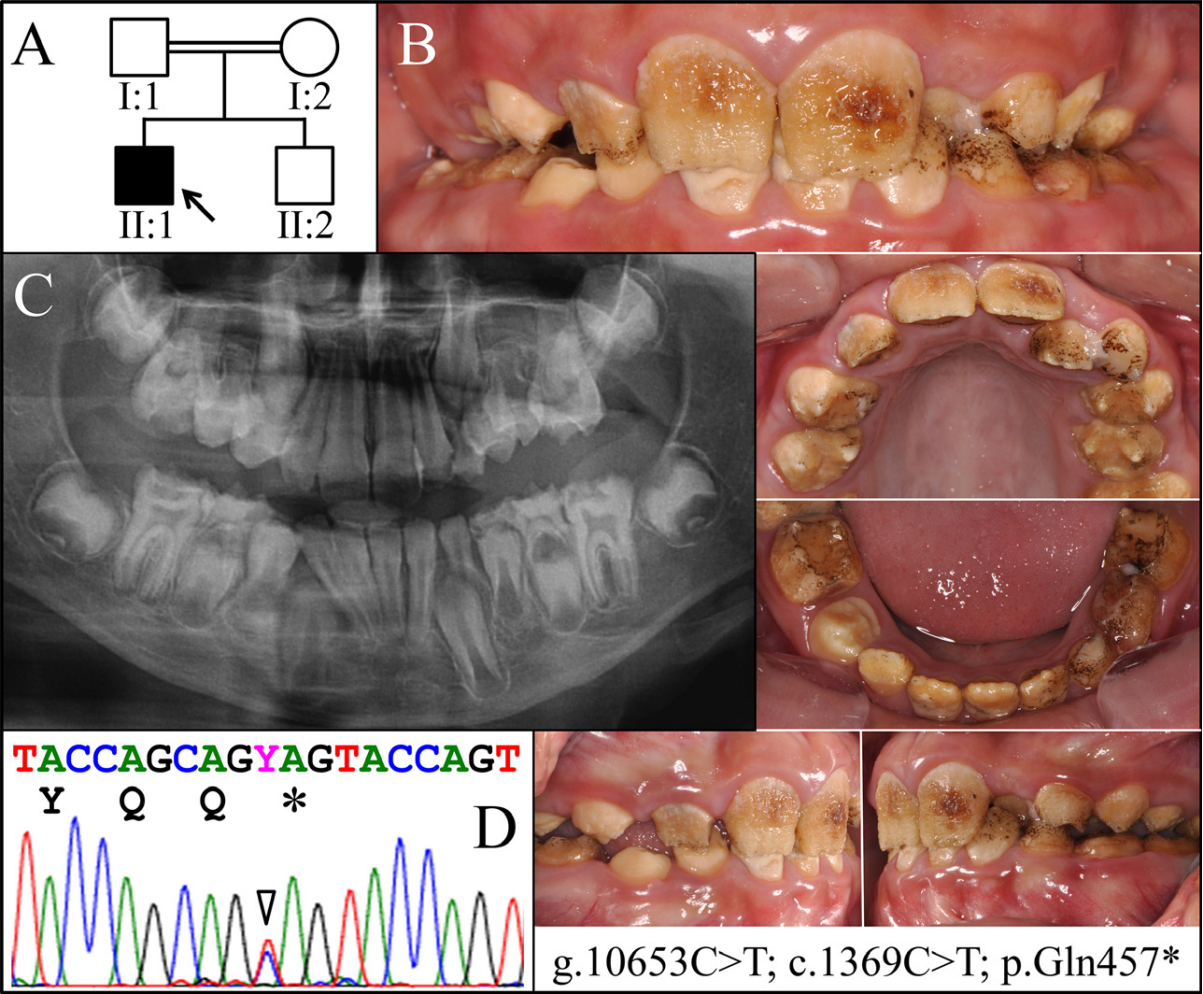
Family 1. A: Pedigree of Family 1, Proband (II:1). B: Oral photographs showing dental enamel malformations catergorized as autosomal dominant hypocalcified amelogenesis imperfecta (ADHCAI). C: Panorex radiograph showing hypocalcified enamel that contrasts poorly with dentin. D: Chromatogram showing that the proband was heterozygous for the novel *FAM83H* mutation (g.10653C>T; c.1369C>T) that converted a glutamine codon (CAG) at postion 457 into a premature termination codon (TAG). Y  =  C or T.

We recruited three families with dental enamel defects resembling hypocalcified amelogenesis imperfecta (Kim et al. 2008) and conducted mutational analyses. The proband of Family 1 (II:1) was a 10‐year‐old boy of Turkish descent. With parental consanguinity, he was the only affected individual in the family, suggesting that the enamel defect was caused by a recessive or a de novo mutation (Fig. 1A). Clinically, the enamel of both his primary and permanent teeth (mixed dentition) was yellow–brown discolored and cheesy‐soft (Fig. 1B). While the defective enamel chipped off from most tooth surfaces, some enamel was noted over cusp tips and cervical areas of several teeth. All erupted teeth showed extensive posteruptive attrition except for the lower right first bicuspid, which had just emerged into the oral cavity. Radiographically, the proband presented with a full set of permanent teeth excluding third molars (Fig. 1C). The enamel of unerupted teeth appeared to be of normal thickness but did not contrast with underlying dentin. Whole exome analysis of proband's DNA identified no potential disease‐causing mutations in known AI candidate genes except a novel nonsense mutation in a single allele of *FAM83H* (g.10653C>T, c.1369C>T, and p.Gln457*). The proband's brother (Fig. S2), father (Fig. S3), and mother (Fig. S4) were all unaffected and did not carry the mutation. The genetic analysis confirmed the diagnosis of hypocalcified AI and demonstrated that the enamel defects in the proband were caused by a spontaneous de novo mutation in *FAM83H* and should subsequently show an autosomal dominant pattern of inheritance.

**Figure 2:**
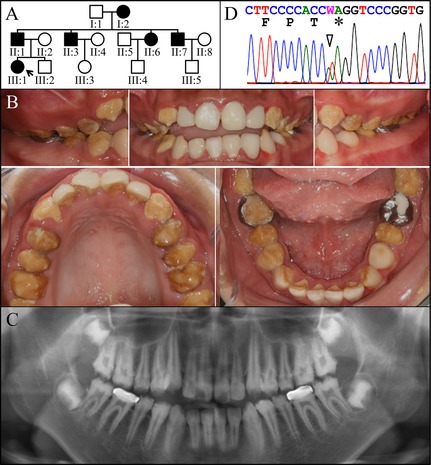
Family 2. A: Pedigree of Family 2 Proband (III:1). B: Oral photographs showing dental enamel malformations catergorized as autosomal dominant hypocalcified amelogenesis imperfecta (ADHCAI). C: Panorex radiograph showing hypocalcified enamel that contrasts poorly with dentin. D: Chromatogram showing that the proband was heterozygous for *FAM83H* mutation (g.11199A>T; c.1915A>T) that converted a Lysine codon (AAG) at position 639 into a premature termination codon (TAG). W = A or T.

Family 2 was a Taiwanese family in which the proband (III:1) was a 12‐year‐old at the time of examination (Fig. 2). According to the mother's (II:2) report, the enamel defects were inherited from the paternal grandmother (I:2). The proband's father (II:1), aunt (II:6), and two uncles (II:3, II:7) were all affected, indicating that the enamel malformations were caused by a dominant mutation (Fig. 2A). The proband had a complete permanent dentition (32 teeth) and an anterior open bite. The tooth crowns were yellow–brown discolored, and the enamel had chipped off from most tooth surfaces (Fig. 2B). The incisors had been restored with laminate veneers, but their lingual surfaces were exposed and showed defective enamel. The proband's primary teeth were reported to be affected. The panoramic radiograph (Fig. 2C) showed attrition on most of the erupted teeth, while the unerupted third molars showed enamel of normal thickness that did not contrast with dentin. A diagnosis of ADHCAI was made based upon the dominant pattern of inheritance and enamel phenotype, which implicated *FAM83H* as a likely candidate in its etiology. Target gene mutational analysis revealed a novel *FAM83H* nonsense mutation (g.11199A>T, c.1915A>T, and p.Lys639*) that segregated with the disease phenotype (Fig. S5).

**Figure 3:**
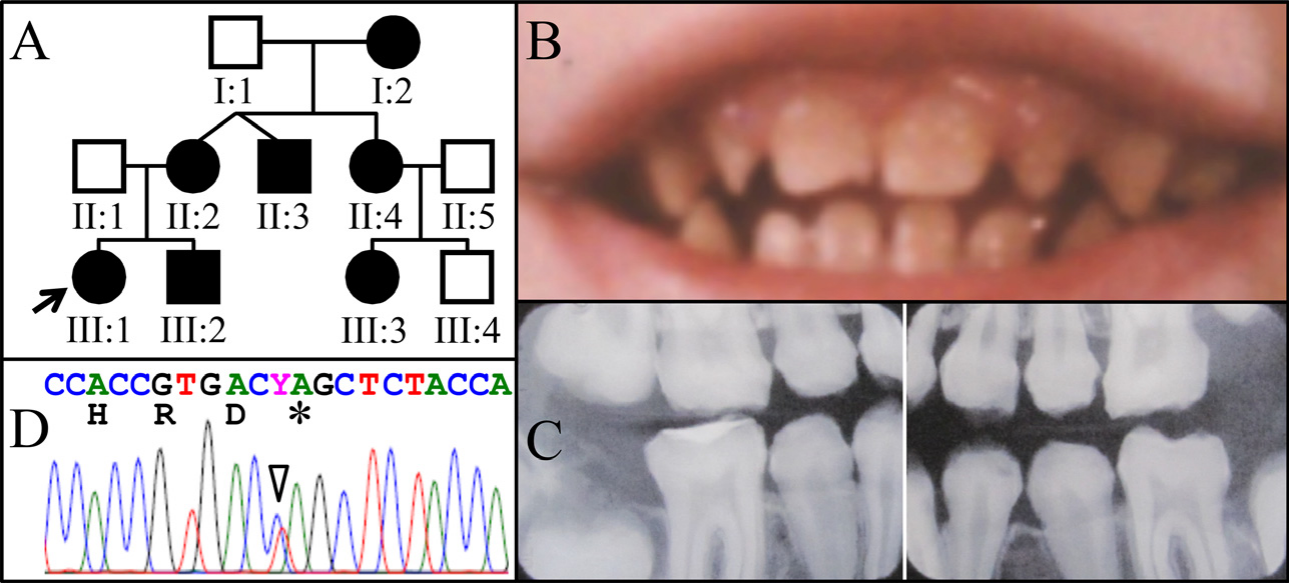
Family 3. A: Pedigree of Family 3 Proband (III:1). B: Oral photograph showing dental enamel malformations in primary dentition. C: Bitewing radiographs showing hypocalcified enamel of permanent teeth at age 12 that have already undergone extensive attrition. D: Chromatogram showing that the proband was heterozygous for *FAM83H* mutation (g.10638C>T and c.1354C>T) that converted a glutamine codon (CAG) at codon 452 into a premature termination codon (TAG). Y  =  C or T.

The proband of Family 3 (III:1) was a female of Jewish descent. The enamel malformation reportedly ran in every generation of the maternal family for at least six generations, suggesting a dominant pattern of disease inheritance (Fig. 3A). Although the mother (II:2) and the younger brother (III:2) were also affected, we were only able to collect dental records and a DNA sample from the proband. The proband's permanent teeth had been fully reconstructed, but she provided a family photo showing her front teeth prior to dental reconstruction (Fig. 3B). The enamel of the primary and permanent teeth was defective. Bitewing radiographs taken at an age of 12 years showed the enamel of her permanent teeth had similar defects to those of the probands from the first two families (Fig. 3C). Therefore, we directly screened the *FAM83H* coding region and identified a previously reported disease‐causing mutation (g.10638C>T, c.1354C>T, and p.Q452*), which confirmed the diagnosis of ADHCAI.

The three *FAM83H* mutations we identified were all nonsense mutations within the critical region of exon 5. All of the (now 22) reported *FAM83H* disease‐causing truncation mutations are localized in this region (Fig. S1), which strongly supports the interpretation that *FAM83H* mutations were responsible for the observed enamel defects in our three families.

### Generation of *Fam83h*‐knockout/*lac*Z‐knockin mice and dental phenotype

To better understand the functions of FAM83H in dental enamel formation, we ablated *Fam83h* in mice of C57BL/6 strain by introducing a *lac*Z reporter gene encoding *β*‐galactosidase fused to a mouse NLS to replace the entire *Fam83h* coding and 3′ untranslated (UTR) regions (Fig. S6A), which enabled a simple PCR genotyping strategy (Fig. S6B). The 5′ part of the wild‐type *Fam83h* gene up to the translation initiation codon (ATG) in exon 2 is unaltered. Then, the entire coding region of *Fam83h* (Fig. S7A) is replaced by the coding region for ß‐galactosidase with a nuclear localization signal (NLS‐*lac*Z) and 3′ UTR containing two polyadenylation signals (Fig. S7B). Retention of the 5′ transcriptional regulatory region increases the confidence that the NLS‐*lac*Z reporter accurately mimics *Fam83h* transcription in vivo, while deletion of the entire *Fam83h* coding region ensures that no part of FAM83H can be expressed in the null mouse. The deletion of *Fam83h* continues through the last nucleotide of the *Fam83h* 3′ UTR in exon 5 and no further.

Intercrosses of heterozygous mice (*Fam83h* ^+/−^) produced pups of three genotypes at the expected Mendelian ratio (1 + /+: 2 + /−: 1−/−). At birth, homozygous mutants (*Fam83h* ^−/−^) were indistinguishable from their wild‐type (*Fam83h* ^+/+^) and heterozygous (*Fam83h* ^+/−^) littermates. However, most died within 2 weeks after birth, although a few *Fam83h* ^−/−^ mice survived longer. We were only able to get eight null mice aged 7 weeks from more than 50 litters of heterozygous mating. Grossly, while the 7‐week heterozygous mice looked comparable to the wild‐type, the survived null mice appeared smaller in size with a sparse and scruffy coat and exhibited reduced general activity, and could readily be distinguished from wild‐type mice (Fig. S8).

**Figure 4:**
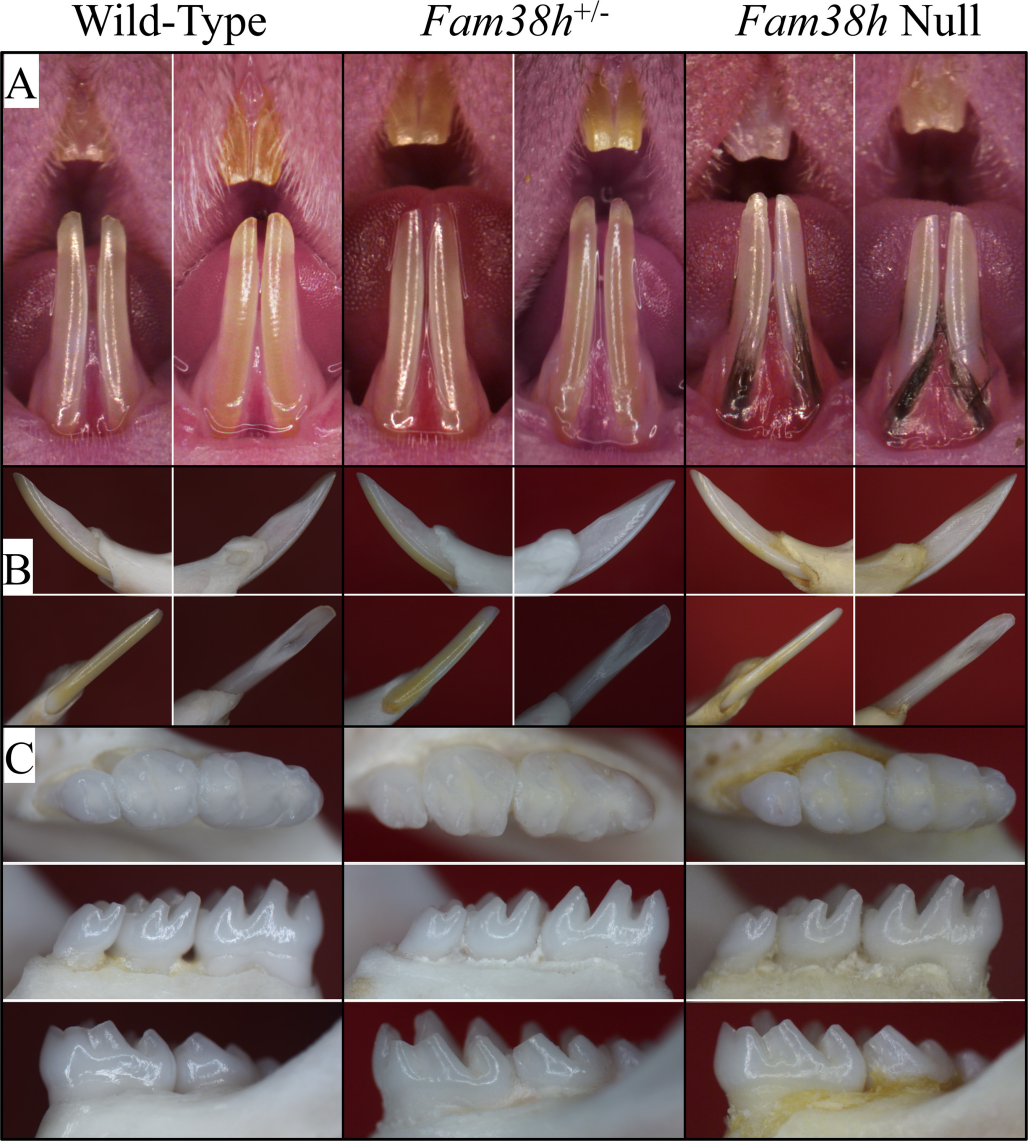
Photos of Mouse Dentitions at 7 weeks. A: Frontal views of incisors. The teeth from all three genotypes look very similar, with no signs of enamel chipping. The null mice have black coat hair stuck in the gingival crevice, which relates to the hair phenotype. B: Going clockwise from upper left: lateral, medial, lingual, labial views of the mandibular incisor. C: Occlusal, lingual, and buccal views of the mandibular incisors. All phenotypes show normal crown morphology, smooth, lusterous enamel without evidence of attrition.

Despite the general weakness of the 7‐week null mice, their incisors appeared normal with glossy and smooth enamel surfaces and sharp incisal tips, similar to those of their *Fam83h* ^+/−^ and *Fam83h* ^+/+^ littermates (Fig. 4A, B). In addition, the basic morphology and enamel of the molars also looked comparable between three genotypes without extensive posteruption attrition in *Fam83h* ^−/−^ mice (Fig. 4C). Noticeably, coat hair was found in the labial gingival sulci of the null mice mandibular incisors, but not in the gingival sulci of heterozygous or wild‐type mice. The gingival sulci of some *Fam83h* ^−/−^ molars were also found to hold hair, which was lost during the removal of soft tissues. The hair was easily removed, suggesting that they were somehow inserted into the place rather than growing out from the sulcus. The inserted hair indicated deep pockets around the *Fam83h* ^−/−^ incisors and molars, although only mild alveolar bone destruction was noted around these teeth.

**Figure 5:**
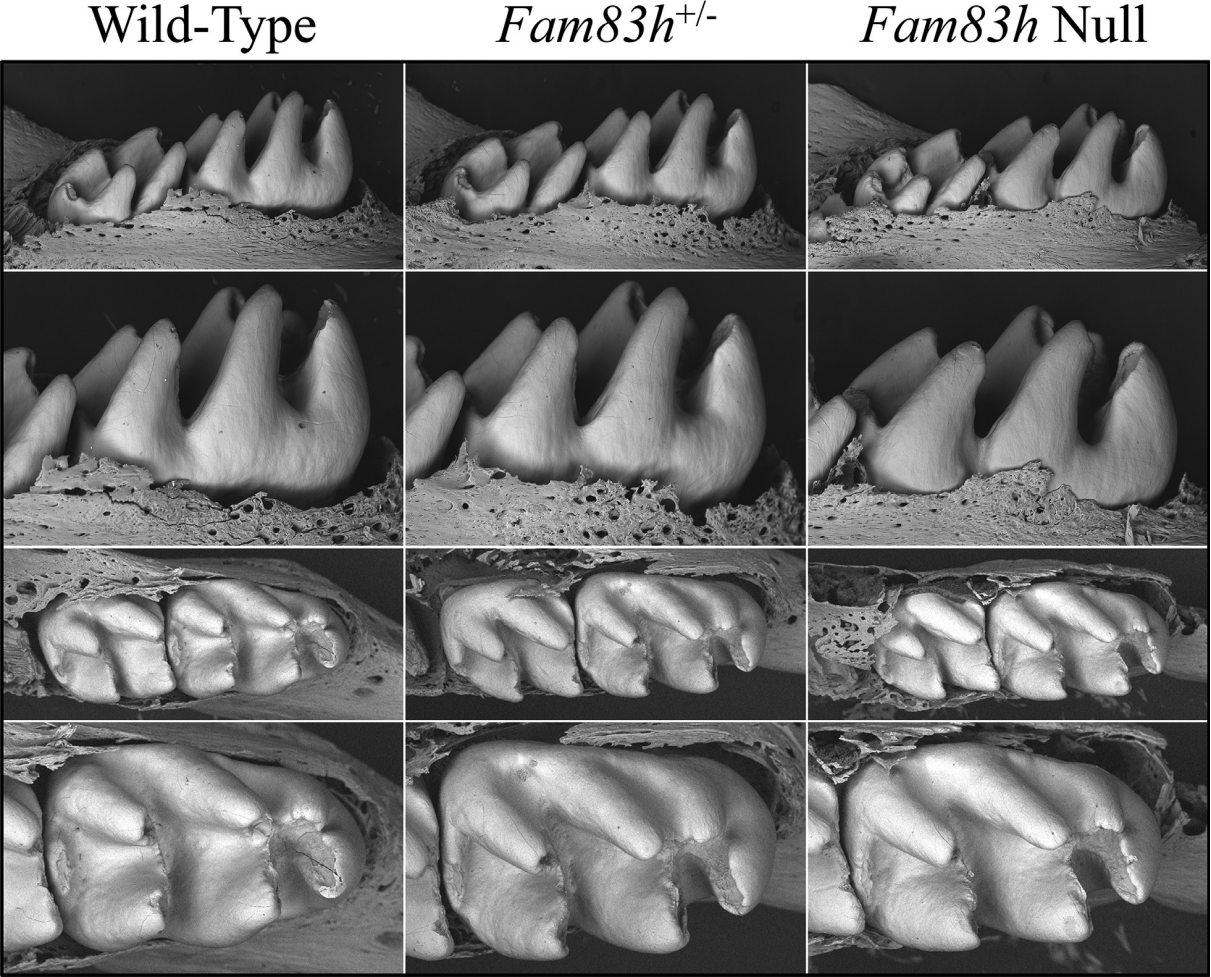
bSEM of D14 Mandibular Molars. D14 is the day before the first molar erupts into function and is often the last day that the developmental form of the molars can still be observed before undergoing potentially rapid attrition following eruption. The crowns from all three genotypes are similar and show no evidence of developmental malformations.

**Figure 6:**
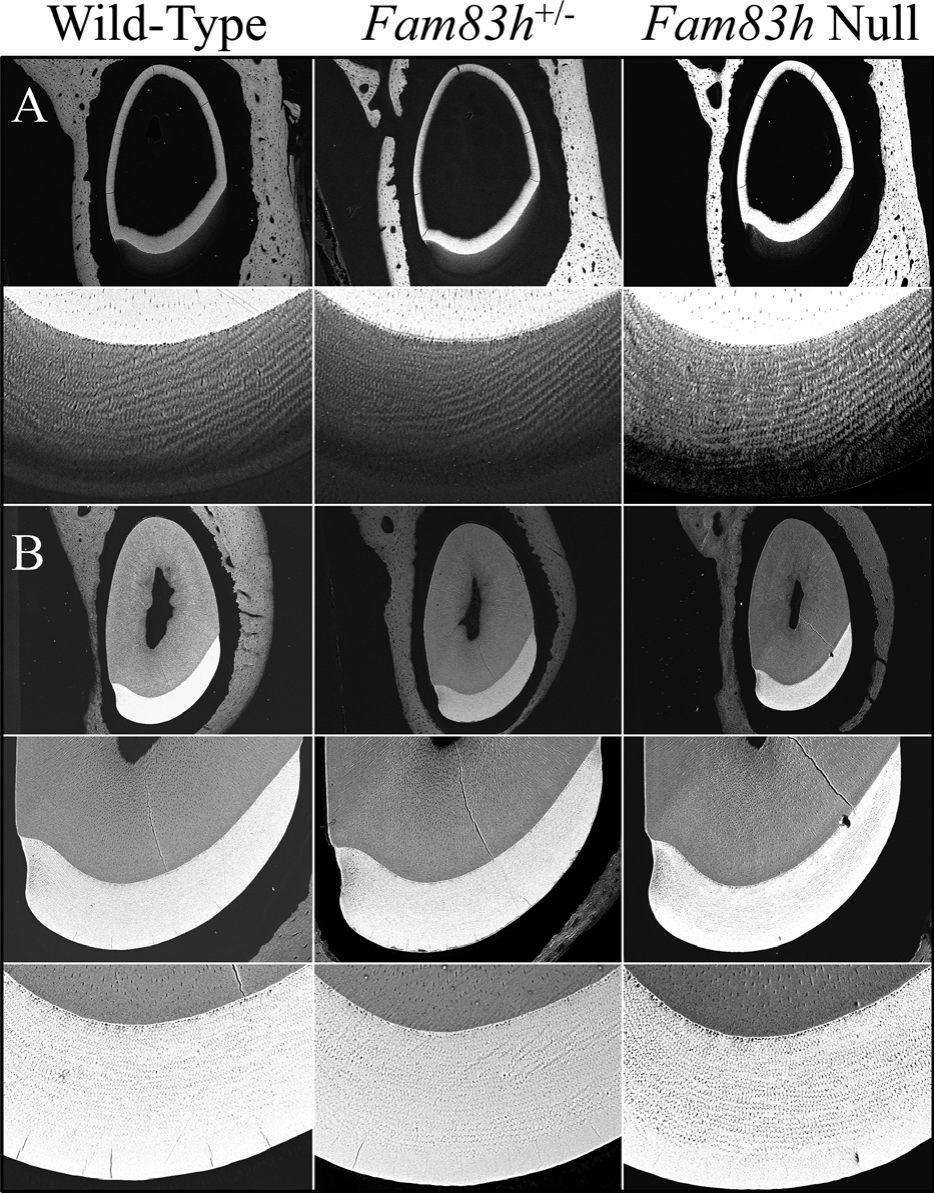
bSEM of 7‐week Mandibular Incisor Cross Sections. A: Sections from level 3, at or nearing the end of the secretory stage. The enamel layer is similar in overall thickness and rod pattern. B: Sections from level 8, at the position of the alveolar crest show that the enamel in all three genotypes has similar thickness, rod pattern, and degree of mineralization.

We examined the developing molars of D14 mice with backscattered scanning electron microscopy (bSEM), when the first molars were about to erupt into the oral cavity (Fig. 5). This is the latest time point that the first molars can be observed before potentially being altered by occlusal forces. Consistent with the findings from incisors, the D14 molars of three genotypes were indistinguishable from one another with normal crown morphology, enamel thickness, and surface texture. The bSEM analyses of sequential cross sections (with 1 mm increments from level 2 to 8) along the continuously growing mandibular incisors also exhibited comparable densities and enamel rod patterns at sequential sections among three genotypes, which indicated a normal progression of mineralization during enamel maturation (Figs. S9–S11). Also, the *Fam83h* ^−/−^ enamel appeared of normal thickness at level 8 (the level of alveolar crest) compared to the *Fam83h* ^+/−^ and *Fam83h* ^+/+^ enamel, suggesting an unimpaired enamel matrix secretion when FAM83H was ablated (Fig. 6). No overt abnormality of dentin and surrounding alveolar bone was noted. However, noticeably, the *Fam83h* ^−/−^ incisor showed a smaller cross‐sectional area of pulp chamber than that of the other two genotypes at comparable levels. This suggests that the incisors erupted more slowly than in the wild‐type mice, which gave the odontoblasts more time to add dentin. Incisors erupt at the rate that they are worn down (Robinson et al. 1988), so a reduction in gnawing behavior might account for this effect.

**Figure 7:**
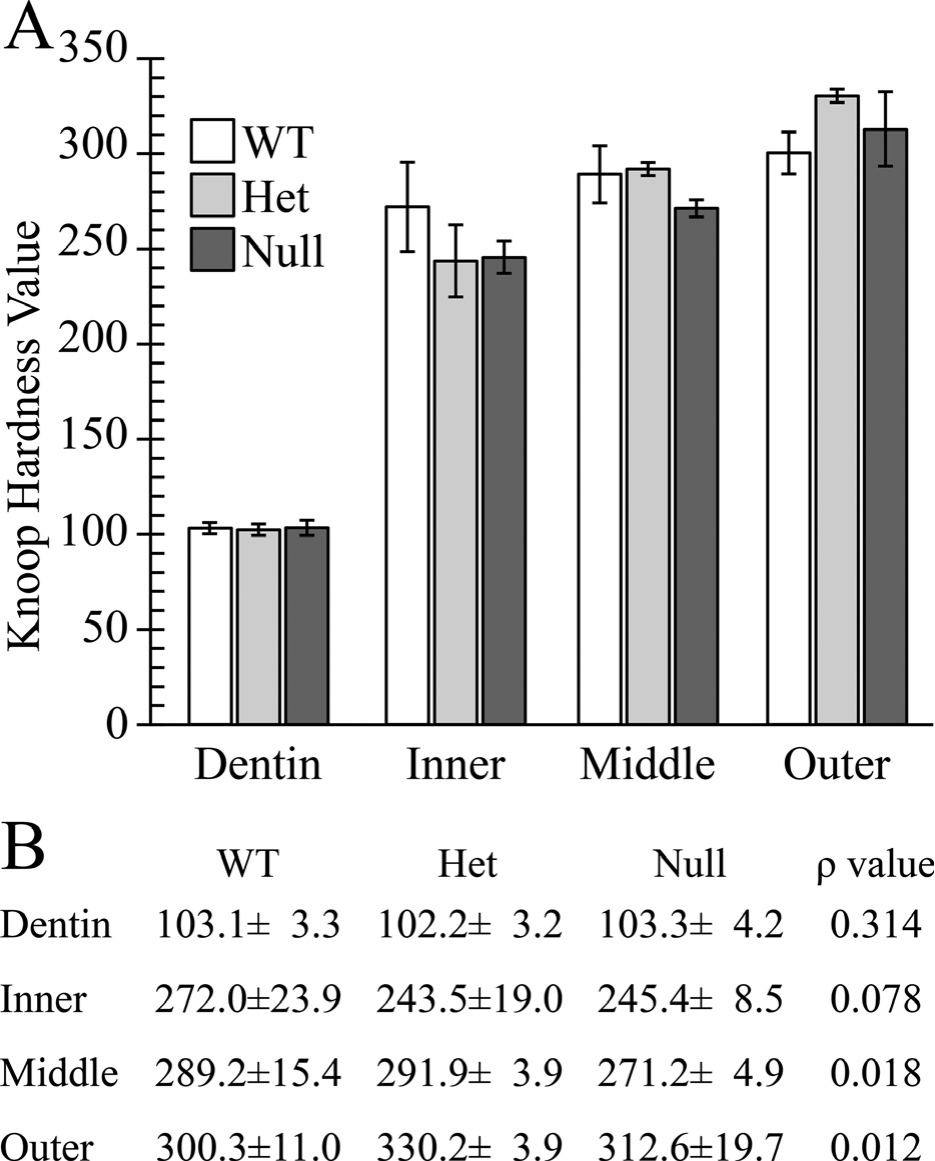
Hardness Plots. Indents in dentin, inner enamel, middle enamel, and outer enamel were made on 7‐week mandibular incisor cross sections from the level 8 of the alveolar crest (level 8). A: Plot of the average calculated Knoop Hardness Value for wild‐type (WT), *Fam83h* heterozygous (Het) and *Fam83h* Null mice for dentin and enamel (inner, middle and outer). The enamel at all levels was very hard (2.5 to 3× as hard as dentin) and increased going from the inner to outer enamel. B: Average calculated Knoop Hardness Values with standard deviations and *P* values. A *P* < 0.01 was considered significant. All *P* values were above 0.01 and did not support a hypothesis of significant hardness differences among the three genotypes.

In order to evaluate the enamel in the context of functionality, we performed Knoop hardness testing (KHT) of dentin, the inner enamel (near dentin), the middle enamel, and outer enamel (near the surface) of incisors sectioned at level 8 for each of the three genotypes. No significant difference of Knoop hardness numbers (KHNs) was observed in respective areas of enamel among *Fam83h* ^+/+^, *Fam83h* ^+/−^, and *Fam83h* ^−/−^ mice (Fig. 7).

Overall, there were no dental phenotypes detected by light microscopy, bSEM analyses, and hardness testing in either heterozygous or homozygous *Fam83h* null mice, except for a suggestion of a slightly reduced rate of incisor eruption.

### *β*‐Galactosidase reporter expression in developing teeth and other tissues

**Figure 8:**
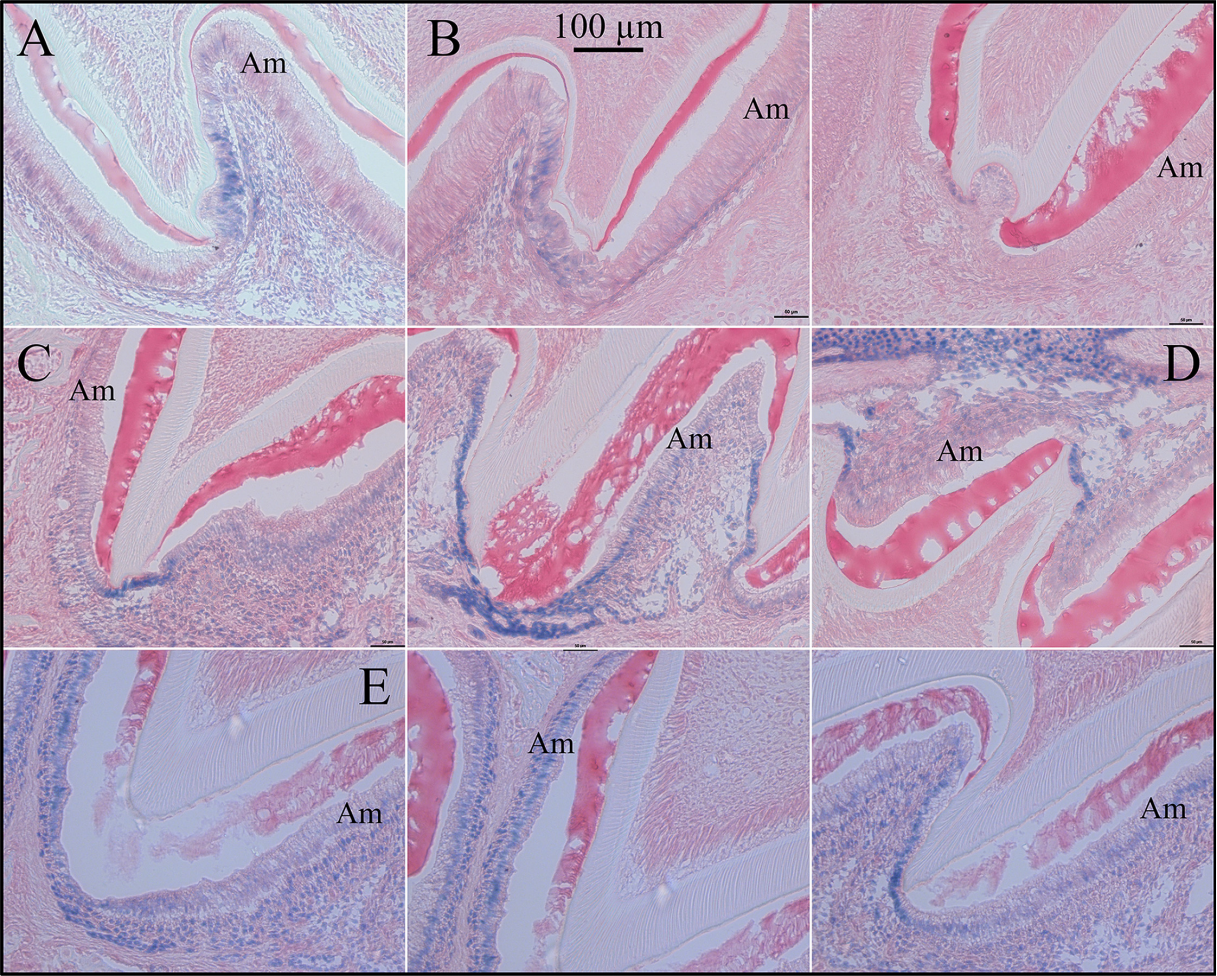
X‐gal Histostaining of *Fam83h*‐knockout/NLS‐*lac*Z‐knockin (null) developing molars. A: Cusp region of *Fam83h* null D5 maxillary second molar showing positive staining in epithelia covering the enamel‐free area. The first molar and incisor ameloblasts were negative or trace for X‐gal staining (Figs. S13–S14). B: Cusp region of *Fam83h* null D6 maxillary second molar showing weak/positive staining in epithelia covering the enamel‐free area. No other ameloblasts were positive in D6 molars or incisors (Fig. S14). C: Cusp region of *Fam83h* null D9 maxillary second molar showing positive staining in epithelia covering the enamel‐free area and weak staining in maturation stage ameloblasts (Fig. S15). D: Cusp region of *Fam83h* null D9 mandibular second molar showing positive staining in epithelia covering the enamel‐free area. E: Day 11 maxillary molars show positive histostaining for maturation stage ameloblasts.

To study the spatial and temporal pattern of *Fam83h* expression, we performed X‐gal staining on sections of developing molars at D5 (Figs. S12, S13), D6 (Fig. S14) D9 (Fig. S15), D11 (Fig. S16) and 7‐week incisors (Fig. S17), and many other organ tissues. The developing first molar of D5 *Fam83h* ^−/−^ mice showed detectable but very weak X‐gal histostaining over the enamel organ epithelium, including cells of stratum intermedium, stellate reticulum and outer enamel epithelium (Fig. 8A). The signal in ameloblasts, which were in the secretory stage, was sporadic and barely detectable. In contrast, the oral epithelium exhibited much stronger staining, compared to the dental epithelium. A similar staining pattern was also observed on the *Fam83h* ^−/−^ first molars at D6 (Fig. 8B) and D9 (Fig. 8C). However, the maturation stage ameloblasts as well as other epithelial cells of the enamel organ at D11 *Fam83h* ^−/−^ first molars were more evidently stained compared to those molars from earlier stages (Fig. 8D), although the signal was still generally weaker than that of oral epithelium. Interestingly, the epithelia at the enamel‐free zone over the cusps of developing molars consistently showed strong staining over sequential stages. However, on the same sections of these developing molars of D5, D6, D9, and D11 mice, the enamel organ epithelia of the incisors were all negative for staining. Furthermore, in contrast to the epithelial components of developing molars, no positive staining could be detected in odontoblasts, dental pulp cells, and surrounding bone tissues.

**Figure 9:**
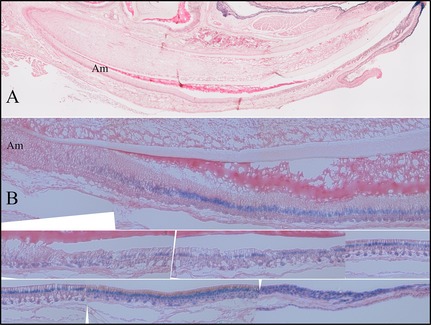
X‐gal Histostaining of *Fam83h*‐knockout/NLS‐*lac*Z‐knockin (null) mandibular incisors. A: Sagital section from D9 *Fam83h* ^+/−^ mouse. All Ameloblasts are unstained. B: Sagital section from 7‐week *Fam83h* ^+/−^ mouse. All Ameloblasts are stained positive.

We also performed X‐gal staining on longitudinal sections of D9 and 7‐week *Fam83h* ^+/−^ mandibular incisors (Fig. 9), which display sequential stages of enamel formation in a single tooth. In D9 *Fam83h* ^+/−^ mandibular incisor, while X‐gal staining was evident in oral epithelium, no positive signal was detected throughout the enamel organ of the whole incisor. In contrast, in the 7‐week *Fam83h* ^+/−^ mandibular incisor, the signal became detectable as early as the beginning of secretory stage when ameloblast differentiation was complete (Fig. S17). The staining stayed positive afterwards and got stronger gradually as the late maturation stage ameloblasts exhibited the strongest signal. Interestingly, unlike the staining pattern of the developing molars, the secretory and maturation stage ameloblasts of the 7‐week mandibular incisor were more prominently stained compared to other epithelial cells of the enamel organ. These results suggested that mouse *Fam83h* expression during amelogenesis varied in developing molars relative to the incisors. Also, incisors at younger (D9) and older (7 week) ages showed very distinct *lac*Z expression patterns. Moreover, we observed positive X‐gal staining in the junctional epithelia of erupted molars of D28 *Fam83h* ^+/−^ mice, but no signal was detected in other parts of the periodontium, namely cementum, periodontal ligament, alveolar bone, and gingival connective tissues (Fig. S18).

Positive X‐gal staining was also noted in many other tissues and organs with various intensities. In 7‐week *Fam83h* ^+/−^ mice, the skin epidermis (Fig. S19), hair follicles, tongue epithelia (Fig. S20), submandibular salivary glands (Fig. S21), thymus (Fig. S22), and urinary bladder (Fig. S23) exhibited moderate to strong staining, while oviducts (Fig. S24) and testis (Fig. S25) showed weak signal. No signal could be detected in the uterus (Fig. S26), ovary (Fig. S27), and prostate (Fig. S28 and S29), except some endogenous false‐positive staining that was also evident in wild‐type controls. Noticeably, epithelial cells, but not mesenchymal cells, were stained in all of the tissues and organs that we showed positive signals. Furthermore, in some tissues, a sporadic staining pattern over specific epithelial cells was noted, such as Sertoli cells in seminiferous tubules of testis.

### No evident histological abnormalities during amelogenesis in *Fam83h* null mice

**Figure 10:**
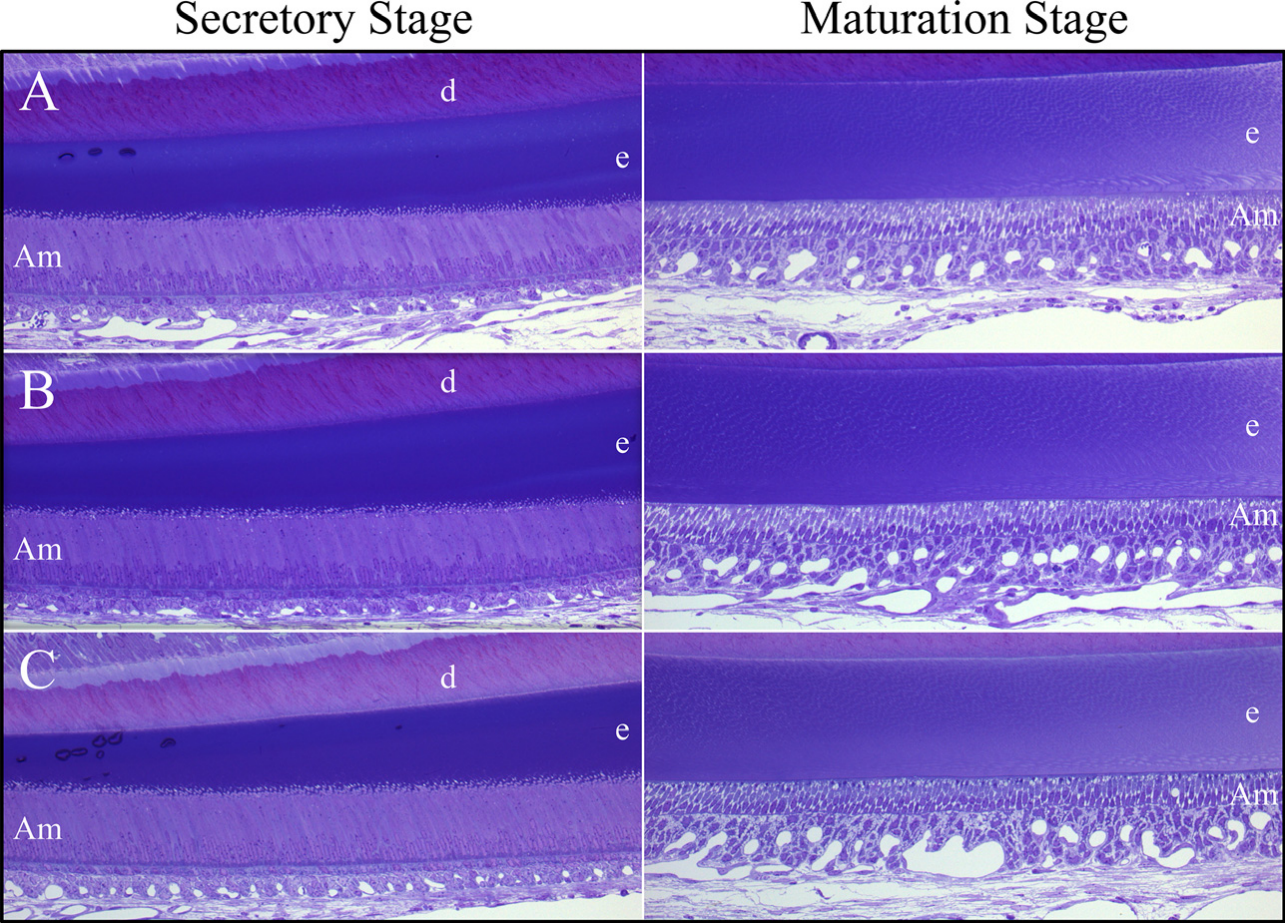
Histology of 7‐Week Mandibular Incisors. A: Wild‐type; B*: Fam83h* ^+/−^; *C: Fam83h* ^−/−^ mice. No histological differences were observed between the wild‐type mouse incisors and those developing in the *Fam83h* heterozygous or null mice.

We analyzed the histology of developing maxillary first molars at postnatal D5 (Fig. S30) and D11 (Fig. S31) and in 7‐week mandibular incisors (Fig. 10 and Fig. S32) to investigate the impact of FAM83H depletion on secretory and maturation stages of enamel formation. In all three genotypes (*Fam83h* ^+/+^, *Fam83h* ^+/−^, and *Fam83h* ^−/−^), the developing D5 first molars exhibited comparable basic morphology with typical tall‐columnar secretory stage ameloblasts and eosinophilic extracellular matrices accumulated in the enamel, suggesting that the enamel matrix secretion was not affected in *Fam83h* null mice. Similarly, the maturation stage ameloblasts of D11 first molars in all genotypes showed a comparable short‐columnar cell morphology, although there seemed to be more eosinophilic matrices in the enamel space of the *Fam83h* ^−/−^ molar, especially around the cusp tips. This suggested that the enamel maturation of *Fam83h* ^−/−^ molar was generally unaffected but might be delayed. Furthermore, the odontoblasts and developing dentin‐pulp complexes of both D5 and D11 first molars appeared comparably normal when *Fam83h* was ablated. Consistent with the findings from developing molars, the *Fam83h* ^−/−^ enamel organ of 7‐week mandibular incisor, including ameloblasts, and enamel matrices at secretory, transition, and maturation stages exhibited comparable morphology without any detectable abnormalities. While the size of *Fam83h* ^−/−^ maxillary incisor was generally smaller, this presumably reflected the overall smaller body size of *Fam83h* ^−/−^ mice. Together, the histological analyses of developing molars and incisors suggested that depletion of FAM83H has little impact on normal enamel development.

We also analyzed crude protein extracts from D11 first molars of three genotypes by SDS‐PAGE and immunoblotting with an antibody raised against recombinant mouse amelogenin (rM179) (Simmer et al. 1994). The results demonstrated that there was no significant difference in the amount of matrix proteins, mainly amelogenin, among *Fam83h* ^+/+^, *Fam83h* ^+/−^, and *Fam83h* ^−/−^ first molars at D11, which was consistent with our findings from molar histology (Fig. S33).

### FAM83H self‐interaction

In many pathological conditions, a mutant protein can exert a dominant negative effect by interacting with its wild‐type protein and lead to a loss of function. With the suspicion that the reported *FAM83H* mutations lead to a dominant negative effect to cause disease phenotypes, we hypothesized that FAM83H might interact with itself to form dimers or multimers. Furthermore, the N‐terminus of FAM83H contains a phospholipase D (PLD)‐like domain (cd09188) with a predicted structure similar to that of PLD. Since PLD has been shown to form dimers (Stuckey and Dixon 1999), it is possible that FAM83H can dimerize through its N‐terminal PLD‐like domain.

**Figure 11:**
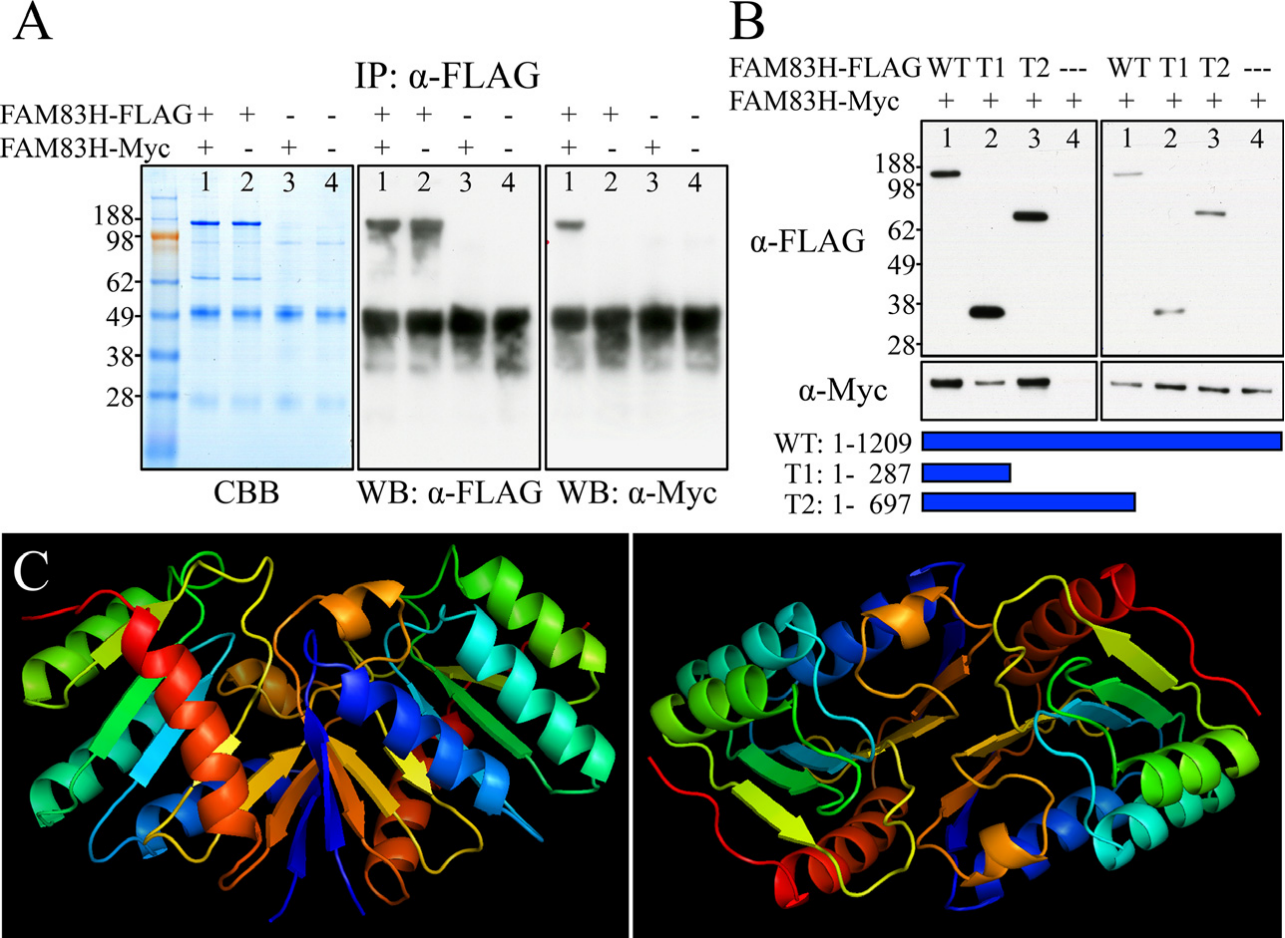
FAM83H Self Interactions. A: Pull‐down assay using FAM83H fused to FLAG (FAM83H‐FLAG) or Myc (FAM83H‐Myc) epitopes. FAM83H‐FLAG and FAM83H‐Myc were coexpressed in HEK293 cells. The cell lysate was immunoprecipitated (IP) with an *α*‐FLAG antibody and then fractionated by SDS‐PAGE and stained (left) with Coomassie Brilliant Blue (CBB) or (middle) immunoblotted with *α*‐FLAG or (right) *α*‐Myc antibodies. Lane 1: IP when both FAM83H‐FLAG and FAM83H‐Myc were coexpressed. Lane 2: IP when only FAM83H‐FLAG was expressed. Lane 3: IP when only FAM83H Myc was expressed. Lane 4: IP when only empty vector was expressed. The *α*‐FLAG IP pulled down both FAM83H‐FLAG and FAM83H‐Myc (~130‐kDa bands) indicating that FAM83H interacts with itself. (It would only have pulled down FAM83H‐FLAG if there were no interaction). The ~50‐kDa bands on the western blots (WB) are signals from heavy chain of rabbit IgG used for the *α*‐FLAG IP. B: Pull‐down assay coexpressing FAM83H‐Myc with three different FLAG‐tagged versions of FAM83H: Wild‐Type FAM83H (WT; amino acids 1–1209) and two C‐terminal truncated FAM83H proteins (T1, amino acids 1–287; T2, amino acids 1–697), or with empty vector (—). The IP products (left) and the initial cell lysates (right) were visualized by western blotting using *α*‐FLAG (top) or *α*‐Myc (bottom) antibodies. All three versions of FAM83H‐FLAG pulled down FAM83H‐Myc indicating that the first 287 amino acids of FAM83H are sufficient for self‐interactions. Bands of ~140‐kDa, ~36‐kDa, and ~75‐kDa are full‐length FAM83H, FAM83H^1‐287^, and FAM83H^1‐697^ respectively. C: Predicted human FAM83H^1‐287^ dimerization model. The interaction model was predicted by SPRING ON‐LINE software. The first 287 amino acids of human FAM83H were used as an input. The structure of phospholipase D from *S. typhimurium* (1byrA) was used as a modeling template. Left: Side view. Right: Top view.

To test this hypothesis, we first coexpressed FLAG‐tagged FAM83H and Myc‐tagged FAM83H in HEK293 cells and performed anti‐FLAG pull‐down assays using cell lysates. By immunoblotting with anti‐Myc tag antibody, we demonstrated that FLAG‐tagged FAM83H could pull down Myc‐tagged FAM83H, which suggested that FAM83H interacted with itself (Fig. 11A). We also tested this self‐interaction with two truncated FAM83Hs (FAM83H^1‐287^ and FAM83H^1‐697^). These truncations correspond to the shortest (FAM83H^1‐287^) and longest (FAM83H^1‐694^) disease‐causing FAM83H truncations in humans (Fig. S5). The results showed that both FLAG‐tagged FAM83H^1‐287^ and FAM83H^1‐697^ were able to pull down Myc‐tagged FAM83H, indicating that the N‐terminus of FAM83H (the first 287 amino acids) was responsible for the self‐interaction (Fig. 11B).

We also performed protein–protein interaction modeling using SPRING ON‐LINE, a template‐based algorithm for protein‐protein structure prediction (Guerler 2013). When the sequence of the first 287 amino acids of human FAM83H was used as an input, six interaction models were predicted. The one with the highest confidence score used phospholipase D from *S. typhimurium* as template (1byrA) (Fig. 11C) and supports the interpretation that FAM83H dimerizes through its N‐terminal PLD‐like domain (the first 287 amino acids of human FAM83H).

### FAM83H interactome

In addition to self (homomeric) interactions, a mutant protein might exert a dominant negative effect by competing with its wild‐type protein for (heteromeric) interactions with another protein that leads to a loss of function. Identifying FAM83H binding partners may also provide clues of its functions. Therefore, we aimed to identify the potential FAM83H interacting proteins using affinity purification combined with mass‐spectrometry (AP–MS) (Gingras et al. 2007; Dunham et al. 2012). We overexpressed FLAG‐tagged mouse FAM83H in HEK293 cells and performed immunoprecipitation with an anti‐FLAG antibody. The immunoprecipitates were then resolved by SDS‐PAGE, and six specific bands on the gel were sliced out and submitted for protein identification by mass‐spectrometry. By this means, all of the proteins that coimmunoprecipitated with the affinity‐purified FAM83H would be identified and the potential binding partners of FAM83H could be determined by subsequent analyses. A total of 143 proteins were identified by mass spectrometry, including 89 matches with a high confidence score (*P* < 0.05). The identified potential interacting proteins were involved in various cellular processes (Fig. S34).

### FAM83H and casein kinase 1 interaction

**Figure 12:**
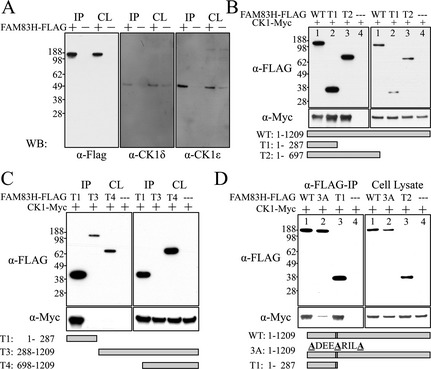
FAM83H‐CK1 interactions. A: Mouse FAM83H‐FLAG was expressed in HEK293 cells and immuno‐precipitated (IP) from cell lysates with *α*‐FLAG antibodies. The IP products and initial cell lysates (CL) were characterized by western blot analyses using *α*‐FLAG (left), *α*‐CK1*δ* (middle), and *α*‐CK1*ε* (right) antibodies. Bands at ~140‐kDa (FAM83H), ~49‐kDa (CK1*δ*), and ~47‐kDa (CK1*ε*) were observed. FLAG‐tagged FAM83H (+), but not controls (−), precipitated endogenous CK1*δ* and CK1*ε*, suggesting FAM83H interacts with these forms of CK1. B: CK1‐Myc (~50‐kDa) and one of three FAM83H‐FLAG proteins (WT: aa 1–1209, ~140‐kDa; T1: aa 1–287, ~36‐kDa; T2: aa 1–697, ~75‐kDa) or FLAG‐tag only (−) were expressed in HEK293 cells and *α*‐FLAG immunoprecipitated. The IP products (left) and the initial cell lysates (CL) were immunoblotted with *α*‐FLAG (top) or *α*‐Myc (bottom) antibodies. All three different‐length FAM83H‐FLAG proteins pulled down CK1‐Myc, suggesting that the first 287 amino acids of FAM83H are sufficient for the FAM83H‐CK1 interaction. C: Pull‐down assays were performed with three different truncated FAM83H‐FLAG proteins: T1: aa 1–287, ~36‐kDa; T3: aa 288–1209, ~110‐kDa; and T4: aa 698–1209, ~65‐kDa. While T1 can pull down CK1‐Myc, neither N‐terminal truncation (T3 or T4) can, confirming that the first 287 amino acids of FAM83H are necessary for FAM83H‐CK1 interactions. *D*: Pull down using FAM83H‐FLAG (WT), mutated FAM83H‐FLAG (3A) in which Phe^270^, Phe^274^, and Phe^278^ are all substituted with Ala (WT: F^270^‐X‐X‐X‐F^274^‐X‐X‐X‐F^278^ to 3A: A^270^‐X‐X‐X‐A^274^‐X‐X‐X‐A^278^). Compared to wild‐type (WT) FAM83H, FAM83H^3F^ has significantly reduced ability to pull down CK1, suggesting the F^270^‐X‐X‐X‐F^274^‐X‐X‐X‐F^278^ motif is critical for FAM83H‐CK1 interaction. This motif localizes in the red *α*‐helix of the human FAM83H^1‐287^ dimerization model shown on Fig. 11C.

Several members of casein kinase 1 (CK1) family were identified by AP‐MS analyses of the immunoprecipitates of FLAG‐tagged FAM83H (FAM83H‐FLAG). To validate this finding we analyzed FAM83H‐FLAG coimmunoprecipitates by western blot analyses. CK1*δ*, and CK1*ε* (CSNK1D and CSNK1E) were both immunodetected. FAM83H‐FLAG precipitated CK1*δ*, and CK1*ε* by FLAG antibody immunoprecipitation, while the control‐FLAG (without FAM83H) did not (Fig. 12A). We then sought to define the CK1 binding site on FAM83H.

Two constructs expressing truncated mouse FAM83H with N‐terminal FLAG tag (FAM83H^1‐287^ and FAM83H^1‐697^) were generated, and their ability to interact with myc‐tagged CK1*ε* (CK1‐myc) was tested (Fig. 12B). HEK293 cells were transfected with constructs expressing CK1‐Myc and FAM83H‐FLAGs of various lengths (WT, FAM83H^1‐287^, FAM83H^1‐697^, and empty vector control). The cell lysate from each group then underwent immunoprecipitation with FLAG antibody, and western analyses using anti‐CK1*ε* or anti‐Myc antibodies. All three of the variable‐length FAM83H‐FLAG truncations, but not the empty vector control, could pull down overexpressed CK1*ε*, meaning that the first 287 amino acids of FAM83H are sufficient to interact with CK1*ε*. We also tested the CK1‐binding ability of two N‐terminal truncated FAM83H‐FLAGs (FAM83H^288‐1209^ and FAM83H^698‐1209^) and showed that neither of these truncated proteins could pull down CK1*ε*, further demonstrating that the N‐terminus (amino acids 1–287) of FAM83H is necessary and sufficient for FAM83H‐CK1 interaction (Fig. 12C).

Searching for potential CK1 binding sites, Okamura et al. identified a conserved docking motif for CK1 binding (F‐X‐X‐X‐F) in many CK1‐interacting proteins (Okamura et al. 2004). Interestingly, in human FAM83H, there are four of such a sequence motif in its N‐terminus (F^247^‐X‐X‐X‐F^251^; F^270^‐X‐X‐X‐F^274^; F^274^‐X‐X‐X‐F^278^; and F^350^‐X‐X‐X‐F^354^) (Fig. S35), with the first three being highly conserved among the FAM83H orthologs during vertebrate evolution. The F^270^‐X‐X‐X‐F^274^‐X‐X‐X‐F^278^ motif is particularly conserved among the human FAM83 paralogs, suggesting that this motif might be a potential CK1 binding site in FAM83H. Noticeably, this motif is located at a highly conserved sequence area right before Ser^287^ of FAM83H, which corresponds to the most N‐terminal FAM83H truncation mutation reported so far. Therefore, we hypothesized that FAM83H interacts with CK1 through its F^270^‐X‐X‐X‐F^274^‐X‐X‐X‐F^278^ motif, and that all of the disease‐causing truncation FAM83H, including the shortest and the longest one, can interact with CK1. We mutagenized the three phenylalanines (F^270^, F^274^, and F^278^), potentially serving as a CK1 docking site in FAM83H, into alanines (FAM83H^3FA^) by site‐directed mutagenesis and evaluated this motif for its importance for CK1 binding. By using the same pull‐down assays, we demonstrated that site‐directed mutagenesis of F^270^, F^274^, and F^278^ to A^270^, A^274^, and A^278^, respectively, in FAM83H significantly attenuated its ability to interact with CK1*ε* (Fig. 12D). This result supported our hypothesis that FAM83H interacts with CK1 through its F^270^‐X‐X‐X‐F^274^‐X‐X‐X‐F^278^ motif near the end of its N‐terminal PLD‐like domain (aa 4–281).

### Mouse recombinant FAM83H phosphorylation by casein kinase 1 in vitro

The FAM83H‐CK1 interaction raises the possibility that FAM83H might be phosphorylated by CK1, since there are many predicted CK1 phosphylation sites with a specific D/E/pS‐X‐X‐Ser/Thr motif in FAM83H. In order to test if FAM83H is the substrate of CK1, we used bacterial‐expressed mouse recombinant FAM83H and performed an in vitro kinase assay. Incubated with *γ*‐^33^P‐ATP, the recombinant FAM83H showed much stronger radioactivity when CK1 was added, compared with a CK2 (casein kinase 2) and no kinase control, which demonstrated that FAM83H can be phosphorylated by CK1 in vitro (Fig. S36A).

We submitted the CK1‐phosphorylated mouse recombinant FAM83H (with cold ATP) for mass‐spectrometry to identify the exact CK1 phosphorylation sites in FAM83H. The results showed that most of the CK1‐phosphorylated Serines and Threonines were located at the C‐terminus of FAM83H (Fig. S36B).

### Altered subcellular localization of CK1 in FAM83H^1‐697^‐expressing cells

**Figure 13:**
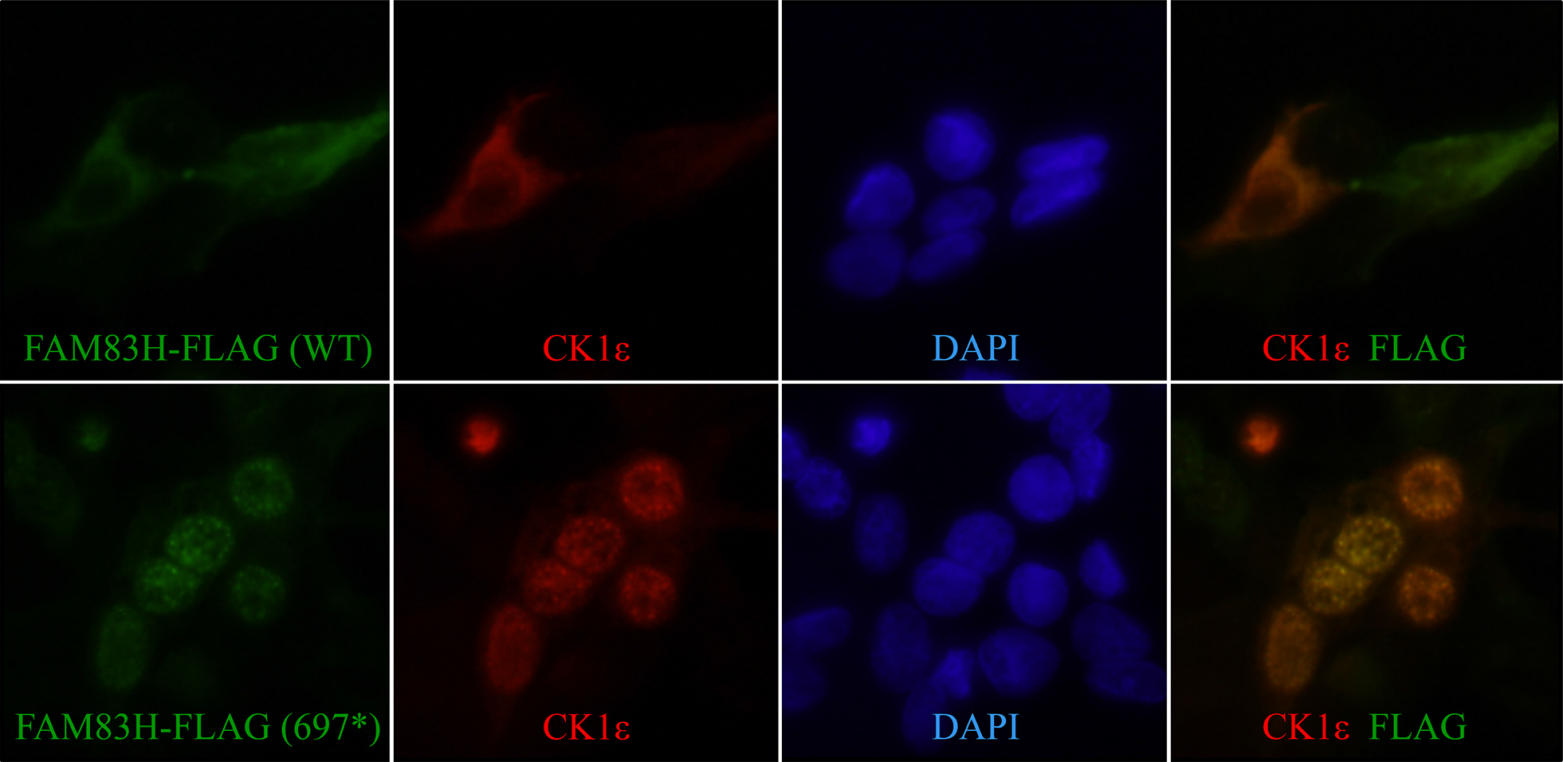
FAM83H and CK1*ε* immunocytochemistry. FLAG‐tagged full‐length (WT, FAM83H^1‐1209^) and truncated (697*, FAM83H^1‐697^) FAM83H proteins were expressed in HEK293 cells and immunostained with *α*‐FLAG (green) and *α*‐CK1*ε* (red) antibodies. *Top*: Both overexpressed WT FAM83H (green) and endogenous CK1*ε* (red) localize in the cytoplasm. *Bottom*: Unlike WT FAM83H, overexpressed FAM83H^1‐697^ (green) localized in the nucleus. Endogenous CK1*ε* (red) also mislocalized to the nucleus. Panels on the right show the superimposition of FLAG and CK1*ε* images. Blue: DAPI nuclear staining.

We also performed immunostaining with anti‐FLAG antibody in HEK293 cells overexpressing FLAG‐tagged full‐length FAM83H and FAM83H^1‐697^. Interestingly, while full‐length FAM83H showed localization in the cytoplasm, the FAM83H^1‐697^ localized in the nucleus. More interestingly, we observed altered intracellular localization of endogenous CK1*ε* in HEK293 cells overexpressing FAM83H^1‐697^. While CK1*ε* showed a diffuse cytoplasmic localization pattern when full‐length FAM83H was overexpressed, in the presence of FAM83H^1‐697^, CK1*ε* localized to the nucleus as FAM83H^1‐697^ itself did (Fig. 13).

## Discussion

There are now 22 different *FAM83H* mutations reported to cause human autosomal dominant hypocalcified amelogenesis imperfecta (ADHCAI) (Fig S1). All of these disease‐causing mutations are nonsense or frameshift mutations in the coding segment for Ser^287^ through Glu^694^ in the last *FAM83H* exon. In this study, we identified one previously reported (p.Gln452*) and two novel (p.Gln457*, p.Lys639*) *FAM83H* mutations that segregate with the ADHCAI phenotype. No other types of FAM83H defects, such as missense mutations, have been reported to be disease‐causing, which suggest that a loss of FAM83H function or haploinsufficiency is not a plausible mechanism of *FAM83H*‐associated ADHCAI. While nonsense mutations in earlier exons (Exons 2–4) of *FAM83H* have never been identified in patients with ADHCAI, many of these kinds of sequence variations have been found in the general population. For example, a nonsense sequence variant in Exon 3 of *FAM83H* (p.Gln201*; rs189033490), shows a minor allele frequency of 0.04% in the 1000 Genomes database and a 0.15% frequency in the NHLBI Exome Sequencing Project database. Introducing a premature termination codon in the early exons of *FAM83H* is presumed to generate a mutant transcript that undergoes nonsense mediated decay and results in a *FAM83H* null allele (Popp and Maquat 2013). Therefore, the presence of these variants in the general population indicates that a loss of half of the normal amount of FAM83H protein does not lead to a disease phenotype in humans, supporting the hypothesis that the enamel defects of ADHCAI are not caused by FAM83H haploinsufficiency. Conversely, a dominant negative effect or a “gain‐of‐function” from a truncated protein produced by *FAM83H* disease‐causing mutations seems more likely to underlie the pathological mechanism of ADHCAI.

When we generated the *Fam83h*‐knockout/*lacZ*‐knockin mice, we expected to find significant and obvious dental malformations in the null condition. However, this was not observed. We examined the enamel surfaces of incisors and molars under a dissecting microscope and by bSEM and found no apparent differences between the wild‐type, *Fam83h* ^+/−^, and *Fam83h* ^−/−^ mice. Enamel thickness and prism patterns were normal, indicating that the secretory stage of amelogenesis had proceeded normally. Enamel density, protein content and Knoop hardness were also comparable, indicating that the maturation stage had achieved its objectives. The histology of the ameloblasts in all stages of development, in molars and incisors, was virtually identical in all three genotypes and showed no signs of pathology. These findings lead us to the unexpected conclusion that FAM83H is not required for normal enamel development in mice. Previously it was shown that FAM83H overexpression in mice did not cause enamel defects (Kweon et al. 2013). These results further diminish the likelihood that human FAM83H truncation mutations cause enamel malformations through a haploinsufficiency mechanism.

The NLS‐*lac*Z reporter gene knocked into the *Fam83h* locus provided a useful tool to investigate the spatial and temporal pattern of *Fam83h* expression in mice. The NLS‐*lac*Z reporter showed no *Fam83h* expression in secretory stage ameloblasts in developing molars and incisors, except in the enamel‐free zone in the molars. Maturation stage ameloblasts were positive for *Fam83h* expression, but the levels were low and less than in other tissues, such as skin and hair follicles. Both secretory and maturation stage ameloblasts were positive in the mandibular incisor at 7‐weeks. The variability of *Fam83h* expression between molars, young incisors, and 7‐week incisors was unexpected, given that the enamel of all teeth is affected in human ADHCAI. These findings led us to the unexpected conclusion that FAM83H is not only not required for normal enamel development in mice, but that there may be differences in the patterns of FAM83H expression during tooth development between mice and humans.

The strongest X‐gal staining in the knockin mice was in the oral mucosa epithelia and skin epithermis, suggesting a potential function for FAM83H in epithelial cells. However, no overt abnormalities were found in these tissues of the *Fam83h* null mice, except mildly scurfy skin. More interestingly, the positive X‐gal staining in hair follicles not only explained the sparse and scruffy coat of *Fam83h* null mice, but also suggested an important role of FAM83H in hair follicle development and homeostasis. This also seems to be true for dogs. A 1 bp deletion in the critical exon 5 segment in *Fam83h* (Wang et al. 2013) caused autosomal recessive congenital keratoconjunctivitis sicca and ichthyosiform dermatosis (CKCSID; also known as “dry eye curly coat syndrome”) in the Cavalier King Charles Spaniel, with no apparent enamel malformations, although the affected dogs exhibited increased tartar formation and gingivitis that eventually required tooth extractions (Forman et al. 2012). It appears that the type of mutation that causes ADHCAI in humans does not cause an analogous condition in dogs, but instead causes a recessive condition (CKCSID) analogous to the phenotype in *Fam83h* null mice. Interestingly, we also found strong X‐gal staining in the junctional epithelia of erupted teeth (Fig. S18), suggesting a potential role of FAM83H in the epithelial attachment component of the periodontium. This finding might help explain the potential deep periodontal packets around *Fam83h* ^−/−^ incisors and molars with inserted coat hairs, although hair loss and early onset periodontal conditions have not been reported in patients with ADHCAI.

Consistent with the many *Fam83h* ESTs (expression sequence tags) found in extraoral tissues and organs of mice, we detected positive X‐gal staining in many organs, confirming a wide expression of *Fam83h*. However, despite this ubiquitous expression pattern, we only found positive FAM83H signal specifically in epithelial and not mesenchymal tissues in all of the mouse organs we examined (Figs. S18–S29), suggesting potential functions of FAM83H in epithelial cells. The postnatal lethality of *Fam83h* ^−/−^ mice around 2 weeks and the general weakness of the few survival null mice demonstrated an essential role of FAM83H in certain critical physiological functions. However, the finding that the null mice appeared to be normal at birth but usually died during the second week after birth suggested that FAM83H might not play significant roles in development of vital organs but might instead be required for the homeostasis of certain critical physiological functions. Further investigations are required to discern the cause of death in *Fam83h* null mice and to unravel the cellular functions of FAM83H. The ubiquitous expression of *Fam83h* also raised two interesting questions: Is it lethal in humans to have both *FAM83H* alleles mutated or ablated? Also, why do patients carrying a disease‐causing *FAM83H* mutation exhibit only enamel defects without clinically detectable abnormalities in other organ systems?

The absence of enamel defects in *Fam83h* null mice further supports the interpretation that the pathological mechanism for ADHCAI in humans is a gain‐of‐function. A dominant negative effect is caused by a mutation whose gene product adversely affects the normal, wild‐type gene product within the same cell, usually by dimerizing (combining) with it. That is, the mutant protein causes a disease phenotype by interfering with the normal functions of the wild‐type protein expressed from the unmutated allele. When a mutant allele is expressed at the same level as the wild‐type allele and the products readily form nonfunctional heterodimers, the quantity of functional dimers is only one/fourth that of the wild‐type. This is significantly less than the 50% levels of wild‐type protein in heterozygous conditions absent a dominant negative effect. However, our Fam83h null mice produced no FAM83H whatsoever and did not show an enamel phenotype. We detected no significant decrease in enamel hardness in the *Fam83h* null mice relative to the wild‐type, whereas humans with a single defective (c.1354C>T, p.Q452*) *FAM83H* allele exhibited soft enamel that was only 17% as hard as the normal control (Hyun et al. 2009). Therefore, the absence of overt enamel phenotypes in *Fam83h* ^−/−^ mice actually suggested that a “gain‐of‐function” (neomorph) rather than a dominant negative effect (antimorph) from the mutant FAM83H protein causes the enamel defects in human ADHCAI. The truncated FAM83H from the mutant allele appears to impair normal enamel development by executing certain “toxic” effects in ameloblasts rather than by blocking the normal functions of the wild‐type FAM83H expressed from the other allele. Therefore, in order to investigate the pathological mechanism of ADHCAI, a mouse model knockin analogous to a human disease‐causing *FAM83H* mutation should be established. However, potential species differences between human and mice in the role of FAM83H during amelogenesis cannot be discerned in this study but should not be overlooked.

The lack of documented individuals with homozygous *FAM83H* nonsense mutations in either general population or patients with ADHCAI, despite the relatively high allele frequency of these mutations, suggests that mutations in both *FAM83H* alleles might be lethal and selected against. Furthermore, based upon the normal enamel formation in *Fam83h* ^−/−^ mice, we hypothesized that FAM83H might not be essential for enamel development and that a “gain‐of‐function” (neomorph) rather than a dominant negative effect (antimorph) from the mutant FAM83H protein underlies the enamel defects in human ADHCAI. However, in contrast, with the postnatal lethality of *Fam83h* ^−/−^ mice, it is apparent that certain critical physiological functions are dependent upon FAM83H. Given it is true that a mutant truncated FAM83H protein does not interfere the normal functions of wild‐type FAM83H, it is possible that one normal *FAM83H* allele might be sufficient for maintaining its functions in other organ systems. Therefore, patients with ADHCAI exhibit only enamel defects without systemic involvement. However, why these tissues are less sensitive to the “toxic” effects from mutant FAM83H compared to developing enamel still remains to be elucidated.

Using biochemical approaches we demonstrated that FAM83H dimerizes through interactions within its predicted PLD‐like domain (N‐terminal 287 amino acids). Although this finding suggests that the mutant truncated protein might interact with the wild‐type protein and prevent it from functioning (a dominant negative effect), our *Fam83h* ^−/−^ mice did not exhibit enamel defects, which contradicts this hypothetical disease mechanism of ADHCAI. However, the evolutionary conservation of FAM83H PLD‐like domain still suggested that the dimerization of FAM83H proteins through this domain might be important for its functions. We also demonstrated that FAM83H interacts with CK1 and that the first 287 amino acids of FAM83H are necessary and sufficient for this interaction. A specific sequence motif, F^270^‐X‐X‐X‐F^274^‐X‐X‐X‐F^278^, located at an evolutionarily conserved region N‐terminal to Ser^287^, seems to play a critical role in the FAM83H‐CK1 interaction. This finding is further supported by a study of the CK1 interactome (Kategaya et al. 2012) and a study about FAM83H in colorectal cancer (Kuga et al. 2013). It is known that the subcellular localization of CK1 is an important factor in its functional regulation (Knippschild et al. 2005). Therefore, our finding that FAM83H interacts with CK1 via distinct motifs suggests that FAM83H may function as a scaffold protein to bring CK1 to its physiological locations. If this hypothesis is true, the *FAM83H* disease‐causing mutations (truncation from FAM83H^1‐287^ to FAM83H^1‐694^) produce a truncated protein that binds CK1, but fails to localize CK1 properly (directs it to the nucleus) and possibly altered the normal pattern of protein phosphorylation in the cell. Alternatively, the disease phenotype might result from an adverse pathological effect of mislocalized truncated FAM83H in the nucleus. This mechanism might explain the dominant pattern of defects in humans with ADHCAI, but a simple loss of FAM83H function (possibly to correctly localize CK1) might explain the systemic conditions observed in the *Fam83h* null mice, which are not observed in patients with ADHCAI.

We also demonstrated that recombinant mouse FAM83H can be phosphorylated by CK1 in vitro, and most of the phosphorylation sites are located within the C‐terminal region of FAM83H, although most of these phosphorylated serines and threonines are not evolutionarily conserved. Therefore, at this stage, we are not sure if FAM83H phosphorylation by CK1 is functionally or structurally significant. However, there are two amino acid sequence areas (1025–1055 and 1114–1139 in human FAM83H) near the C‐terminus of FAM83H that show high sequence conservation during vertebrate evolution (Fig. S36), suggesting that these regions may be important for FAM83H function. Given that FAM83H is a scaffold protein as we suspect, these two sequences areas may serve as protein docking sites. Using additional truncated FAM83H proteins to define the functions of these conserved domains seems to be the next logical pursuit.

## Materials and Methods

### Ethics statement

The human study protocol and consents were reviewed and approved by the IRB Committee at the National Taiwan University Hospital, Taipei, Taiwan, the Institutional Review Board at the University of Michigan, and the Ethics Committee of University of Istanbul, Turkey. Study participants signed appropriate written consents after explanation and discussion of their contents. All procedures involving animals were reviewed and approved by the UACUC committee at the University of Michigan and all relevant guidelines were followed.

### Mutational analyses

Peripheral whole blood (5 mL) or saliva (2 mL) was obtained from recruited individuals and genomic DNA was isolated using the QIAamp DNA Blood Maxi Kit (51194; Qiagen, Valencia, CA) or Saliva DNA Collection, Preservation, and Isolation Kit (RU35700; Norgen Biotek Corporation; Thorold, Canada), respectively. The quality and quantity of the extracted DNA samples were determined by spectrophotometry at OD_260_ and OD_280_.

Genomic DNA from the proband of Family 1 was characterized by whole‐exome sequencing (Yale Center for Genome Analysis, West Haven, CT, USA) in which the procedure was previously described (Choi et al. 2009). In brief, the genomic DNA was captured with NimblGen v2.0 exome capture reagent (Roche/NimblGen Incorporation; Madison, WI) and sequenced with Illumina HiSeq 2000for 75 base paired‐end reads. Reads were aligned to human reference genome hg19 using ELAND v2. Single nucleotide variants and short insertions and deletions (indels) were called using SAM tools. The called variants were annotated using an in‐house script. The annotated results were first inspected to search for potential disease‐causing sequence variations in the known candidate genes for syndromic and non‐syndromic AI. The identified *FAM83H* mutation was further validated by Sanger sequencing.

Genomic DNAs from the probands of Family 2 and 3 were screened for potential *FAM83H* disease‐causing mutations by analyzing the *FAM83H* Exon 5. Specifically, the coding region of Exon 5 was amplified by using six pairs of primers (Fig. S37). The amplification products were purified and characterized by Sanger sequencing at the University of Michigan DNA Sequencing Core. The sequencing data were then compared to the human reference sequence, and sequence variants called and evaluated. *FAM83H* c.DNA and genomic changes were numbered with respect to the National Center for Biotechnology Information (NCBI) human FAM83H mRNA reference sequence: NM_198488.3 (numbered from the first nucleotide of the *FAM83H* translation initiation codon in exon 2) and genomic reference sequence NG_016652.1 (numbered from the first nucleotide of the reference sequence).

### Generation of knockin mice

The knockin construct was designed and the mice (species: *Mus musculus*, strain C57BL/6) were generated by Ozgene (Perth, Australia). The generation of knockin (KI) gene precisely replaced the *Fam83h* coding sequence from Exon 2 to Exon 5 with the coding sequence of a *lac*Z (*β*‐galactosidase) reporter containing a mouse nuclear localization signal (NLS) and two downstream polyadenylation signals (pA), followed by a phosphoglycerine kinase (PGK) promoter driving a neomycin (Neo) selection marker in reverse direction. The PGK‐Neo coding sequence was flanked by FRT sites and later deleted by flippase (Flp). The deleted *Fam83h* and inserted NLS‐*lac*Z sequences are provided in Fig. S7.

### Physical assessment and photography

Heterozygous mutant (*Fam83h* ^*+/−*^) mice were mated to generate three mouse *Fam83h* genotypes (+/+, +/−, and −/−), which were then evaluated for their physical appearance, activity, growth rate, size difference, food intake, and fertility. Although the pups of three genotypes were born at the expected Mendelian ratio, almost all the *Fam83h* ^*−/−*^ mice died within 2 weeks after birth with only a few surviving to 7 weeks. For gross evaluation of the dental enamel, mice were put under anesthesia using isofluorane, and the incisors were inspected under a dissection microscope. 7‐week‐old mice were sacrificed and fixative‐perfused with 4% paraformaldehyde (PFA) or fixative‐perfused with 2.5% glutaraldehyde. The mandibles were removed and sliced through the mental symphysis with a razor blade to generate hemimandibles. These were carefully dissected free of soft tissues under a stereoscopic microscope using tissue forceps and a spoon excavator. The hemimandibles were submerged in 1% NaClO for 5 min, rinsed, air dried, and photographed using a Nikon SMZ1000 dissection microscope equipped with a Nikon digital camera DXM1200 (Melville, NY).

### X‐gal staining

The processes of tissue preparation, cryosectioning, and X‐gal histostaining were previously described (Simmer et al. 2011). Briefly, mouse heads of D5, D6, D9, D11, D28, and 7‐week mice were harvested and fixed with 4% PFA overnight at 4°C. The tissues were then decalcified at 4°C by immersion in 1 L of 4.13% disodium ethylenediaminetetraacetic acid (EDTA, pH 7.3) to reach the soft consistency suitable for sectioning. After cryoprotection and embedding in OCT, the tissue blocks were cryosectioned at 8‐*μ*m thickness, and the slides were stored at −80°C. For staining, slides were treated with glutaraldehyde fixative (0.1 mol/L HEPES, 1.25 mmol/L EGTA, 2 mmol/L MgCl_2_, and 0.5% glutaraldehyde, pH 7.3) and then washed with 0.1 mol/L HEPES and 2 mmol/L MgCl_2_ (pH 7.3) 3 × for 5 min. The slides were stained with X‐gal solution (0.1 mol/L HEPES, 1 mmol/L MgCl_2_, 5 mmol/L potassium ferrocyanide, 5 mmol/L potassium ferricyanide, 2% Triton X‐100, and 1 mg/mL X‐gal substrate; pH 8.0) for 5 h at 45°C and then washed several times in PBS, and counterstained with 0.1% (w/v) Nuclear Fast Red, coverslipped with Aquamount, and imaged using a Nikon Eclipse TE300 inverted microscope.

### Histological staining and analyses

For histology of developing molars, day 5 and 11 mouse heads were quickly dissected free of skin, cut in half, and immersed in 4% PFA fixative overnight at 4°C, washed in PBS 4–5 times (every 0.5–1 h) at 4°C, and decalcified at 4°C by immersion in 1 L of 4.13% disodium EDTA (pH 7.3) with agitation. The EDTA solution was changed every other day for 19–21 days for D11 mice, and 30 days for D14 mice. The samples were washed in PBS at 4°C 4–5 times (every 0.5–1 h) followed by one overnight wash. The samples were dehydrated using a graded ethanol series followed by xylene, embedded in paraffin, and sectioned at 5 *μ*m thickness. For staining, the sections were rehydrated and stained with hematoxylin and eosin (H&E) stain.

### Protein extraction and analyses from mouse molars

The detailed protocol of protein extraction from mouse molars and the analyses were previously described (Wang S‐K et al. 2015). Briefly, D14 first molars were incubated in 1 mL of 0.17 N HCl/0.95% formic acid for 2 h at 4°C. After the undissolved materials were removed, the crude protein extract was buffer exchanged with 0.01% formic acid. The concentrate containing proteins extracted from four molars was raised back to 250 *μ*L of 0.01% formic acid and used for subsequent SDS‐PAGE, Coomassie Brilliant Blue (CBB) staining, and amelogenin immunoblotting. The amount of protein applied per lane for SDS‐PAGE was normalized for each genotype on a per‐tooth basis. For CBB staining, 3/21 of a tooth was applied per lane; and for immunoblotting, 1/21 of a tooth was used. A polyclonal rabbit anti‐full‐length mouse recombinant amelogenin antibody (rM179; 1:2000) was used for immunoblotting (Simmer et al. 1994).

### Backscattered scanning electron microscopy

The procedures of backscattered scanning electron microscopy (bSEM) were described previously (Smith et al. 2011). Soft tissue was removed from the left and right hemimandibles of 7‐week‐old mice, and cross‐sectioned at 1 mm increments from the basal (growing) end of the incisors, and imaged by bSEM. For whole incisor surface imaging, the bony soft tissue and bony caps covering the mandibular incisors were carefully removed, and examined at 50 × magnification with a Hitachi S‐3000N variable pressure SEM using the backscatter mode at 25 kV and 20 pascal pressure.

For molar surface SEM, the molars were prepared as follows: The D14 hemimandibles were submerged in 4% PFA overnight, carefully dissected of soft tissues under a stereoscopic microscope, submerged in 1% NaClO for 5 min, rinsed, and acetone dehydrated (30%, 50%, 70%, 80%, 90%, and 100%). The hemimandibles were mounted on metallic stubs using conductive carbon cement and carbon coated to increase conductivity and examined using a Hitachi (Century City, Los Angeles, CA) S‐3000N variable pressure SEM using the backscatter mode.

### Microindentation hardness evaluation

The procedures for tissue preparation and microhardness testing were previously described (Wang S‐K et al. 2015). In brief, hemimandibles from 7‐week‐old mice were cleaned free of soft tissue and embedded in Epon resin following graded acetone dehydration. After polymerization at 65°C, the incisors were cross‐sectioned at the level of the crest of the alveolar bone close to where the incisor erupts into the mouth, about 8 mm from the apex of the incisor. The sectioned hemimandibles were reembedded in Castolite AC (Eager Polymers, Chicago, IL) using 25‐mm SteriForm molds (Struers Inc., Westlake, OH), allowed to harden overnight, and polished.

Microhardness testing was performed using a LM247AT microhardness tester (Leco Corp., St. Joseph, MI) with a load of 25 g for 10 sec with a Knoop tip to obtain a Knoop hardness number (KHN). Measurements were made at 500 × magnification. Indentations were placed in the outer, middle, and inner enamel as well as the dentin as a control reading for a total of four indentations per row. This series was performed three times in each animal, for a total of twelve points per animal. Hardness Data points were treated as independent, unweighted numbers and subjected to one‐way ANOVA than Tukey's HSD test using calculation spreadsheet at http://vassarstats.net/anova1u.html. Statistical significance was determined at *P* < 0.01.

### Expression constructs

The mouse *Fam83h* coding region was subcloned into pCMV‐Tag4A and pCMV‐Tag5A vectors (211174, 211175; Agilent Technologies; Santa Clara, CA.) to express C‐terminal FLAG‐tagged and Myc‐tagged mouse FAM83H. These two constructs were used for FAM83H self‐interaction experiments. The pCMV‐Tag4A‐*Fam83h* was also used to overexpress FLAG‐tagged FAM83H for affinity purification‐mass spectrometry (AP‐MS) experiment.

Seven constructs to express different domains, truncations, and mutations of FAM83H protein were generated. They are pCMV‐Tag2B‐*Fam83h*, pCMV‐Tag2B‐*Fam83h* ^*1‐287*^, pCMV‐Tag2B‐*Fam83h* ^*1‐657*^, pCMV‐Tag2B‐*Fam83h* ^*1‐697*^, pCMV‐Tag2B‐*Fam83h* ^*288‐1209*^, pCMV‐Tag2B‐*Fam83h* ^*698‐1209*^, and pCMV‐Tag2B‐*Fam83h* ^*3FA*^. The pCMV‐Tag2B‐*Fam83h* construct encodes a full‐length mouse FAM83H protein (1209 amino acids). The pCMV‐Tag2B‐*Fam83h* ^*1‐287*^, pCMV‐Tag2B‐*Fam83h* ^*1‐657*^, and pCMV‐Tag2B‐*Fam83h* ^*1‐697*^ constructs were made by introducing three respective premature stop codons into pCMV‐Tag2B‐*Fam83h* construct to express three truncated FAM83H proteins (1–287, 1–657, and 1–697 amino acids). The pCMV‐Tag2B‐*Fam83h* ^*288‐1209*^ and pCMV‐Tag2B‐*Fam83h* ^*698‐1209*^ constructs were designed to express two different‐length C‐terminal domain of FAM83H protein (288–1209 and 698–1209 amino acids). The pCMV‐Tag2B‐*Fam83h* ^*3FA*^ construct was generated by introducing three site‐directed mutations into pCMV‐Tag2B‐*Fam83h*, which encodes a full‐length FAM83H protein with three Phenylalanine‐to‐Alanine substitutions (p.Phe270Ala, p.Phe274Ala, and p.Phe278Ala). All the encoded FAM83H proteins contain an N‐terminal FLAG tag. These six constructs were used for protein pull‐down assays to study FAM83H protein‐protein interaction.

Constructs expressing 6xMyc‐tagged CK1*δ* and CK1*ε* (pcDNA3‐CK1*δ*‐6xMyc, pcDNA3‐CK1*ε*‐6xMyc) were gifts from Dr. Ying‐Hui Fu (University of California San Francisco) (Kategaya et al. 2012).

### Cell culture and plasmid transfection

Human HEK293 cells were cultured in Dulbeccos modified Eagle medium (DMED) (11995; Gibco^®^ by Life Technologies; Grand Island, NY) with 10% (v/v) fetal bovine serum (FBS) (16000; Gibco^®^ by Life Technologies) in a 5% CO_2_ humidified culture incubator. Cells were regularly passaged when reaching 95–100% confluency.

For transient plasmid transfection, cells in 2 mL DMEM were plated on six‐well plates the day before transfection so that the cell confluency could reach 75–80% on the day of transfection. 4 *μ*g of plasmid in 10 *μ*L of Lipofectamine^®^ 2000 (11668; Invitrogen^™^ by Life Technologies) was diluted with 500 *μ*L of Opti‐MEM^®^ I reduced serum medium (31985; Gibco^®^ by Life Technologies), and incubated for 20 min at room temperature. The plasmid/Lipofectamine^®^ 2000 complexes were then added to the culture media. After 6‐h incubation, the culture media were changed to the regular media without transfection complexes, and the cells were further cultured for 42 h before harvested.

### Immunoprecipitation (IP)

Fourty eight hour following transfection, cells were washed twice with cold phosphate‐buffered saline (PBS) and, for each well of six‐well plates, lysed with 500 *μ*L NP40 cell lysis buffer (FNN0021; Novex^®^ by Life Technologies) with 1 mmol/L phenylmethylsulfonyl fluoride (PMSF) (P7626; Sigma‐Aldrich; St. Louis, MO) and 1 × protease inhibitor cocktail (P2714; Sigma‐Aldrich). After 30 min lysis, the lysates were collected and centrifuged at 15,000 rpm ~21,000 *g* for 10 min. The supernatants were then used for subsequent analyses.

Dynabeads^®^ protein A immunoprecipitation kit (10006D; Novex^®^ by Life Technologies) was used for all the immunoprecipitation experiments. The experimental procedure followed the protocol provided by the manufacturer. In brief, anti‐FLAG antibody (1:20; F7425; Sigma‐Aldrich) was incubated with protein A‐attached Dynabeads for 30 min. The antibody/Dynabeads complexes were then mixed with the cell lysate and incubated for another 30 min at room temperature. After washed twice, the immunoprecipitates were eluted and assayed for SDS‐PAGE and immunoblotting.

### Western blot analysis

Regular immunoblotting was performed for protein detection following SDS‐PAGE electrophoresis. The primary antibodies used for western blot analysis include: anti‐FLAG^®^ (F7425) (1:2000), anti‐FLAG^®^ M2 (F1804) (1:4000), anti‐c‐Myc (C3956) (1:2000) from Sigma‐Aldrich (St. Louis, MO); anti‐Myc Tag, clone 4A6 (05‐724) (1:4000) from Millipore Corporation (Billerica, MA); anti‐ CK1*α*1 [EPR1961(Mendoza et al. 2007)] (ab108296) (1:2000), anti‐CK1*δ* [AF12G4] (ab85320) (1:4000), anti‐CK1*ε* [AF6C1] (ab82426) (1:2000) from abcam^®^ (Cambridge, MA); anti‐HaloTag^®^ (G9211) (1:1000) from Promega Corporation (Madison, WI). Two secondary antibodies were used: ECL anti‐rabbit IgG (NA934V; GE Healthcare; Little Chalfont, U.K.) and anti‐mouse IgG+IgM HRP (ab47827; abcam^®^; Cambridge, MA).

### Protein‐protein interaction modeling

SPRING ON‐LINE (http://zhanglab.ccmb.med.umich.edu/spring/) (Guerler 2013) from Zhang laboratory at University of Michigan was used for modeling potential FAM83H dimerization. Sequence of the first 287 amino acids (PLD‐like domain) from human FAM83H protein was used for both query sequence A and query sequence B.

### Affinity purification combined with mass spectrometry (AP‐MS)

FLAG‐tagged mouse FAM83H protein was overexpressed by transiently transfected HEK293 cells on a 10‐cm Petri dish. The cell lysate from all the harvested cells underwent immunoprecipitation (with anti‐FLAG^®^ antibody), and the immunoprecipitate was further fractionated with SDS‐PAGE, as described in the above sections. After Coomassie brilliant blue staining, six specific protein bands were sliced out and submitted to Keck Biotechnology Resource Laboratory at Yale University, where trypsinization, LC‐MS/MS mass spectrometry for protein identification, and subsequent data analysis were performed.

### In vitro kinase reaction

For each kinase reaction, 0.2 mg purified mouse recombinant FAM83H was incubated with 5000 units of casein kinase 1 (P6030S), casein kinase 2 (P6010S; New England BioLabs^®^; Ipswich, MA), or no enzyme and 0.05 *μ*Ci ^33^P‐ATP in a total reaction volume of 20 *μ*L. The reactions were conducted at 30°C for 60 min and subsequently fractionated with SDS‐PAGE of Novex^®^ 4–20% Tris‐Glycine protein gels (EC60255BOX; Novex^®^ by Life Technologies). After drying, the gel was exposed to a film for 20 min.

For determination of CK1 phosphorylation sites on FAM83H protein, mass spectrometry was used. The kinase reaction was conducted using above‐mentioned method except that non‐radioactive cold ATP was used. The reaction was fractionated with SDS‐PAGE. After Coomassie Brilliant Blue staining, the band of FAM83H was sliced out and submitted to Keck Biotechnology Resource Laboratory at Yale University, where trypsinization, LC‐MS/MS mass spectrometry for protein posttranslational modification identification, and subsequent data analysis were performed.

### Immunocytochemistry (ICC)

HEK293 cells were cultured in Lab‐Tek chamber slides (1 chamber) with cover (70360‐12; Electron Microscopy Sciences; Hatfield, PA) and transfected with pCMV‐Tag2B‐*Fam83h*, pCMV‐Tag2B‐*Fam83h* ^*1‐697*^, or control empty vector. After 18 h, the cells were fixed with 100% methanol for 15 min at −20°C, washed with PBS buffer for three times. Following blocking with 5% sheep serum (S22; Millipore Corporation; Billerica, MA) in PBT buffer (0.1% Triton X‐100 in PBS buffer) for 30 m at room temperature, anti‐FLAG^®^ antibody (1:200; F7425; Sigma‐Aldrich; St. Louis, MO) and anti‐CK1*ε* antibody (1:200; ab82426; abcam^®^; Cambridge, MA) were applied. After overnight incubation of primary antibody at 4°C, the cells were washed with PBS buffer for 15 min and then incubated for 30 min at room temperature in solutions containing anti‐rabbit IgG secondary antibody conjugated with Alexa Fluor 488 (1:500; A‐11008; Molecular Probes^®^ by Life Technologies) and anti‐mouse IgG1 secondary antibody conjugated with Alexa Fluor 594 (1:500; A‐21125; Molecular Probes^®^ by Life Technologies). The slides were then rinsed in PBS buffer for 15 min, mounted with ProLong^®^ Gold antifade reagent with DAPI (P‐36941; Molecular Probes^®^ by Life Technologies), and examined under a Leica DM5000B fluorescence microscope.

## Supplementary Material

Supplementary Material is available at MGGM online.

## Funding

This study was supported by NIDCR/NIH research grants DE019622 and DE015846, by a National Research Foundation of Korea (NRF) grant funded by the Korea government (2014R1A2A1A11049931).

## Conflict of Interest

None declared.

## Supporting information


**Figure S1.** *FAM83H* disease‐causing mutations.
**Figure S2.** Family 1. Unaffected Brother (II:2).
**Figure S3.** Family 1. Unaffected Father (I:1).
**Figure S4.** Family 1. Unaffected Mother (I:2).
**Figure S5.** Family 2 Chromatograms.
**Figure S6.** *Fam83h*‐Knockin Construct and Genotyping Strategy.
**Figure S7.** Mouse *Fam83h* Wild‐Type and NLS‐*lac*Z‐Knockin Sequences.
**Figure S8.** Wild‐type and *Fam83h* null mice at 7 weeks.
**Figure S9.** bSEM Images of Manibular Incisor Cross Sections at 7 weeks (lower magnification).
**Figure S10.** bSEM Images of Manibular Incisor Cross Sections at 7 weeks (higher magnification).
**Figure S11.** bSEM Images of Manibular Incisor Cross Sections at 7 weeks (highest magnification).
**Figure S12.** *Lac*Z Histochemistry of Developing PN5 *Fam83h* ^+/−^ Mouse Teeth.
**Figure S13.** *Lac*Z Histochemistry of Developing PN5 *Fam83h* null Mouse Teeth.
**Figure S14.** *Lac*Z Histochemistry of Developing PN6 *Fam83h* null Mouse Teeth.
**Figure S15.** *Lac*Z Histochemistry of Developing PN9 *Fam83h* null Mouse Teeth.
**Figure S16.** *Lac*Z Histochemistry of Developing PN11 *Fam83h* null Mouse Teeth.
**Figure S17.** *Lac*Z Histochemistry of 7‐Week *Fam83h* ^+/−^ Mandibular Incisors.
**Figure S18.** *Lac*Z Histochemistry of D28 *Fam83h* ^+/−^ Dental Papilla.
**Figure S19.** Histology and *Lac*Z Histochemistry of PN5 Perioral Skin.

**Figure S20.** *Lac*Z Histochemistry of Tongue at 7 Weeks.
**Figure S21.** *Lac*Z Histochemistry of Submandibular Salivary Gland at 7 Weeks.
**Figure S22.** *Lac*Z Histochemistry of Thymus at 7 Weeks.
**Figure S23.** *Lac*Z Histochemistry of Urinary Bladder at 7 Weeks.
**Figure S24.** *Lac*Z Histochemistry of Oviduct at 7 Weeks.
**Figure S25.** *Lac*Z Histochemistry of Testis at 7 Weeks.
**Figure S26.** *Lac*Z Histochemistry of Uterus at 7 Weeks.
**Figure S27.** *Lac*Z Histochemistry of Ovary at 7 Weeks.
**Figure S28.** *Lac*Z Histochemistry of Prostate at 7 Weeks (Part 1).
**Figure S29.** *Lac*Z Histochemistry of Prostate at 7 Weeks (Part 2).
**Figure S30.** Histology of Mouse Maxillary First Molars at PN5.
**Figure S31.** Histology of Mouse Maxillary First Molars at PN11.
**Figure S32.** Histology of *Fam83h* null Mandibular Incisor at 7 weeks.
**Figure S33.** SDS‐PAGE and western Blots of enamel proteins.
**Figure S34.** Proteins that immunoprecipitated with FAM83H.
**Figure S35.** Protein sequence alignment of FAM83H orthologs from eight vertebrates.
**Figure S36.** FAM83H phosphorylation by CK1 in vitro.
**Figure S37.** *FAM83H* Exon 5 PCR primers and reaction conditions.
