# Supplementary material for: Fam83h null mice support a neomorphic mechanism for human ADHCAI
Source: Mol Genet Genomic Med. 2015 Sep 21;4(1):46–67. doi: 10.1002/mgg3.178 (PMC4707031; doi:10.1002/mgg3.178)
Supplement: Supplementary file 1 — Figure S1. FAM83H disease‐causing mutations. Figure S2. Family 1. Unaffected Brother (II:2). Figure S3. Family 1. Unaffected Father (I:1). Figure S4. Family 1. Unaffected Mother (I:2). Figure S5. Family 2 Chromatograms. Figure S6. Fam83h‐Knockin Construct and Genotyping Strategy. Figure S7. Mouse Fam83h Wild‐Type and NLS‐lacZ‐Knockin Sequences. Figure S8. Wild‐type and Fam83h null mice at 7 weeks. Figure S9. bSEM Images of Manibular Incisor Cross Sections at 7 weeks (lower magnification). Figure S10. bSEM Images of Manibular Incisor Cross Sections at 7 weeks (higher magnification). Figure S11. bSEM Images of Manibular Incisor Cross Sections at 7 weeks (highest magnification). Figure S12. LacZ Histochemistry of Developing PN5 Fam83h +/− Mouse Teeth. Figure S13. LacZ Histochemistry of Developing PN5 Fam83h null Mouse Teeth. Figure S14. LacZ Histochemistry of Developing PN6 Fam83h null Mouse Teeth. Figure S15. LacZ Histochemistry of Developing PN9 Fam83h null Mouse Teeth. Figure S16. LacZ Histochemistry of Developing PN11 Fam83h null Mouse Teeth. Figure S17. LacZ Histochemistry of 7‐Week Fam83h +/− Mandibular Incisors. Figure S18. LacZ Histochemistry of D28 Fam83h +/− Dental Papilla. Figure S19. Histology and LacZ Histochemistry of PN5 Perioral Skin. [file MGG3-4-046-s001.pdf]

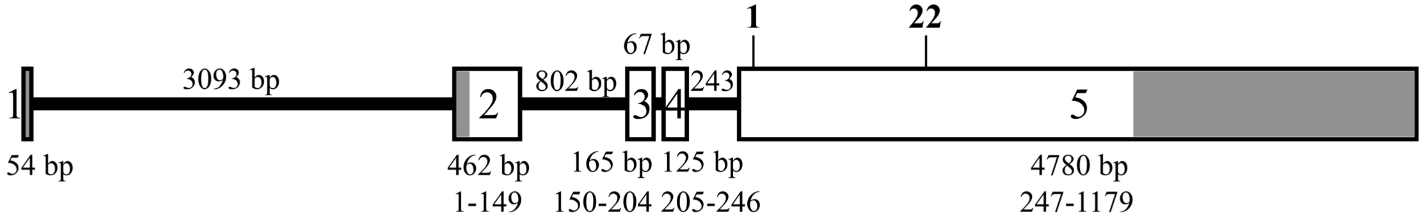

#	Gene	cDNA	Protein	References
1	g.10144C>A	c.860C>A	p.S287*	(1)
2	g.10175T>A	c.891T>A	p.Y297*	(2)
3	g.10190T>A	c.906T>A	p.Y302*	(3, 4)
4	g.10207dupT	c.923dupT	p.V309Rfs*16	(4)
5	g.10207_10208delTC	c.923_924delTC	p.L308Rfs*16	(1)
6	g.10257C>T	c.973C>T	p.R325*	(4, 5)
7	g.10476C>T	c.1192C>T	p.Q398*	(5-8)
8	g.10527G>T	c.1243G>T	p.E415*	(2)
9	g.10573C>A	c.1289C>A	p.S430*	(8)
10	g.10614C>T	c.1330C>T	p.Q444*	(6, 7)
11	g.10638C>T	c.1354C>T	p.Q452*	(3, 4, 9, 10) Family 3
12	g.10650C>T	c.1366C>T	p.Q456*	(6)
13	g.10653C>T	c.1369C>T	p.Q457*	Family 1
14	g.10658C>A	c.1374C>A	p.Y458*	(11)
15	g.10663G>A	c.1379G>A	p.W460*	(1)
16	g.10664G>A	c.1380G>A	p.W460*	(2)
17	g.10692C>T	c.1408C>T	p.Q470*	(1)
18	g.11156_11157delCC	c.1872_1873delCC	p.L625Afs*79	(1)
19	g.11199A>T	c.1915A>T	p.K639*	Family 2
20	g.11277C>T	c.1993C>T	p.Q665*	(12)
21	g.11313C>T	c.2029C>T	p.Q677*	(2, 4, 8, 10, 12)
22	g.11364G>T	c.2080G>T	p.E694*	(1)

Fig. S1. *FAM83H* disease-causing mutations. *FAM83H* gene structure: numbered boxes indicate exons; introns are lines connecting the exons. The numbers above each intron indicate the length of the intron in basepairs (bp). The numbers below each exon show the length of the exon in bp and below that the range of amino acids encoded by it. Shaded exon regions are non-coding. The 20 reported *FAM83H* nonsense or frameshift mutations are located between the sites marked 1 and 20 in bold. The gene numbers start from the first nucleotide of the National Center for Biotechnology Information (NCBI) *FAM83H* genomic reference sequence NG_016652.1. The cDNA numbers start from the translation initiation site of *FAM83H* cDNA reference sequence NM_198488.3.

References (Figure S1)

1 Wright, J.T., Frazier-Bowers, S., Simmons, D., Alexander, K., Crawford, P., Han, S.T., Hart, P.S. and Hart, T.C. (2009) Phenotypic variation in FAM83H-associated amelogenesis imperfecta. *J Dent Res.*, **88**, 356-360.

2 Lee, S.K., Hu, J.C.-C., Bartlett, J.D., Lee, K.E., Lin, B.P.-J., Simmer, J.P. and Kim, J.W. (2008) Mutational spectrum of FAM83H: The C-terminal portion is required for tooth enamel calcification. *Hum Mutat*, **29**, E95-E99.

3 Haubek, D., Gjorup, H., Jensen, L.G., Juncker, I., Nyegaard, M., Borglum, A.D., Poulsen, S. and Hertz, J.M. (2011) Limited phenotypic variation of hypocalcified amelogenesis imperfecta in a Danish five-generation family with a novel FAM83H nonsense mutation. *Int J Paediatr Dent*, **21**, 407-412.

4 Song, Y.L., Wang, C.N., Zhang, C.Z., Yang, K. and Bian, Z. (2012) Molecular characterization of amelogenesis imperfecta in Chinese patients. *Cells Tissues Organs.*, **196**, 271-279.

5 Kim, J.W., Lee, S.K., Lee, Z.H., Park, J.C., Lee, K.E., Lee, M.H., Park, J.T., Seo, B.M., Hu, J.C. and Simmer, J.P. (2008) FAM83H mutations in families with autosomal-dominant hypocalcified amelogenesis imperfecta. *Am J Hum Genet*, **82**, 489-494.

6 Hart, P.S., Becerik, S., Cogulu, D., Emingil, G., Ozdemir-Ozenen, D., Han, S.T., Sulima, P.P., Firatli, E. and Hart, T.C. (2009) Novel FAM83H mutations in Turkish families with autosomal dominant hypocalcified amelogenesis imperfecta. *Clin Genet.*, **75**, 401-404.

7 Ding, Y., Estrella, M.R., Hu, Y.Y., Chan, H.L., Zhang, H.D., Kim, J.W., Simmer, J.P. and Hu, J.C. (2009) Fam83h is associated with intracellular vesicles and ADHCAI. *J Dent Res.*, **88**, 991-996.

8 Wright, J.T., Torain, M., Long, K., Seow, K., Crawford, P., Aldred, M.J., Hart, P.S. and Hart, T.C. (2011) Amelogenesis imperfecta: genotype-phenotype studies in 71 families. *Cells Tissues Organs*, **194**, 279-283.

9 Hyun, H.K., Lee, S.K., Lee, K.E., Kang, H.Y., Kim, E.J., Choung, P.H. and Kim, J.W. (2009) Identification of a novel FAM83H mutation and microhardness of an affected molar in autosomal dominant hypocalcified amelogenesis imperfecta. *Int Endod J.*, **42**, 1039-1043.

10 Chan, H.C., Estrella, N.M., Milkovich, R.N., Kim, J.W., Simmer, J.P. and Hu, J.C. (2011) Target gene analyses of 39 amelogenesis imperfecta kindreds. *Eur J Oral Sci.*, **119**, 311-323.

11 El-Sayed, W., Shore, R.C., Parry, D.A., Inglehearn, C.F. and Mighell, A.J. (2010) Ultrastructural analyses of deciduous teeth affected by hypocalcified amelogenesis imperfecta from a family with a novel Y458X FAM83H nonsense mutation. *Cells Tissues Organs*, **191**, 235-239.

12 Lee, S.K., Lee, K.E., Jeong, T.S., Hwang, Y.H., Kim, S., Hu, J.C., Simmer, J.P. and Kim, J.W. (2011) FAM83H Mutations Cause ADHCAI and Alter Intracellular Protein Localization. *J Dent Res*, **89**, 1378-1382.

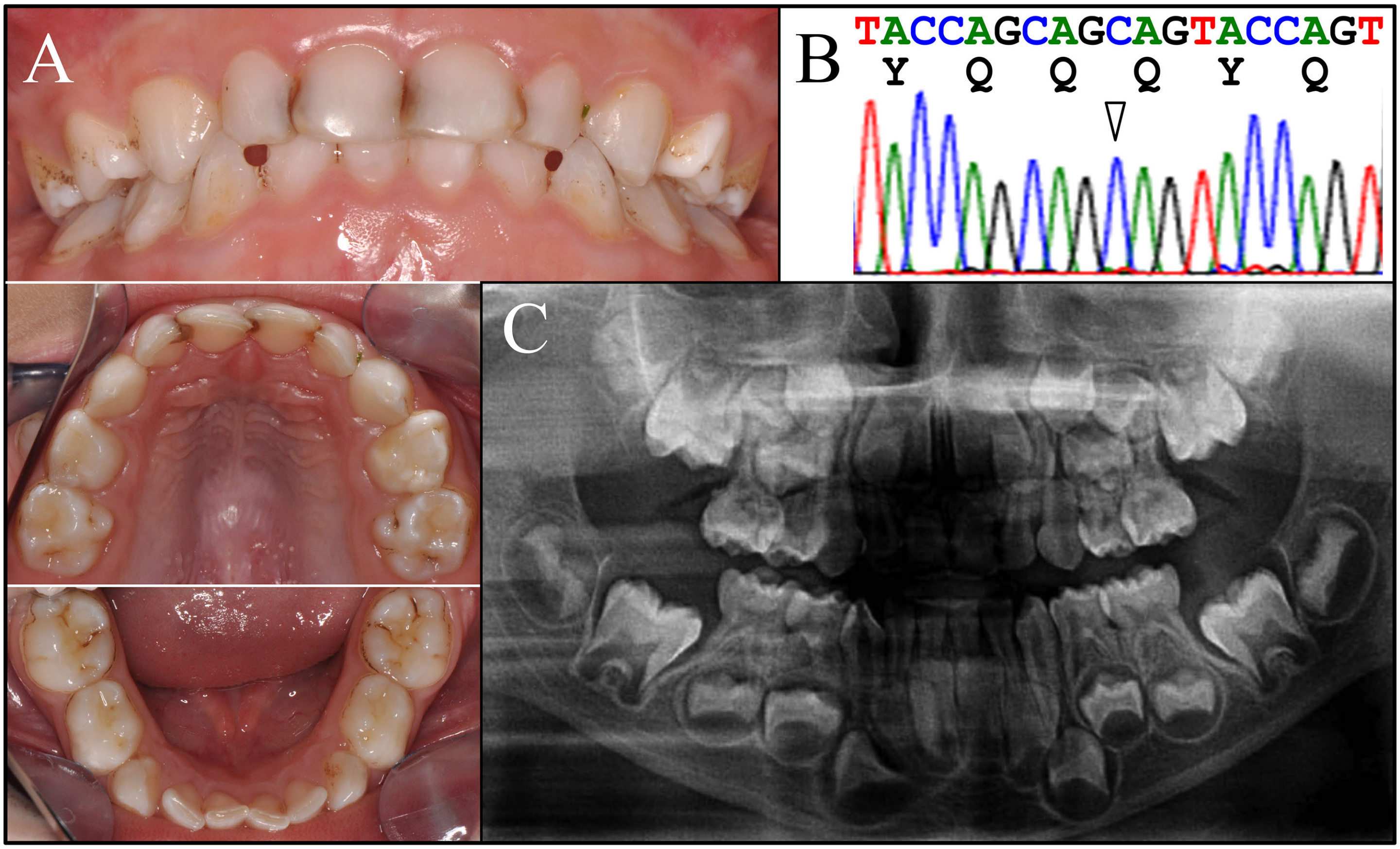

Fig. S2. Family 1. Unaffected Brother (II:2). ***A:*** Oral photographs. ***B:*** Chromatogram showing normal *FAM83H* sequence in both alleles at the site (c.1369C>T) mutated in the proband. *C:* Panorex.

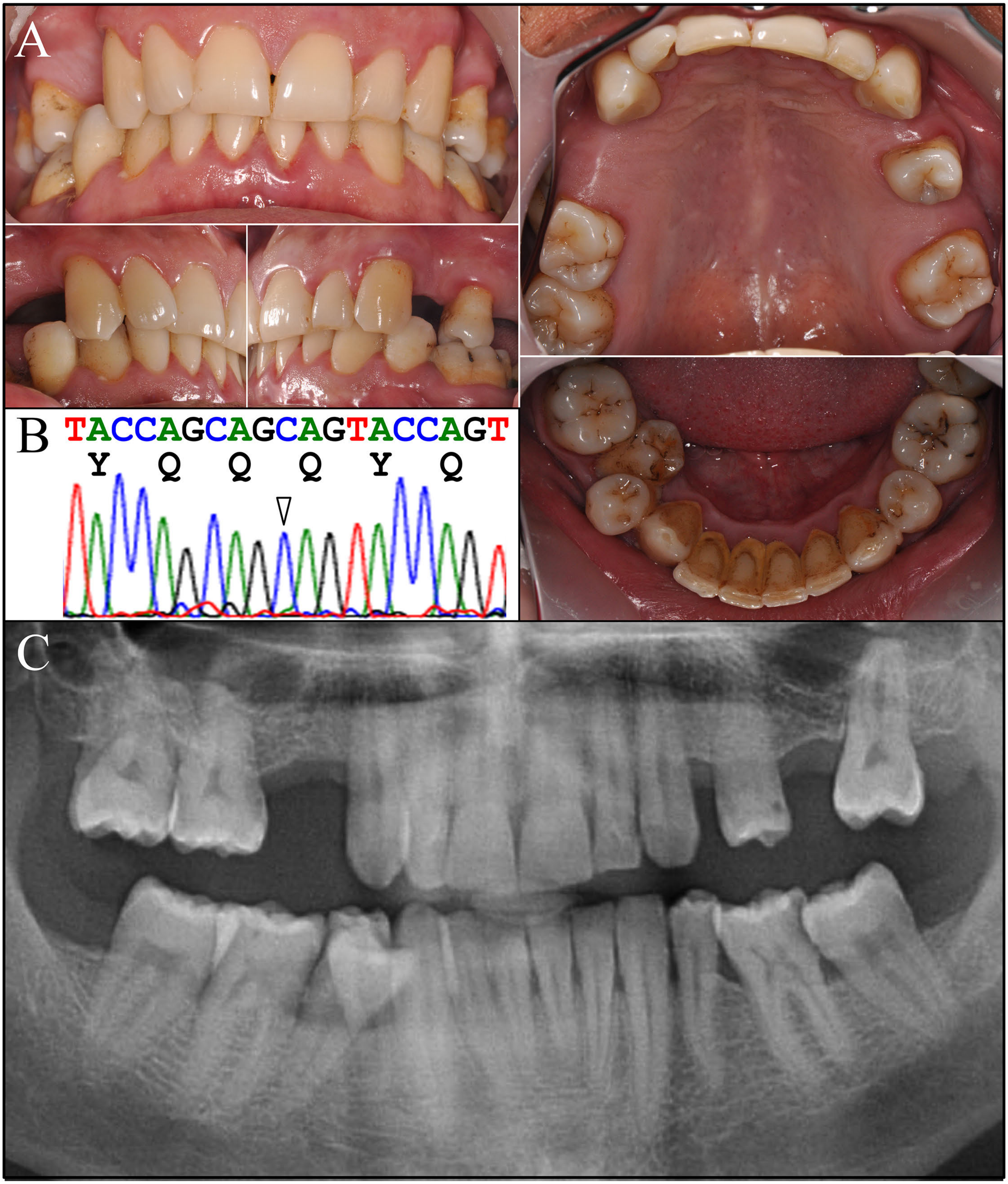

Fig. S3. Family 1. Unaffected Father (I:1). ***A:*** Oral photographs. ***B:*** Chromatogram showing normal *FAM83H* sequence in both alleles at the site (c.1369C>T) mutated in the proband. ***C:*** Panorex.

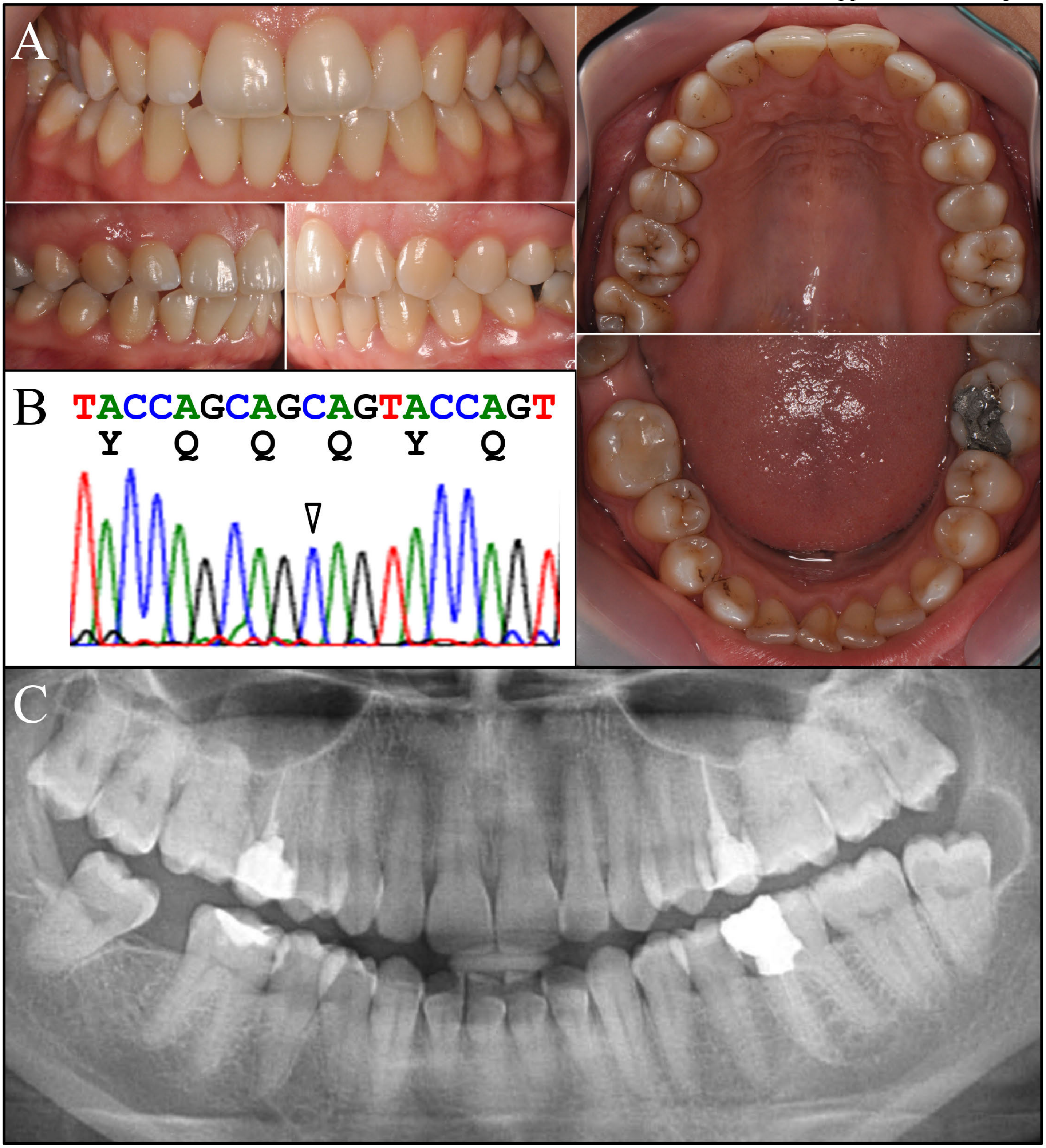

Fig. S4. Family 1. Unaffected Mother (I:2). ***A:*** Oral photographs. ***B:*** Chromatogram showing normal *FAM83H* sequence in both alleles at the site (c.1369C>T) mutated in the proband. ***C:*** Panorex.

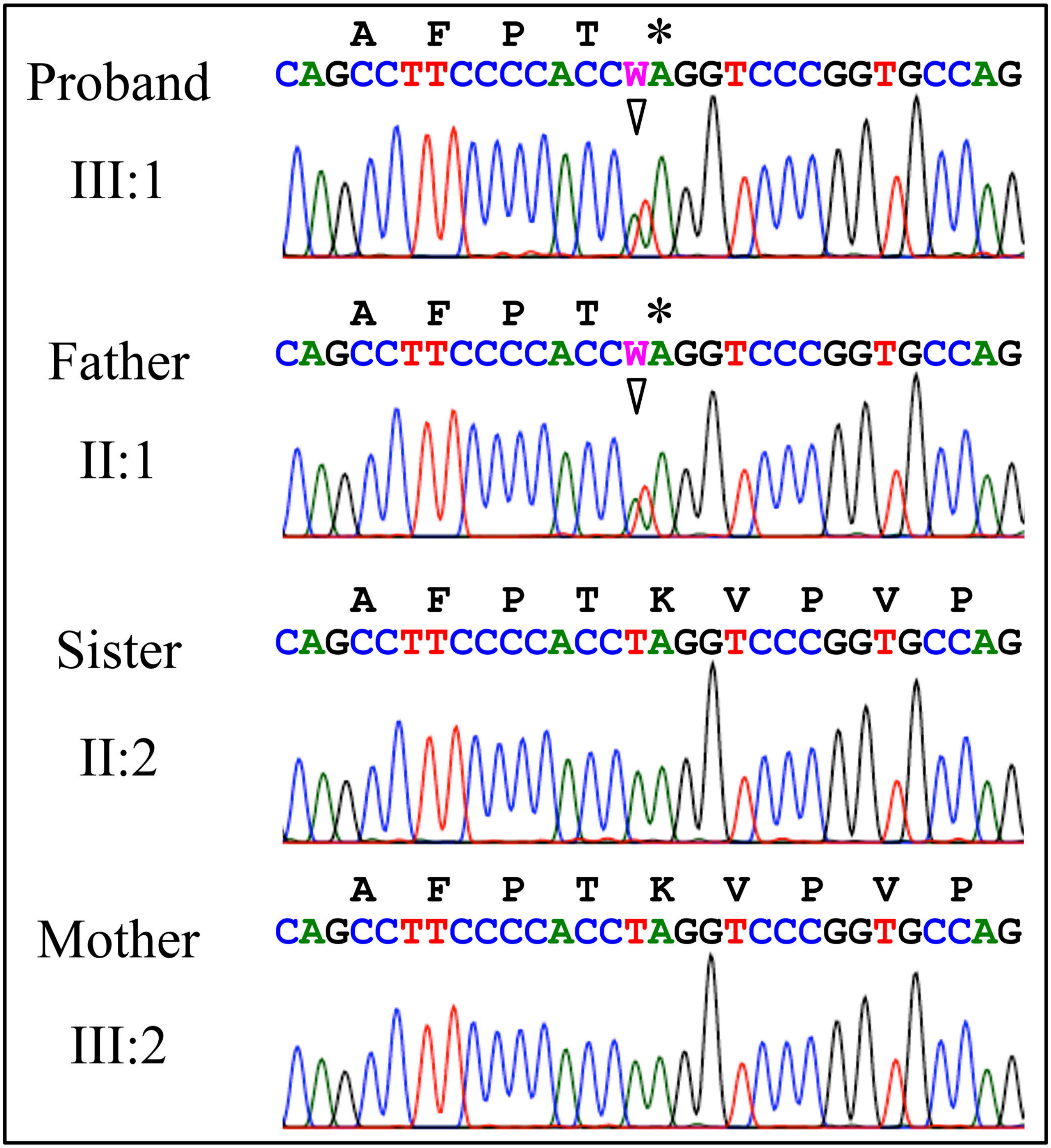

Fig. S5. Family 2 Chromatograms. The affected proband (III:1) and affected father (II:1) both showed the single allele *FAM83H* truncation mutation (g.11199A>T, c.1915A>T, p.Lys639*) that was absent from unaffected mother (II:2) and unaffected sister (III:2) and therefore segregated with the disease phenotype. W = A or T.

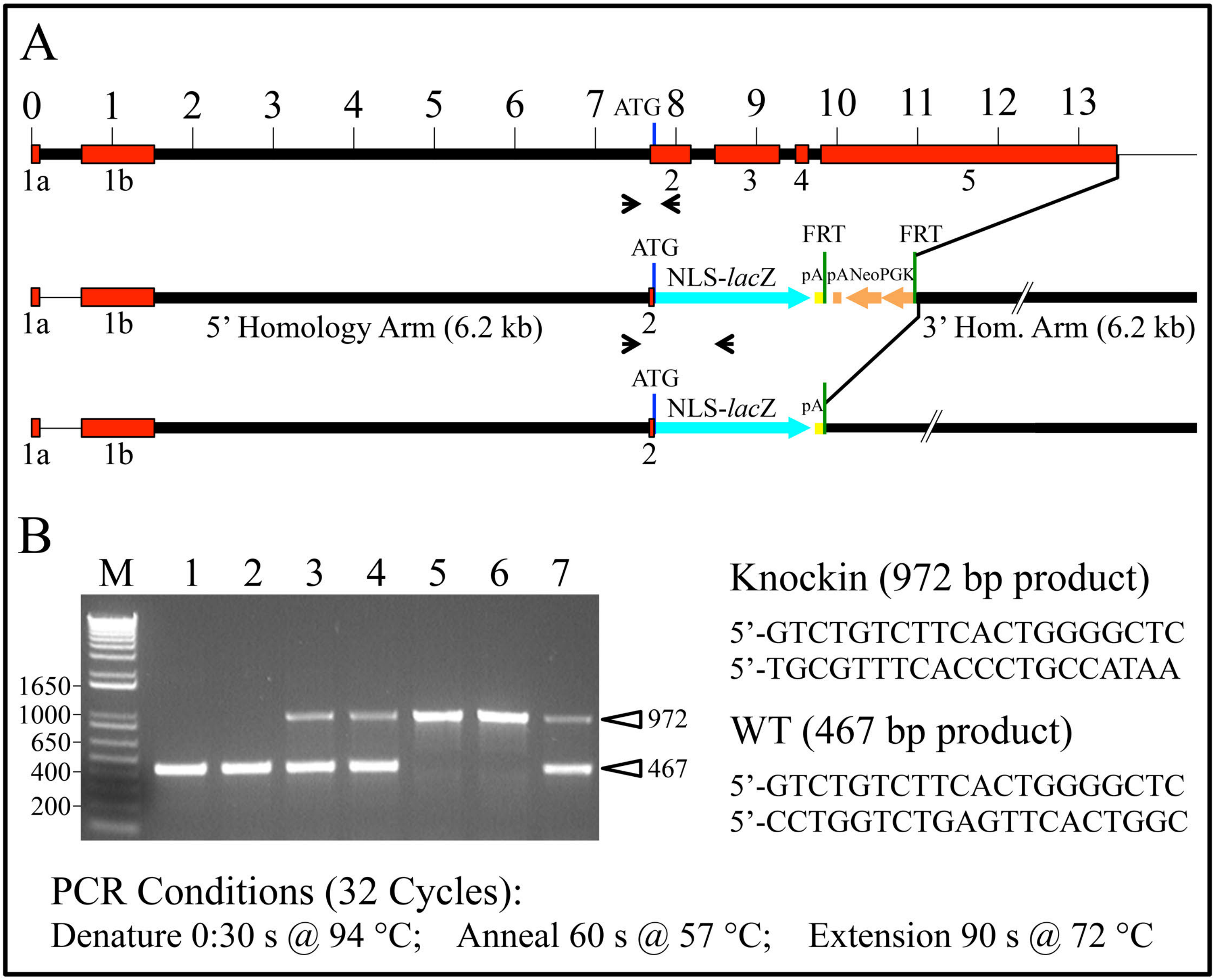

Fig. S6. *Fam83h* Knockin Construct and Genotyping Strategy. ***A:*** *FAM83H* gene structure (*top*): The numbered boxes (red) are exons. The numbers 1-13 indicate thousands of bp. Zero on the left marks the 5' end of the mouse *Fam83h* genomic reference sequence NG_016652.1. Exons 1a and 1b are expressed from alternative promoters and are not translated. Translation initiation begins in exon 2 (ATG). The thin line following exon 5 indicates downstream untranscribed sequence not included in the genomic reference sequence. The first *Fam83h* knockin structure (*middle*). The entire *Fam83h* coding region starting at the translation initiation codon and extending to the end of exon 5 was replaced by the mouse *lac*Z coding region that was modified by adding a nuclear localization signal (NLS) and two downstream polyadenylation signals (pA, yellow). The Neomycin (*Neo*) and selection Protein kinase C (*PKC*) selection genes and associated downstream sequences (pA, brown) were bracketed by flippase recognition target (FRT) sites. Arrowheads indicate the PCR primer annealing sites used for genotyping. Final *Fam83h* knockin structure (bottom). Mating with flippase (*FLP*) deleter mice resulted in site-directed recombination and germline deletion of the *Neo* and PKC selection marker genes. ***B:*** 1% agarose gel stained with ethidium bromide showing typical genotyping results. Lanes M, molecular weight marker; lanes 1-2, wild-type; lanes 3-4, 7 heterozygotes; lanes 5-6, homozygous knockin mice. The same 5' primer (that annealed near the 5' end of intron1) was mixed with two reverse primers (one specific for the wt, the other for the knockin) for genotyping. The next figure shows the exact sequences that were deleted and inserted.

A

Exon 1a (nucleotides 1-93) is non-coding.

ACCTGGAGCGACTCCTCGAAACAGGAGGCCTGGGAAGGAAGCCTCAGATGAGAGTTGGTCTTGGACCCTCAGCTCCACCCCTGGGACTAACAG

Intron 1a (nucleotides 94 to 7774) includes Exon 1b and Intron 1b.

gtgggccacgtgagggtcccttaggccttgctttaatttaaaggggggtgctggactcccaatcatggcagtagtcaggtcccacaccccatcccaggagccaagctagactc
ttctttatctagacttttgaaggctcagtgtcttcaccagcccacggtgtggcccaagtgcttcatgctgggcctagggtgggccttcatctctggcaaactcctgtctggc
agggttcgtccgggtaggcctggggctccagtggtcagctgggttgggatgacctatggccagtgagggtctgggcagttagggctggctggtgcatttgggatgcagctct
tggctctgtggccagtttgagtttcccgcgaccagagactgagggtggggtaaagggggggggcttggtgacacccaggcaggaccaggcaagtcctcaagcagcagtttag
ctactgagagccgcccccagtttctcagtctccctcccagtctggccagtgaagtctgtggcctgtaaagtctcgcaccctgtgtcagcg

Exon 1b (nucleotides 632 to 1540) is non-coding.

tgttagtcagcctcctcggctagggtgagccactgtgccgccaaagtctctataaactctctggtgtggcccagtgccacagagtgggacctgtggggcctataggcagagc
cagggccctgggctctgctaccggggtgcggagatcagccaaggggtagcacagccacttgggacagcggggagcagagggctgaacctggctgagagcaagcaacctcttg
tggcctctgggatgtcctctgagctccctaaccaggccagttgatgagccaggcccctggggagcagacatctggagaaagaggcactggattcagggccatgtctgggaaa
cgagggcgagctcgaaagccccgaggcaagagggtgggcagggcccagcgggcagctgggcagcagggccctgaggctgagcccccgccccccaacacaggggggccctcagg
accaggtgggggtggccccgacagagcacgtgaccagcactcagccggctcctcagggacactccagggccaccaggccagagcagctacaggcagcctggaggccagttca
ggacattgctttgggatccgagctggtgctggcccctgagcacatggagggctgtggagctggggcccccatgccagccctgggccctgaattgctgagactccacgaagtc
cagctgtgcctagcccaggagcagctgctgctggaggatcggaggcggcaggtccagctgcagatgcagctgtggcaggaggagcagctgtggctgcagcagctgcaggagg
agcagctgtggctgcagcagctgcaggaggagcaggcctgggtgcacatggaggggctgcagctggctgtggccctggagcagctccggagtgagggcctcgaggcactgca
aactcaaggccag

Intron 1b (nucleotides 1541 to 7774).

gtaaggcctagaggggtatggggcaggggctcgggcagtgacagtacagtggacactcaagcatcttataggtgtgtgaaggatgggaagccaggttggctgtagaacgtct
gtgggcctgtccaccaagaggggctagtgctcactacataggatgttgcctgggtgaaggacagcaagcctgcccagaccacccaatgcttggtaggcaaggtcattgaggg
agggaacttgcttggggatggaggtgtcaggggaccaggataagtagctacaggagtggtccagggcggtagggtgtgtgttggggggggagggccgggtgcacagctgatag
acaacatcagaaccaagaaacagtccctaccccaagcatcctggacaacttctccgtctggtccacccgccctgccccgtgtttactgcagcttggtgcccagggcttgaga
tcacagaggcagaacgcaatgctggtacccattatcctcattccaggaggaagactggcctcagacgcagctggggctattttgggcaactaggatctcacctcagcgtagt
gtggagtcaccactgcctgtcagccccagggaatctaagatgtggggctgcgtgtgggtctggggtgggtcccctgacagcgggtacagactccaggcaagctcggtgcaag
agacagtctgggaagatgttcacagctgtggcagaatgaatgggactcagagcagaggggcgataaaggctgcaggagggtaggcacaggggcgagggagaccttttctga
tgctctgcaggagccagcccaggctggctgtaacccttcatccttcctggaggtgcttacagacaaacagtctgaaggggaaggcaggactgtgggatggtgaatagacaac
cacttagcatgaagattcccaactcagaatcaaggcctgctttttctcaatattgctttgttgctgttgttgtcgtttccagacagggtttctctgtgtaaccctggctgtc
ctggaactcactctgtagaccaggctggcctcagactcagagatccgcctgcctctgcctcctgagtgctggtggcagtgtgcgtcaccaacgacctgcttcgatattttt
ttaagctatctgcccctccaatctcctccttaccctaacactgtaccctcctgagcactgtacccacaccccttctccagctctagaagttcggggtgcagtggacatctga
gctttgagtcacaaatctatcattccacgcccaactgaaagatctcttggaaaccacttggagagaaagggtttctgtgtgcctagtggtctcgtggtcagtgttggggtag
tgccctggaggtcagaggggcactcaccagcagtagctcgaagcttggacccagggaggagcgcctgccccttccctgatgcagctttgggcccccccttcttagtgctca
ttctcctggggggtgggaaggaaaagtccagggatggtacattcaggagggcagcgaacaaggcagtgggaatactgccccggccctggatgaacatgcagcaggagcataga
cctccagcatgtcccccagccagcttcttggtccacctgcccgcccctggtggtctaaggatgtgcccagtagctcgaggaaggatacctagcaagaggatgtgtcccttta
tgctggtaccccccgtcctctacaattctggatctgcctgtccttctgtgttctaacaccatcctctaaatcgctgggctagaagctcaacagcctgagctctgcggacttgc
tttcaactactgccaacgtgggtctgctgaacacagcccctccccacccgcaaaccctcttccttcttggccttgggaatctctgctgtctggttctggaagcaatgccctt
ctccctagtatacaaggcaggcccccattcactcctcttggaagaactttccgctcctgttttagtagggtcttccagtgccctagaacccctcacaggccaatgaatag
gaaagcgggtagtgcaatcgctgactccatacacttatgtacccactaggctggggccgcagggctccagagtgcatttggggatggagttaatgaaatgttatcctaaaag
taggaccagaaagtgactgaaagtaagctgtcttacggacaatgttcaggcaggtggcagacactgagaatctaggacaggagaagtggtaggtaggaagctggcttggaca
tgtgactgagttcagggcacggcatgcgtagaggagtgggttggagggagggctgggtagattagggctctaggaggaactaagtccttaggtgtacgggtgatgtgttggg
gtcgatacaaccatgaggttccctgtctgtagaggatccagtacttgcagcatcttgggtgcagcctcttttaatgtgcatgtattaagacgggacctttgtgtcttcagtt
ggtttgcacaggaaaggatcagtgggtgccaggaccgacactgttggagcagtttagagagtgatcctcatgtgagatggggacttgggggtacgattggtttgcgggatt
ctgtgtggcagaggggcttctgctggcggatcactccagattcccttgtgcctgaggggtcccatgggtacgggtgagtgcgcaggaacctttatggttgggcaaatgagtg
gggaggtccgtggagttcctatgctgtcggtctcatgcgcgaagatgagcctgcggggagtggactggggaaactagaggtgtgtctgcgaacggtagggcggtcacttgag
tgagatgggtacccaaacaccccccccccccccccccgtctgagacctcacctgcctcacctgcctctcgcaacacagaggccagaagctccgcccctcgcgtcgagctggc
cagtttcctagcgactaagccggaacttccgagttaccccaacttccggggcgagggtcaggggtcggcagtaggggctttgagcgatcggttttcaaataggttcaagggg
cttcagtgtccggaaagacgaggctctagaaatccggagcggcgctggccagggtccggagcgcagctctggggctccaccagagcgctcaggggtcggggttggggtgtgg
gctgggggcgccagtgaagccacactgggaacccgggatcgcggccggggtccggggcgcggagggcgcaggaatgcgggcgcgtcggggtggggccgccccgcccaggtgc
tgcccggacaggcaggaggcagagcgcgggcggggcgggggcgggcgcgtgaatcaggccgaacgggcgggcgggccgcggcggggagtggctgtcggacggacggaccgc
gaggccgctgggcggcggtgggctcctgctgcccctgtgccgaaaccccgctcacctggccaggtagggctcccgccccggagcgggccgggcacagcacggcggggcgagg
cacacgggacgggaggctcgggcctccgcgcggtgccacctgggtctggtctctggacctgggccgggcggtgcagtgagcgcgtgctttgggactgggtgggagacaggga
gactgcgctccggggcactgggtgagggattttccagggtcacgctggcaagtaggagactgaacagtgcgattcgctctggcggcctaggtattgtcaggacatgacctgt
acctttcccggagcctctccccgagccgccatgaaccggggtgtctagggctgggtccttgtgtggaaggcagggaaaactggactctcctaactcctgatctcatatccct
ggactggatcggcagataagatacctggcctgggtagccgctaacttcctggggtacagtaacacctttgggccttgcagtgcctaacctgaaagctgtataattcatcgac
ttccggtcttcctaggttcttcttgtcccccacaaccccaatccttcttgatcacaatcatcctgtcatcctctttgtgtagtagcctaaggtcctcaaccatggatccccc
ttccagacccaggaacactactacctcctgccagacaggagaggaaagggtcattttcttgccctacttgtggtgaagggcctggggctagcttcaggtaggaggtcaggaa
caaaaaattcctgctgagtcactggtttgggtgggataggctccttccttaagcagggccccacttggataggcagatgtcagtgagcaggctgttggattcatcgctgata
ggagtggtcaccgccccgtctatcgtcaaggggcgatgtgtaccagtgaatgattgtaagggcatttaaggtcgtgtgtgacttctgtgaggtcaggtaccacagtggccgc
tcaggactaaggccacttcccaacccatgtgtctctgagggttctcaccactggctagacagtatcagttctcagtcttatacaaggaagtgtcccctcagcgtgagcatgc
taggatccccaggtagtgtgggaatgctgcctaggagagctggctccagtctgagacctgtccaagcagacaggcccgcatcagaccccaggtgctctgcttccatctcccc
agaacagtgtgccacgcctcacattgcagacaggaaggaaaagcaacagccttaggtcagggcaatgccccagagggaagtcggagcactcttcttagggccagtagacata
ggagtgtccccacctccaatctctgtcacacttggacttgctaatgtgttacgccatgcccctaggggtggcagacactgtagctccctgggtcatacacagcagagtcccg
ctggacccccaacgtcattgaacaagtatcccctaataccagagctgccatcctgtcagcatcgctggcccactcctcaaggcactcctcctcctgggcagaaatacttccc
tagtttggactatcatccaggaacctgtgtgctaggcagggcaagagtcccaagccatttcaaacctgtggagcagtggggaatggtatgagcctgccctaaggcaacttcc
aggccagaggccaccaggtttgcaggcgtttggttgtcacttctaatgccttcttcaaccacaagcttctgctactcgcactcatggcccaggacctcctgatgtggacat
gaacggtttctcgggcacatacttgccagtgaactcagaccaggctggacctgggctagcccgtgacatctggcttccaaggcacaggagtcatgctctctcatgagcgcac
atgcacgcacgtgtgtgtgcacacacacacacacaccagccactagagaacagtgatcctgaggcacctgtgatgggggacgctgacagagaacttgggacgaggcttgtag
caaagtcttccttcattcttagtgtattagggagagggtggagatccggggccttgggtattggcaatggctctgttgccatcaggatgggtgagtgagtctgggagggtat
aaagaagagaccctacagggaactgtgagttcccaaggaactgtggcccagggaggttttagctgaggtgagacggaagcaagggtgaagattttcagagaggtcgaaggtt

gaggagtcaggacagggagatcagtgaagggactgtgggcagatgcaggtgtggactgaggaaagaggtgtgggtggggcttaaggccatcatccctggatgagtcaatct
gcactccactgtgctcaccttgggccaggtatgatgtcttgccacccctcccagctctgtgcagtgaaggacacaggctagagcaaaggggggggggggaaggaataatc
tctttaactggcctgcatttgtatcttaaccaaaacttatctttacaggaaaagggaaccacagagtaaatgggccgtgaatgtattgactctgaagtaaggggaggtggga
cctcttacctgtattggggtggggacaaacattggcagtcactgagactgatagaaacaaggtgtcagccccaggaaggggattaagggctgagtaccactggcccatgtct
taccagctaggcacatgctacggcctgtggtgctgcttcagagtctgtcttcactggggctcagaaaaactcccgggcataagctaaacccaagatccagcagccacatcag
ccgtgtgtgtccacttgtggcatgaggtaagtgggtcctactgcccctagttaatgcattttcctgcccctccag

Exon 2 (nucleotides 7775 to 8236).

gcccctggccccaac

atggcccgtcgctcccagagcagctcgcaggggacaacccactggcacctgggtacctgccacct
M A R R S Q S S S Q G D N P L A P G Y L P P

cactacaaagaatattaccgcctagcggtggatgcattgactgagggtggccagaagcctacaac
H Y K E Y Y R L A V D A L T E G G P E A Y N
cgcttcttggcatctgagggggcacctgacttcctgtgccctgaggaactggaacacgtgagccgc
R F L A S E G A P D F L C P E E L E H V S R
cacctgcagcccccacagtatgtggcccgggaaccccctgaaggcaccccatctgatgtggacatg
H L Q P P Q Y V A R E P P E G T P S D V D M
gacgggtcctcaggcacctactggccagtgaactcagaccaggctgtgcctgagctggacctgggc
D G S S G T Y W P V N S D Q A V P E L D L G
tggcccctcacatttggcttccaaggcacagaggtcaccacactggtacagccgccgccacctgac
W P L T F G F Q G T E V T T L V Q P P P P D
agccccagcatcaaagatgaagctcggaggatgatccgctctgcccagcag
S P S I K D E A R R M I R S A Q Q

Intron 2 (nucleotides 8237 to 9037)

gtgcattctcacgggcttgaaggatggtgaaaggcagcccaagccgggttttgaagcatgaataggagggtgtgggagatataaaagcattcctggtgcttaggatgccagg
gctggccagccagacctcggcagtagggttgccctgtagagtctgctcattatcacctgggtcttgggtagcgggttctgtagacattccagtatctaggatcatggcctgt
acccagaaactcagggtcttgcccagctgagagcaccccaccccaggcagctggtaaactgaaaaaccaagtgtttaagccaggaagactggctcatgcctgtaatcctagc
gcccagggaggtggaggccagcctgggctacacggtaacagaccagtcctcattaagaaacaggaacactcctacaaaacccaggaatattaaagagaggtagcaaggccag
atggtaaaggcctagcttctgcccctgttccaggaagttgacaaaccgcaccttgaccacttggggtctgaacacacggagctgcagataatcccccctctgaatgaagcacc
ctaaaagtatagcccctgcttctgcttcaagctccctcaagggctaccaagtgtcaattttgtgtgtgtgtgtgagaccttctgctgggtcccttcttcccccacctccca
attcatgaccagtctctcctggggcaggctaatggtcatttttatcgtgcttgtgggtgaaaatgtggccagagatgggcaggatgaccaggcccttccctccccgctgacc
tgatacccacctcccag

Exon 3 (nucleotides 9038 to 9202)

gtggtagctgtggtgatggacatgtttaccgacgtggatctgctcagtgaggtgctagaggccgct
V V A V V M D M F T D V D L L S E V L E A A
gcaaggcgagtcccggtctacattctgctggatgaaatgaacgcccagcacttcctagacatggcc
A R R V P V Y I L L D E M N A Q H F L D M A
gacaagtgtcgcgtcaacctgcatcatgtggac
D K C R V N L H H V D

Intron 3 (nucleotides 9203 to 9271)

gtgagtgaccaagccaggggagtggagggaaaatatctttggtgaagacctgactcccctctcttccag

Exon 4 (nucleotide 9272 to 9396)

ttcctgcgtgtgcgcacagtggcaggtcctacttactactgccgcactgggaagtctttcaagggc
F L R V R T V A G P T Y Y C R T G K S F K G
catctaaaggagaagttcttgcttgtggactgtgccgtagtgatgagcggcagttatag
H L K E K F L L V D C A V V M S G S Y S

Intron 4 (nucleotide 9397 to 9587)

gtatgcccagtgggttgcccttagccctggcccttcccacctagtcctccgtggtcccacttccttcccataaagctgtccttgccccagcccaccagcccaacccctttca
gatccctgattatccacctagtgcctttagcctctgtggccctatttcctctccctgactcctgccatcctgtccacag

Exon 5 (nucleotide 9588 to 13245)

```
cttcatgtggtccttcgagaaaatccaccgcagcctggctcatgtgttccagggagagttggtctccagctt
  F  M  W  S  F  E  K  I  H  R  S  L  A  H  V  F  Q  G  E  L  V  S  S  F
cgacgaagagttccgcatcctcttcgcacagtcggagccactggtgccctcagccggggcgctagcccgcat
  D  E  E  F  R  I  L  F  A  Q  S  E  P  L  V  P  S  A  G  A  L  A  R  M
ggatgcctatgcgctagctccatactctggggctggcccctggtgggcgtccccggggttggagcaccaac
  D  A  Y  A  L  A  P  Y  S  G  A  G  P  L  V  G  V  P  G  V  G  A  P  T
acctttttctttccctaaacgggcgcacctcctattcccaccgcccagggaagaaggcctgggcttcccctc
  P  F  S  F  P  K  R  A  H  L  L  F  P  P  P  R  E  E  G  L  G  F  P  S
tttcctagaccctgaccgccacttcctgtcggctttccgccgagaggagctgcagagaatgcctggggtgc
  F  L  D  P  D  R  H  F  L  S  A  F  R  R  E  E  L  Q  R  M  P  G  G  A
tttggagcctcacacagggctccggccactggcgcgcccaactgaggctgggccgttcggagagctcgcggg
  L  E  P  H  T  G  L  R  P  L  A  R  P  T  E  A  G  P  F  G  E  L  A  G
cccccggggcttcttccagtcaaggcacctggaaatggatgccttcaagcggcatagctacgcaacacccga
  P  R  G  F  F  Q  S  R  H  L  E  M  D  A  F  K  R  H  S  Y  A  T  P  D
tggagctggagcagtggagaactttgcagcggcacggcaggtgtcacgacaaacattcctcagtcacggtga
  G  A  G  A  V  E  N  F  A  A  A  R  Q  V  S  R  Q  T  F  L  S  H  G  D
tgacttccgtttccagaccagccacttccaacggaccagctctatcagcagcattaccagtgggacccaca
  D  F  R  F  Q  T  S  H  F  Q  R  D  Q  L  Y  Q  Q  H  Y  Q  W  D  P  Q
gtttgctcctgcgcgcccacagggcctcttcgagaagcttcgtgcaggccgacctggctttgcggaccctga
  F  A  P  A  R  P  Q  G  L  F  E  K  L  R  A  G  R  P  G  F  A  D  P  D
tgactttgccttaggtgctggtcaccgcttcccagaactcggtgctgatgtgcaccaacggctggaatacgt
  D  F  A  L  G  A  G  H  R  F  P  E  L  G  A  D  V  H  Q  R  L  E  Y  V
gccatccagcgcatctcgggaggtacgccacggctcggatccggcctttggacccagcccccgtggtctaga
  P  S  S  A  S  R  E  V  R  H  G  S  D  P  A  F  G  P  S  P  R  G  L  E
gcccagtggagcctcgcgtcccaatctgggccagcgttttccatgccaagcaaccttgagacaaggcctgga
  P  S  G  A  S  R  P  N  L  G  Q  R  F  P  C  Q  A  T  L  R  Q  G  L  D
caccgcttcggaggcagaacctgagcgcaggggcggacccgagggccgggccgggctgcgtcactggcgcct
  T  A  S  E  A  E  P  E  R  R  G  G  P  E  G  R  A  G  L  R  H  W  R  L
tgcctcctacctgagcggctgccacggtgacggtggggaggagggtctaccaatggaggctgaggcttgtga
  A  S  Y  L  S  G  C  H  G  D  G  G  E  E  G  L  P  M  E  A  E  A  C  E
agacgaggtgctggctcccggaggccgggacctgctcccctccgccttccgcactcctgcagccttcccagc
  D  E  V  L  A  P  G  G  R  D  L  L  P  S  A  F  R  T  P  A  A  F  P  A
taagggaccaaagccgggctcaggaagcggtggtggtgacagctccgagcgagagggcccagaagagacaag
  K  G  P  K  P  G  S  G  S  G  G  G  D  S  S  E  R  E  G  P  E  E  T  S
cctggctaagcaggactccttccgctctcgcttgaacccgctcatccagcgcagctccaggttgcgctcatc
  L  A  K  Q  D  S  F  R  S  R  L  N  P  L  I  Q  R  S  S  R  L  R  S  S
actcatctttgcgtcccaggctgagggtgctgttgggaccgcagcagccaccactgaaaaagtacagctgat
  L  I  F  A  S  Q  A  E  G  A  V  G  T  A  A  A  T  T  E  K  V  Q  L  M
gcacaaagaacaaacagtcagtgaaactctgggtcccagcggagaggctgttcgttccagcgcctcggccaa
  H  K  E  Q  T  V  S  E  T  L  G  P  S  G  E  A  V  R  S  S  A  S  A  K
agtggcggagctcctggagaaatacaagggccctgcccgggaccctggcggtgcaggaggtgccgtcacttc
  V  A  E  L  L  E  K  Y  K  G  P  A  R  D  P  G  G  A  G  G  A  V  T  S
ctccagccacagcaaggctgtagtgtcccaggcctggcgggaggaggtggtagcaccaggaggagcgggaac
  S  S  H  S  K  A  V  V  S  Q  A  W  R  E  E  V  V  A  P  G  G  A  G  T
tgaacgccgcagccttgagagttgcttgcttgacctgcgcgattcctttgcccagcagttgcaccaggaggc
  E  R  R  S  L  E  S  C  L  L  D  L  R  D  S  F  A  Q  Q  L  H  Q  E  A
agagcgacacccaggagccgcttcgctcactgctgcgcaactgctcgacaccctgggcggcactgaccgcct
  E  R  H  P  G  A  A  S  L  T  A  A  Q  L  L  D  T  L  G  G  T  D  R  L
gccatcacgcttcctctccgcccagggccgctccttgtctccacaaggtcgagatagccctccgccagaagg
  P  S  R  F  L  S  A  Q  G  R  S  L  S  P  Q  G  R  D  S  P  P  P  E  G
```

```
gcttgggacacaccagctgccttattctgagccaaagggaaaccccaccccagcttaccctgagcgcaaggg
  L  G  T  H  Q  L  P  Y  S  E  P  K  G  N  P  T  P  A  Y  P  E  R  K  G
gagccctaccccagcttaccctgagcgcaaggggagccctaccccagcttaccctgagcgcaaggggagccc
  S  P  T  P  A  Y  P  E  R  K  G  S  P  T  P  A  Y  P  E  R  K  G  S  P
taccccagcttaccctgagcgcaaggggagtcctacccaagcctaccctgagcgcaaggggagccccacgtc
  T  P  A  Y  P  E  R  K  G  S  P  T  Q  A  Y  P  E  R  K  G  S  P  T  S
tggatttcccaatcggaggggcagcccaaccacaggattgatggagcagaagggaagtcccacttcaaccta
  G  F  P  N  R  R  G  S  P  T  T  G  L  M  E  Q  K  G  S  P  T  S  T  Y
cccagaccgcaggggcagtccggtgcccccagtgcctgagcgcaggggtagtccagtacccccctgtgcccga
  P  D  R  R  G  S  P  V  P  P  V  P  E  R  R  G  S  P  V  P  P  V  P  E
gcgcagaggcagtctcactttcgctggggagtcttcgaagactgggcctacagaggaggtgtctagtggccc
  R  R  G  S  L  T  F  A  G  E  S  S  K  T  G  P  T  E  E  V  S  S  G  P
catggaagtcctgcgaaagggttctctccgcctcaggcagctgctgagccccaagaatgagaggcgtgggga
  M  E  V  L  R  K  G  S  L  R  L  R  Q  L  L  S  P  K  N  E  R  R  G  E
ggatgagggcagcttcccaactccgcaggaaaatgggcagcccgagagcccccggcggccctcgctgagtcg
  D  E  G  S  F  P  T  P  Q  E  N  G  Q  P  E  S  P  R  R  P  S  L  S  R
gggtgacagcacagaggctgctgcagaggagagaggctcgagggtccgcctagcttcagctacagccaatgc
  G  D  S  T  E  A  A  A  E  E  R  G  S  R  V  R  L  A  S  A  T  A  N  A
tctgtacagcagcaatctgcgagatgacactaaggccattctggagcaaattagtgcccacggccagaagca
  L  Y  S  S  N  L  R  D  D  T  K  A  I  L  E  Q  I  S  A  H  G  Q  K  H
ccgcggggtccctgctccaggtccagcccacagcagtcctgacgtaggtcgtccaacaactgcaggagactt
  R  G  V  P  A  P  G  P  A  H  S  S  P  D  V  G  R  P  T  T  A  G  D  L
ggccccagacatgtccgacaaggacaaatgttcagctatcttccgctcagacagcctagggacacaaggccg
  A  P  D  M  S  D  K  D  K  C  S  A  I  F  R  S  D  S  L  G  T  Q  G  R
gctcagccgcaccctgcctggcagtgcagaggagcgagaccggctccttcgccgcatggagagcatgcgcaa
  L  S  R  T  L  P  G  S  A  E  E  R  D  R  L  L  R  R  M  E  S  M  R  K
agagaagcgtgtctacagtcgcttcgaagtcttctgcaaaaaggacgaagctggcagtagtggggcaggaga
  E  K  R  V  Y  S  R  F  E  V  F  C  K  K  D  E  A  G  S  S  G  A  G  D
caacttggcagatgaggacaccagggacagtaaaatgggcaaatttgtccccaagatcctgggcacattcaa
  N  L  A  D  E  D  T  R  D  S  K  M  G  K  F  V  P  K  I  L  G  T  F  K
aagcaaaaaatgatctcctggtctcggaggccaggactctgcatcactgccatactgaacctacaataccca
  S  K  K  *
```

```
tctggagtggtggctgtcaggttagtgtcaaagcagtttgggacctagctgacaaccacctgaactgagctccacttgaattcgcctcgcagccgcactgcctctcac
tcttggcttttccatgaggggcttagcccctccacctggtgcctttcctcatcacctacccccaccccagcctccaccatctcctgggctcaggtctactttgggtca
ttatttcacctcagggatccatctctctgccttatgctccagtttttcaagaactctatcactttgtgcctcagttccctcttaaggccttttatctcagggcttttt
ctcagggtttccatctcatggctcctggtcctctccctctggtgacagcctcctcccatcctctcatctctatctctctcgggaacacaccttgcctgctcccactct
gactcctcactgcctcatagcactttcttggcatctgccctcttactgtgtggatggcagagttggcctcgggcagtttaagggaagagagggatgctggctaggaca
gactgttggaaagtaattgctaaacacctagggctccccaccctccccgcctcaggccatgcctgccctctccagcaagtttctgggtagcacttgaagaggagccaa
gattggggagcactgggctttgcagtacaataaaggaatcgggacagacgttctgtga
```

3’ Untranscribed sequence (nucleotides 13246 to 14337; not in gene reference sequence). This sequence and the “a” that precedes it follow the NLS-*lacZ* insert highlighted in cyan below.

```
tcccaagcctcagtaaattcctaagccaaagtctggacagaggcatgtctatagtgtgccctcagcaaaaggcagaaggtccctggttactttttcttaccactaaggatt
aattttgatctggaatgacataaacacatggagagcccccacacacacagagagagagagagagagagaagcactgagaaatatgtgtctgctatctactatttttccaaata
gtttatacctgtgtctatggaatcccaaggggcaaagacatgagaacaaagccactataagtatttctgactccagcagctggagaagggagggtactgggacttggacag
tgacccaatttgccctgtaacatatcactcaaaaatccccaagttcctggccaggtgcacacggtcccctgggaactaggtgagccggagtgcttcctagtcttaacatgct
cactgcttggctcgtcttagggcactcggtggtctggtgtcctgtgcgtcactgctcatgctcagacctgttgaactttgcaatggaaagtggaggctggcagtccttcctg
ggtccctctgagcccccctgactgtgcctttcctggagccctctgagcctgctggctgtgcccttcacacctttctgagaaggacatttcctgaagcacggtgccccgtgttc
tgacattaatgataaacacaacacacaaattccaacatagcccagtgtgggaggcagacgggagctggtctctacattctggggcagcctggaactgcaagttccagaccac
ccagggctacacattgagatcctatgcaaaaataaaaggcaaaaaaaaaaaaaaaaaaagccagcatgtacataaataggtgtactatccaacgtgggtcagtctttatccc
tgtgtggccttcctagtttccttttggaaaaccatgagtcgtgtgcctctgtgcccatacaaacacctcccccatacctaccaaaccccatacaaacccctggctctttgg
gccatccacactcatctgggtctccagtctccaaagccgaaagacccttctcaaccattatctttggagcctgaactcttcctg
```

B

Area highlighted in yellow was replaced with the following NLS-*lacZ* sequence:

ATGGCTCCCAAGAAGAAGAGGAAGGTGATGGAAGATCCCGTCGTTTTACAACGTCGTGACTGGGAAAACCCT

M A P K K K R K V M E D P V V L Q R R D W E N P

GGCGTTACCCAACTTAATCGCCTTGCAGCACATCCCCCTTTCGCCAGCTGGCGTAATAGCGAAGAGGCCCGC

G V T Q L N R L A A H P P F A S W R N S E E A R

ACCGATCGCCCTTCCCAACAGTTGCGCAGCCTGAATGGCGAATGGCGCTTTGCCTGGTTTCCGGCACCAGAA

T D R P S Q Q L R S L N G E W R F A W F P A P E

GCGGTGCCGGAAAGCTGGCTGGAGTGCGATCTTCCTGAGGCCGATACTGTCGTCGTCCCCTCAAACTGGCAG

A V P E S W L E C D L P E A D T V V V P S N W Q

ATGCACGGTTACGATGCGCCCATCTACACCAACGTAACCTATCCCATTACGGTCAATCCGCCGTTTGTTCCC

M H G Y D A P I Y T N V T Y P I T V N P P F V P

ACGGAGAATCCGACGGGTTGTTACTCGCTCACATTTAATGTTGATGAAAGCTGGCTACAGGAAGGCCAGACG

T E N P T G C Y S L T F N V D E S W L Q E G Q T

CGAATTATTTTTGATGGCGTTAACTCGGCGTTTCATCTGTGGTGCAACGGGCGCTGGGTCGGTTACGGCCAG

R I I F D G V N S A F H L W C N G R W V G Y G Q

GACAGTCGTTTGCCGTCTGAATTTGACCTGAGCGCATTTTTACGCGCCGGAGAAAACCGCCTCGCGGTGATG

D S R L P S E F D L S A F L R A G E N R L A V M

GTGCTGCGCTGGAGTGACGGCAGTTATCTGGAAGATCAGGATATGTGGCGGATGAGCGGCATTTTCCGTGAC

V L R W S D G S Y L E D Q D M W R M S G I F R D

GTCTCGTTGCTGCATAAACCGACTACACAAATCAGCGATTTCCATGTTGCCACTCGCTTTAATGATGATTTC

V S L L H K P T T Q I S D F H V A T R F N D D F

AGCCGCGCTGTACTGGAGGCTGAAGTTCAGATGTGCGGCGAGTTGCGTGACTACCTACGGGTAACAGTTTCT

S R A V L E A E V Q M C G E L R D Y L R V T V S

TTATGGCAGGGTGAAACGCAGGTCGCCAGCGGCACCGCGCCTTTCGGCGGTGAAATTATCGATGAGCGTGGT

L W Q G E T Q V A S G T A P F G G E I I D E R G

GGTTATGCCGATCGCGTCACACTACGTCTGAACGTCGAAAACCCGAAACTGTGGAGCGCCGAAATCCCGAAT

G Y A D R V T L R L N V E N P K L W S A E I P N

CTCTATCGTGCGGTGGTTGAACTGCACACCGCCGACGGCACGCTGATTGAAGCAGAAGCCTGCGATGTCGGT

L Y R A V V E L H T A D G T L I E A E A C D V G

TTCCGCGAGGTGCGGATTGAAAATGGTCTGCTGCTGCTGAACGGCAAGCCGTTGCTGATTCGAGGCGTTAAC

F R E V R I E N G L L L L N G K P L L I R G V N

CGTCACGAGCATCATCCTCTGCATGGTCAGGTCATGGATGAGCAGACGATGGTGCAGGATATCCTGCTGATG

R H E H H P L H G Q V M D E Q T M V Q D I L L M

AAGCAGAACAACTTTAACGCCGTGCGCTGTTCGCATTATCCGAACCATCCGCTGTGGTACACGCTGTGCGAC

K Q N N F N A V R C S H Y P N H P L W Y T L C D

CGCTACGGCCTGTATGTGGTGGATGAAGCCAATATTGAAACCCACGGCATGGTGCCAATGAATCGTCTGACC

R Y G L Y V V D E A N I E T H G M V P M N R L T

GATGATCCGCGCTGGCTACCGGCGATGAGCGAACGCGTAACGCGAATGGTGCAGCGCGATCGTAATCACCCG

D D P R W L P A M S E R V T R M V Q R D R N H P

AGTGTGATCATCTGGTCGCTGGGGAATGAATCAGGCCACGGCGCTAATCACGACGCGCTGTATCGCTGGATC

S V I I W S L G N E S G H G A N H D A L Y R W I

AAATCTGTCGATCCTTCCCGCCCGGTGCAGTATGAAGGCGGCGGAGCCGACACCACGGCCACCGATATTATT

K S V D P S R P V Q Y E G G G A D T T A T D I I

TGCCCGATGTACGCGCGCGTGGATGAAGACCAGCCCTTCCCGGCTGTGCCGAAATGGTCCATCAAAAAATGG

C P M Y A R V D E D Q P F P A V P K W S I K K W

CTTTCGCTACCTGGAGAGACGCGCCCGCTGATCCTTTGCGAATACGCCCACGCGATGGGTAACAGTCTTGGC

L S L P G E T R P L I L C E Y A H A M G N S L G

GGTTTCGCTAAATACTGGCAGGCGTTTCGTCAGTATCCCCGTTTACAGGGCGGCTTCGTCTGGGACTGGGTG

G F A K Y W Q A F R Q Y P R L Q G G F V W D W V

```
GATCAGTCGCTGATTAAATATGATGAAAACGGCAACCCGTGGTCGGCTTACGGCGGTGATTTTGGCGATACG
 D  Q  S  L  I  K  Y  D  E  N  G  N  P  W  S  A  Y  G  G  D  F  G  D  T
CCGAACGATCGCCAGTTCTGTATGAACGGTCTGGTCTTTGCCGACCGCACGCCGCATCCAGCGCTGACGGAA
 P  N  D  R  Q  F  C  M  N  G  L  V  F  A  D  R  T  P  H  P  A  L  T  E
GCAAAACACCAGCAGCAGTTTTTCCAGTTCCGTTTATCCGGGCAAACCATCGAAGTGACCAGCGAATACCTG
 A  K  H  Q  Q  Q  F  F  Q  F  R  L  S  G  Q  T  I  E  V  T  S  E  Y  L
TTCCGTCATAGCGATAACGAGCTCCTGCACTGGATGGTGGCGCTGGATGGTAAGCCGCTGGCAAGCGGTGAA
 F  R  H  S  D  N  E  L  L  H  W  M  V  A  L  D  G  K  P  L  A  S  G  E
GTGCCTCTGGATGTCGCTCCACAAGGTAAACAGTTGATTGAACTGCCTGAACTACCGCAGCCGGAGAGCGCC
 V  P  L  D  V  A  P  Q  G  K  Q  L  I  E  L  P  E  L  P  Q  P  E  S  A
GGGCAACTCTGGCTCACAGTACGCGTAGTGCAACCGAACGCGACCGCATGGTCAGAAGCCGGGCACATCAGC
 G  Q  L  W  L  T  V  R  V  V  Q  P  N  A  T  A  W  S  E  A  G  H  I  S
GCCTGGCAGCAGTGGCGTCTGGCGGAAAACCTCAGTGTGACGCTCCCCGCCGCGTCCCACGCCATCCCGCAT
 A  W  Q  Q  W  R  L  A  E  N  L  S  V  T  L  P  A  A  S  H  A  I  P  H
CTGACCACCAGCGAAATGGATTTTTGCATCGAGCTGGGTAATAAGCGTTGGCAATTTAACCGCCAGTCAGGC
 L  T  T  S  E  M  D  F  C  I  E  L  G  N  K  R  W  Q  F  N  R  Q  S  G
TTTCTTTCACAGATGTGGATTGGCGATAAAAAACAACTGCTGACGCCGCTGCGCGATCAGTTCACCCGTGCA
 F  L  S  Q  M  W  I  G  D  K  K  Q  L  L  T  P  L  R  D  Q  F  T  R  A
CCGCTGGATAACGACATTGGCGTAAGTGAAGCGACCCGCATTGACCCTAACGCCTGGGTCGAACGCTGGAAG
 P  L  D  N  D  I  G  V  S  E  A  T  R  I  D  P  N  A  W  V  E  R  W  K
GCGGCGGGCCATTACCAGGCCGAAGCAGCGTTGTTGCAGTGCACGGCAGATACACTTGCTGATGCGGTGCTG
 A  A  G  H  Y  Q  A  E  A  A  L  L  Q  C  T  A  D  T  L  A  D  A  V  L
ATTACGACCGCTCACGCGTGGCAGCATCAGGGGAAAACCTTATTTATCAGCCGGAAAACCTACCGGATTGAT
 I  T  T  A  H  A  W  Q  H  Q  G  K  T  L  F  I  S  R  K  T  Y  R  I  D
GGTAGTGGTCAAATGGCGATTACCGTTGATGTTGAAGTGGCGAGCGATACACCGCATCCGGCGCGGATTGGC
 G  S  G  Q  M  A  I  T  V  D  V  E  V  A  S  D  T  P  H  P  A  R  I  G
CTGAACTGCCAGCTGGCGCAGGTAGCAGAGCGGGTAAACTGGCTCGGATTAGGGCCGCAAGAAAACTATCCC
 L  N  C  Q  L  A  Q  V  A  E  R  V  N  W  L  G  L  G  P  Q  E  N  Y  P
GACCGCCTTACTGCCGCCTGTTTTGACCGCTGGGATCTGCCATTGTCAGACATGTATACCCCGTACGTCTTC
 D  R  L  T  A  A  C  F  D  R  W  D  L  P  L  S  D  M  Y  T  P  Y  V  F
CCGAGCGAAAACGGTCTGCGCTGCGGGACGCGCGAATTGAATTATGGCCCACACCAGTGGCGCGGCGACTTC
 P  S  E  N  G  L  R  C  G  T  R  E  L  N  Y  G  P  H  Q  W  R  G  D  F
CAGTTCAACATCAGCCGCTACAGTCAACAGCAACTGATGGAAACCAGCCATCGCCATCTGCTGCACGCGGAA
 Q  F  N  I  S  R  Y  S  Q  Q  Q  L  M  E  T  S  H  R  H  L  L  H  A  E
GAAGGCACATGGCTGAATATCGACGGTTTCCATATGGGGATTGGTGGCGACGACTCCTGGAGCCCGTCAGTA
 E  G  T  W  L  N  I  D  G  F  H  M  G  I  G  G  D  D  S  W  S  P  S  V
TCGGCGGAATTCCAGCTGAGCGCCGGTCGCTACCATTACCAGTTGGTCTGGTGTCAAAAATAA
 S  A  E  F  Q  L  S  A  G  R  Y  H  Y  Q  L  V  W  C  Q  K  *
```

TAATAAGCTAGCTGATCATAATCAGCCATACCACATTTGTAGAGGTTTTACTTGCTTTAAAAAACCTCCCACACCTCCCCCTGAACCTGAAACATAAAATGAATGCAA
TTGTTGTTGTTAACTTGTTTATTGCAGCTTATAATGGTTACAAATAAAGCAATAGCATCACAAATTTCACAAATAAAGCATTTTTTTCACTGCATTCTAGTTGTGGTT
TGTCCAAACTCATCAATGTATCTTAATTAA**GAAGTTCCTAT**ACTTACTAGAGA**ATAGGAACTTC**GGAATAGGAACTTCTTTAATTAAGCTAGCGGCGCGCCGCGGCCG
CAGTACTGAT

Fig. S7. Mouse *Fam83h* Wild-Type and NLS-*lac*Z Knockin Sequences. ***A:*** The NCBI genomic reference sequence NC_000081.6 for mouse strain C57BL/6J starts with the first nucleotide of exon 1a, which is found on the 5' end transcript variant 2 (TV2) and ends 1 bp after exon 5. The entire coding region and all but a single nucleotide of the 3' untranslated region (highlighted in yellow) was deleted. ***B:*** The NLS-*lac*Z coding sequence and 3' untranslated region that included two polyadenylation signals (AATAAA, underlined) and the FRT sequence (bold) that remainedfollowing flippase recombination. Thus the wild-type mice differ only by the replacement of the sequence in yellow (in **A**) with the sequence in blue (in **B**).

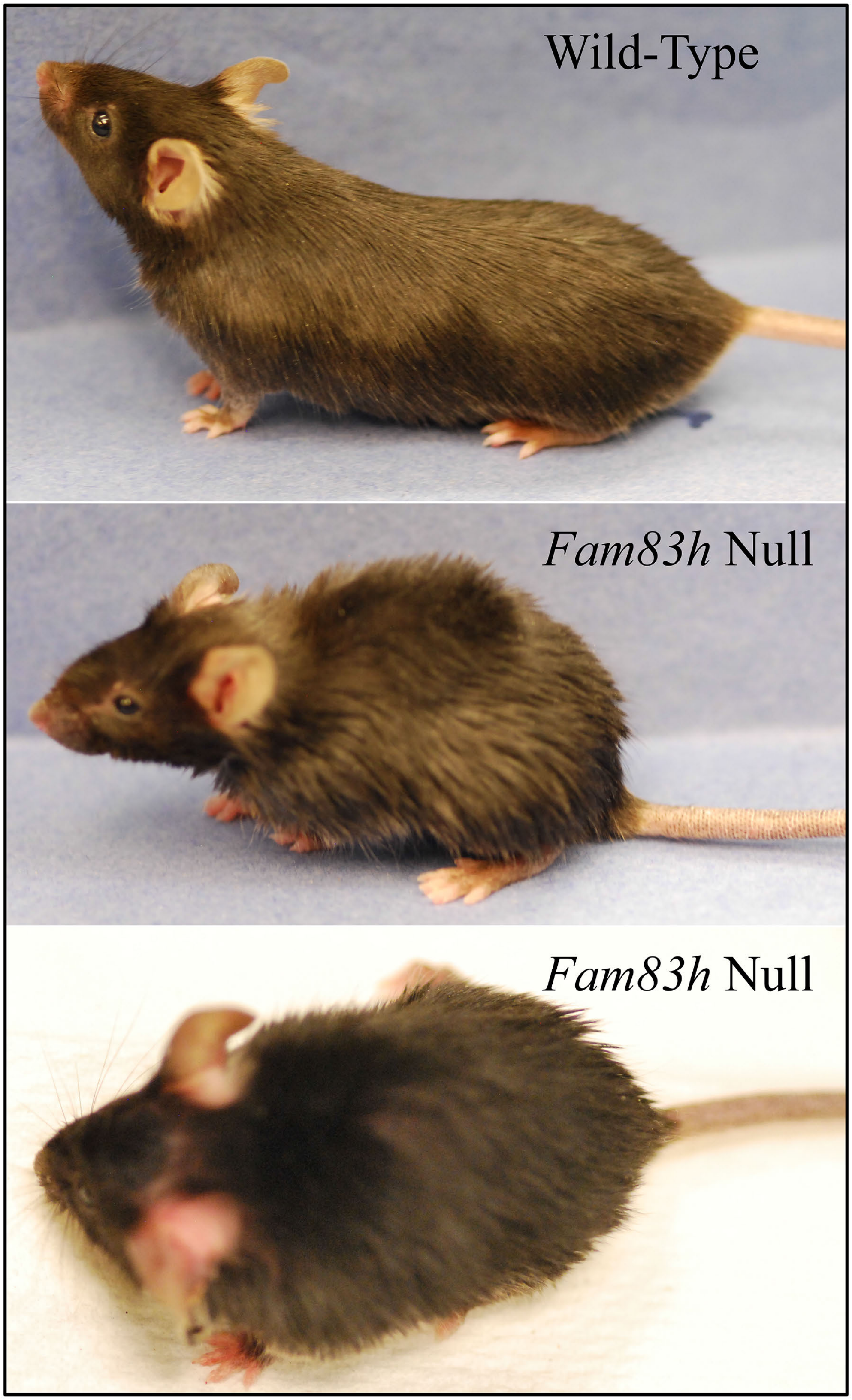

Fig. S8. Wild-type and *Fam83h* null mice at 7-weeks. The *Fam83h* null mice that survive to 7-weeks are smaller than the wild-type. Their fur tends to shed and coat hair gets caught up in the gingival crevice of the mandibular incisors.

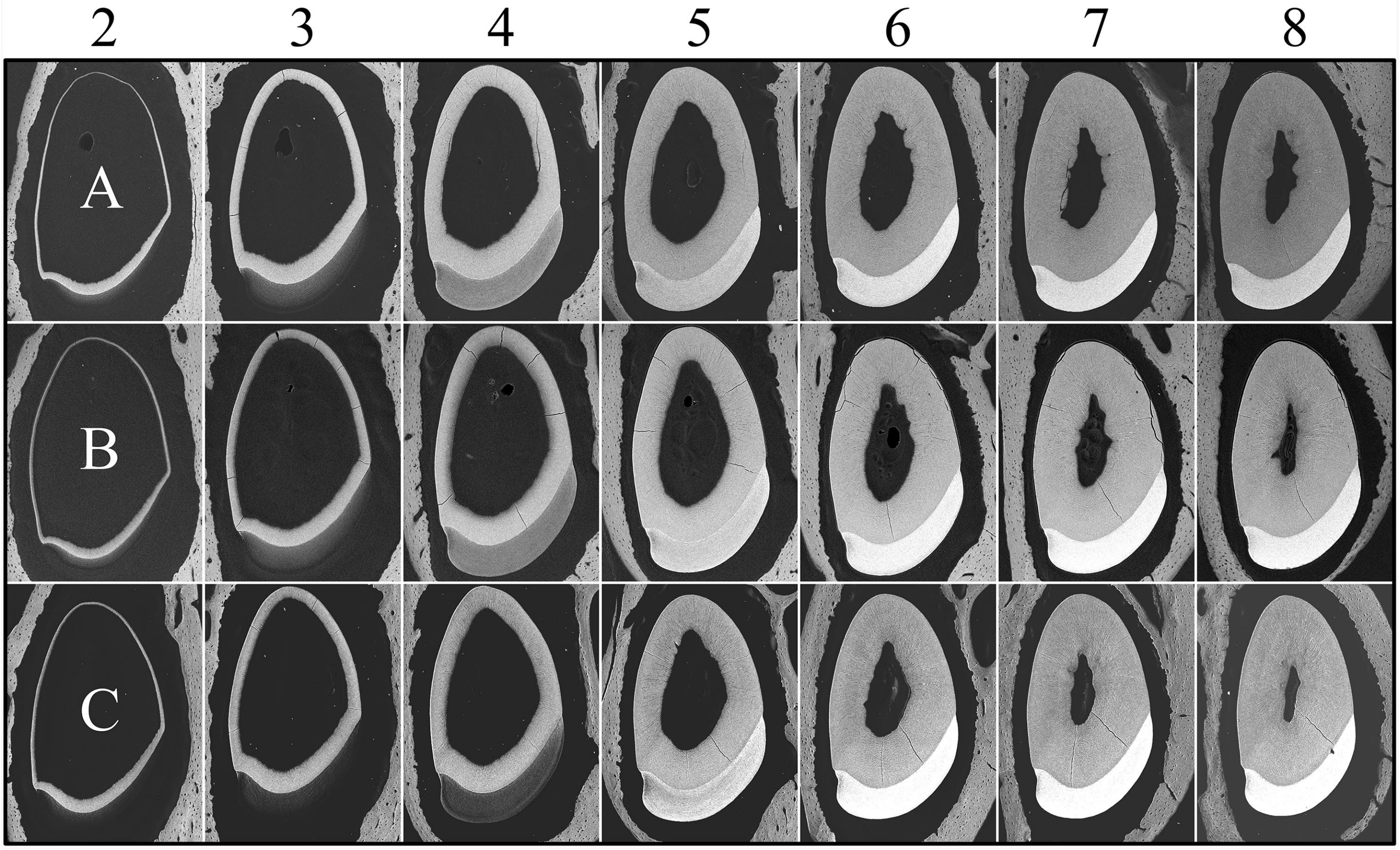

Fig. S9. bSEM Images of Manibular Incisor Cross Sections at 7-weeks (lower magnification). Mandibular incisors display all stages of enamel formation. The incisors are cross-sectioned at 1 mm intervals and examined by bSEM. The basal ends are on the left. ***A:*** wild-type; ***B:*** *Fam83h*$^{+/-}$; ***C:*** *Fam83h*$^{-/-}$. Levels 2 and 3 show the secretory stage of amelogenesis when the enamel layer is expanding. Levels 4 through 8 show the maturation stage when the enamel layer no longer expands. During this stage the mineral ribbons deposited during the secretory stage grow in width and thickness and the enamel layer becomes increasingly mineralized. Level 8 is where the incisor reaches the level of the alveolar crest, still prior to eruption. The enamel layer appears to be fully mineralized in all 3 genotypes. The pulp space in the null mouse is smaller, suggesting that the incisors erupted more slowly than in the wild-type (giving the odontoblasts more time to add dentin).

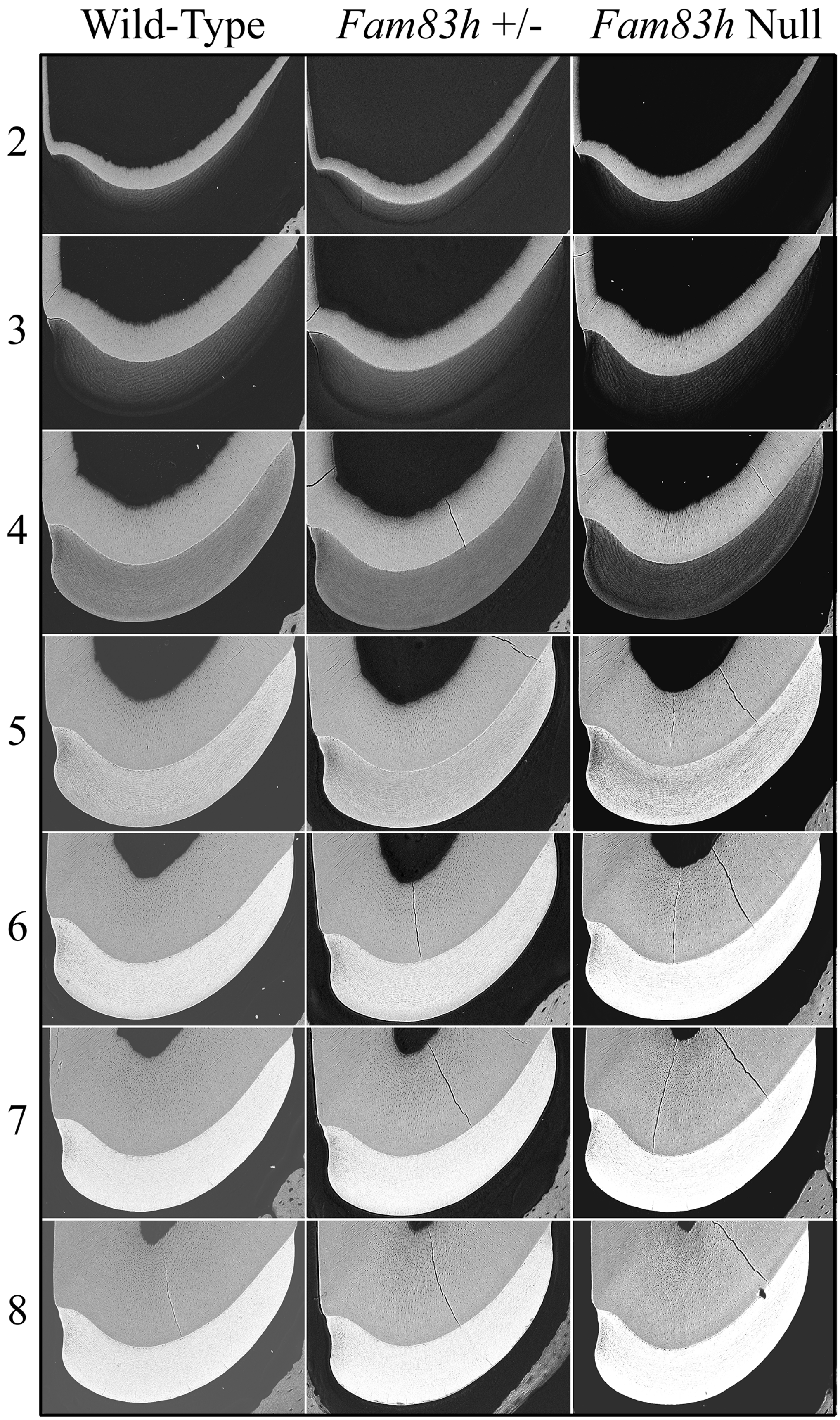

Fig. S10. bSEM Images of Manibular Incisor Cross Sections at 7-weeks (higher magnification). The enamel layer appears to be fully mineralized in all 3 genotypes.

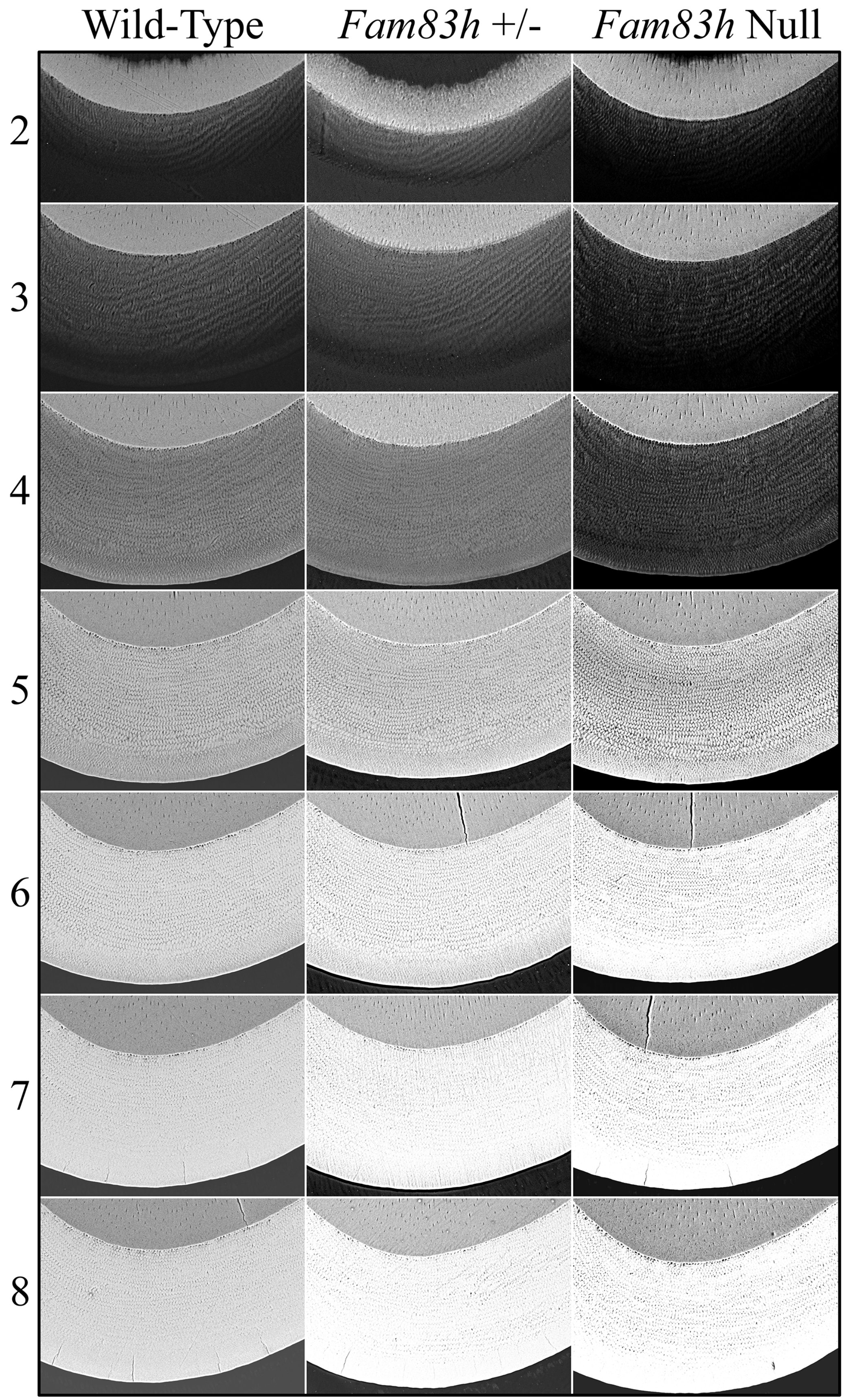

Fig. S11. bSEM Images of Manibular Incisor Cross Sections at 7-weeks (highest magnification). The enamel layer appears to be fully mineralized in all 3 genotypes. The enamel thickness is the same, suggesting normal development during the secretory stage.

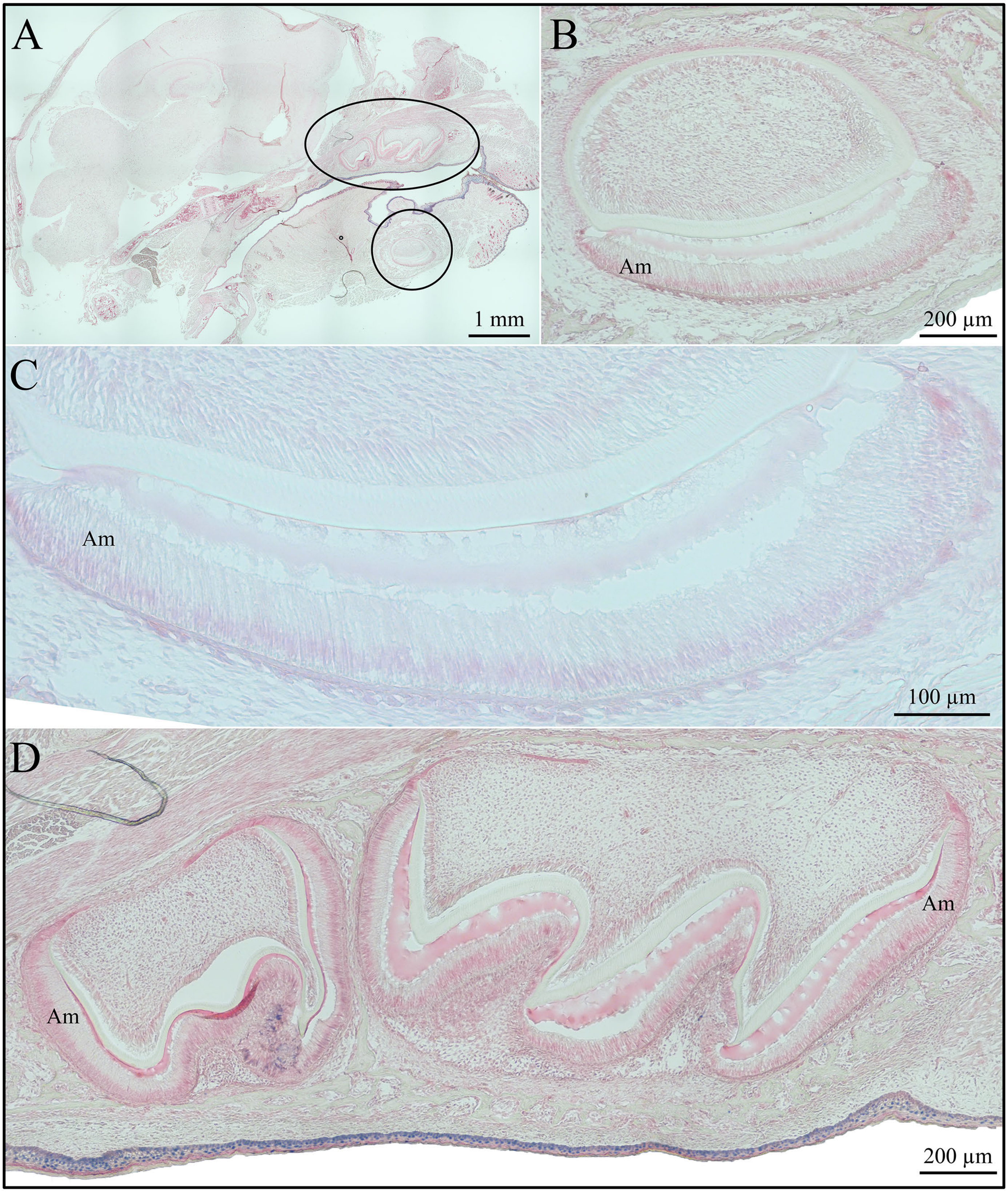

Fig. S12. *LacZ* Histochemistry of Developing PN5 *Fam83h*$^{+/-}$ Mouse Teeth. ***A:*** Low Magnification view of sagittal section of the head. The teeth are circled. ***B:*** Higher magnification of the mandibular incisor near cross-section. ***C:*** Higher magnification of the incisor cross-section. No X-gal histostaining is observed in the secretory stage ameloblasts (Am). ***D:*** PN5 *Fam83h*$^{+/-}$ mouse maxillary first and second molars. The secretory stage ameloblasts are negative except for the mesial cusp tip of the second molar. Note the positive (blue) staining in the nuclei of the oral mucosa.

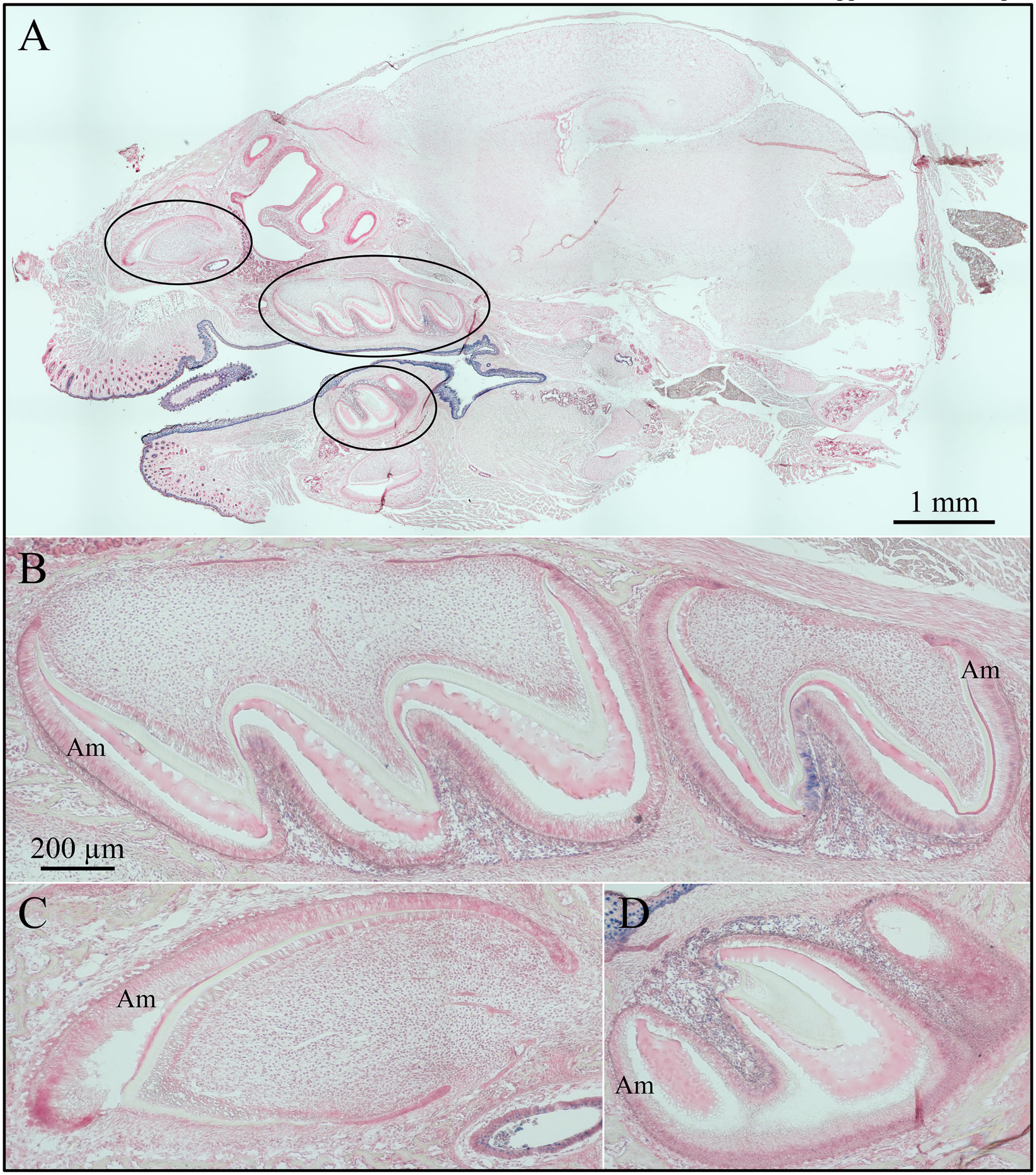

Fig. S13. *Lac*Z Histochemistry of Developing PN5 *Fam83h* Null Mouse Teeth. ***A:*** Low Magnification view of sagittal section of the head. The teeth are circled. ***B:*** Higher magnification of the maxillary molars. Positive staining is observed in Ameloblasts on the mesial cusp of the 2nd molar. ***C:*** Higher magnification of the maxillary incisor. No X-gal histostaining is observed in the secretory stage ameloblasts (Am). ***D:*** Mandibular first molar showing only trace X-gal staining in Ameloblasts. Note the positive (blue) staining in the nuclei of the oral mucosa, which provides an internal positive control.

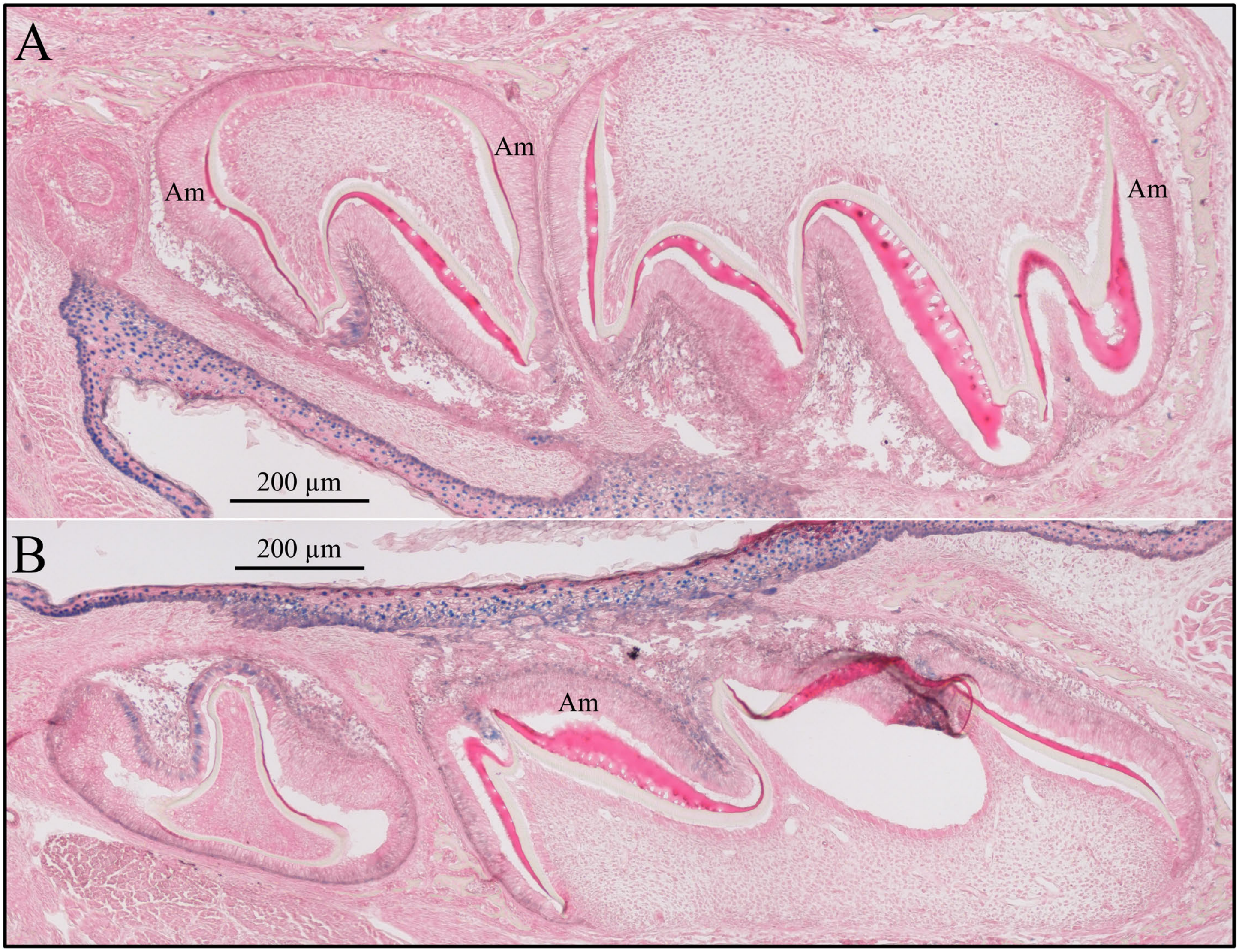

Fig. S14. *Lac*Z Histochemistry of Developing PN6 *Fam83h* Null Mouse Teeth. ***A:*** Low Magnification view of the developing maxillary molars. The ameloblasts (Am) in these teeth are in the secretory stage of amelogenesis. No X-gal stain is observed in the maxillary first molar. Staining observed in ameloblasts at the distal cusp tip. Oral mucosa nuclei stain positive. ***B:*** Low magnification view of the developing mandibular molars. Most ameloblasts are negative except at the tip of the distal cusp in the first molar and the cusp slopes of the second molar and on the tip of the mesial cusp.

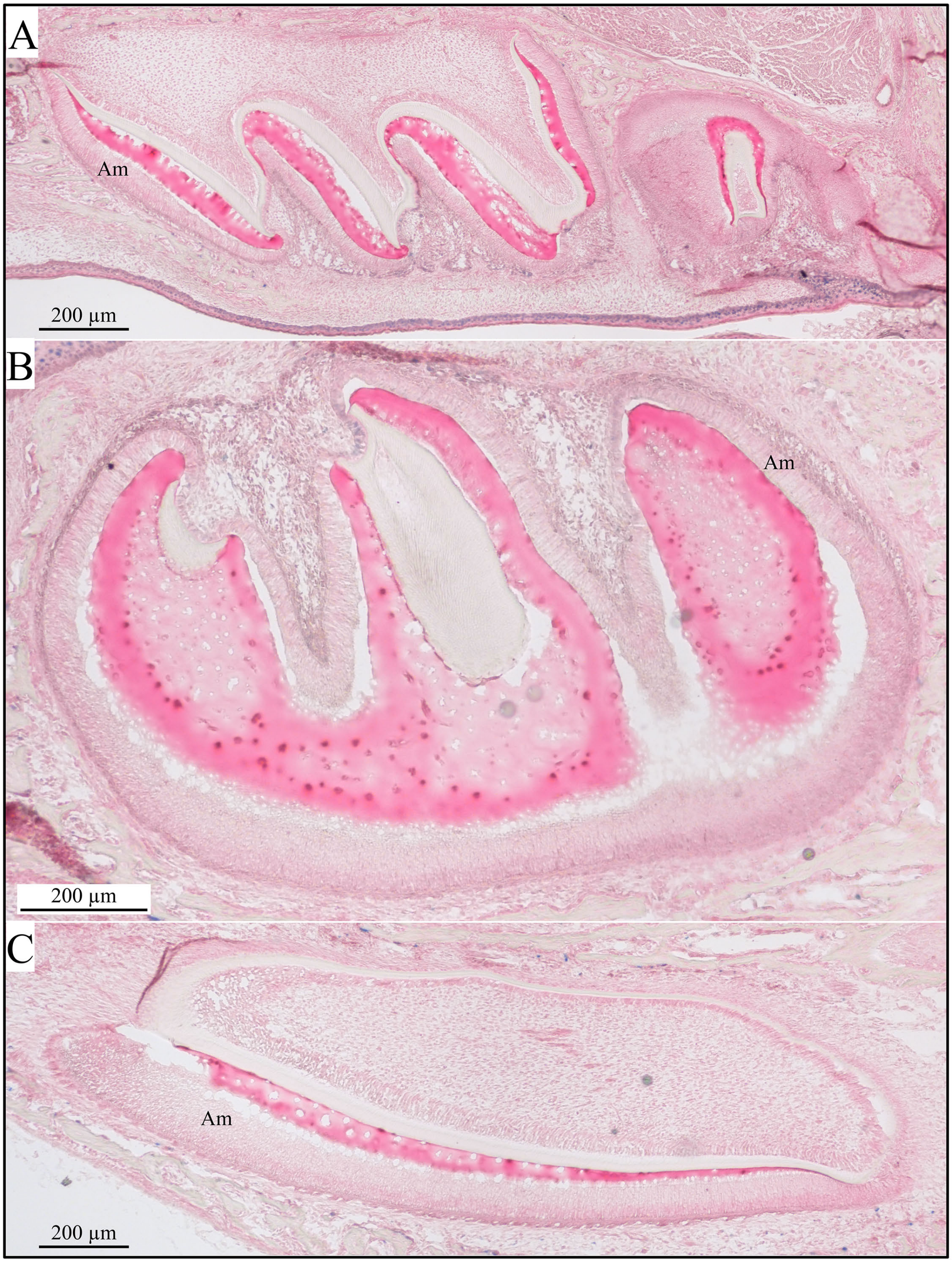

Fig. S15. *Lac*Z Histochemistry of Developing PN9 *Fam83h* Null Mouse Teeth. ***A-B:*** Low Magnification views of the developing maxillary and mandibular first molars, which have ameloblasts (Am) in the maturation stage of amelogenesis. ***C:*** Low magnification view of the basal end developing mandibular incisor. No X-gal stain is observed in the maxillary first molar. Most ameloblasts are negative except at the tip of the distal cusp in the first molar and the cusp slopes of the second molar and on the tip of the mesial cusp.

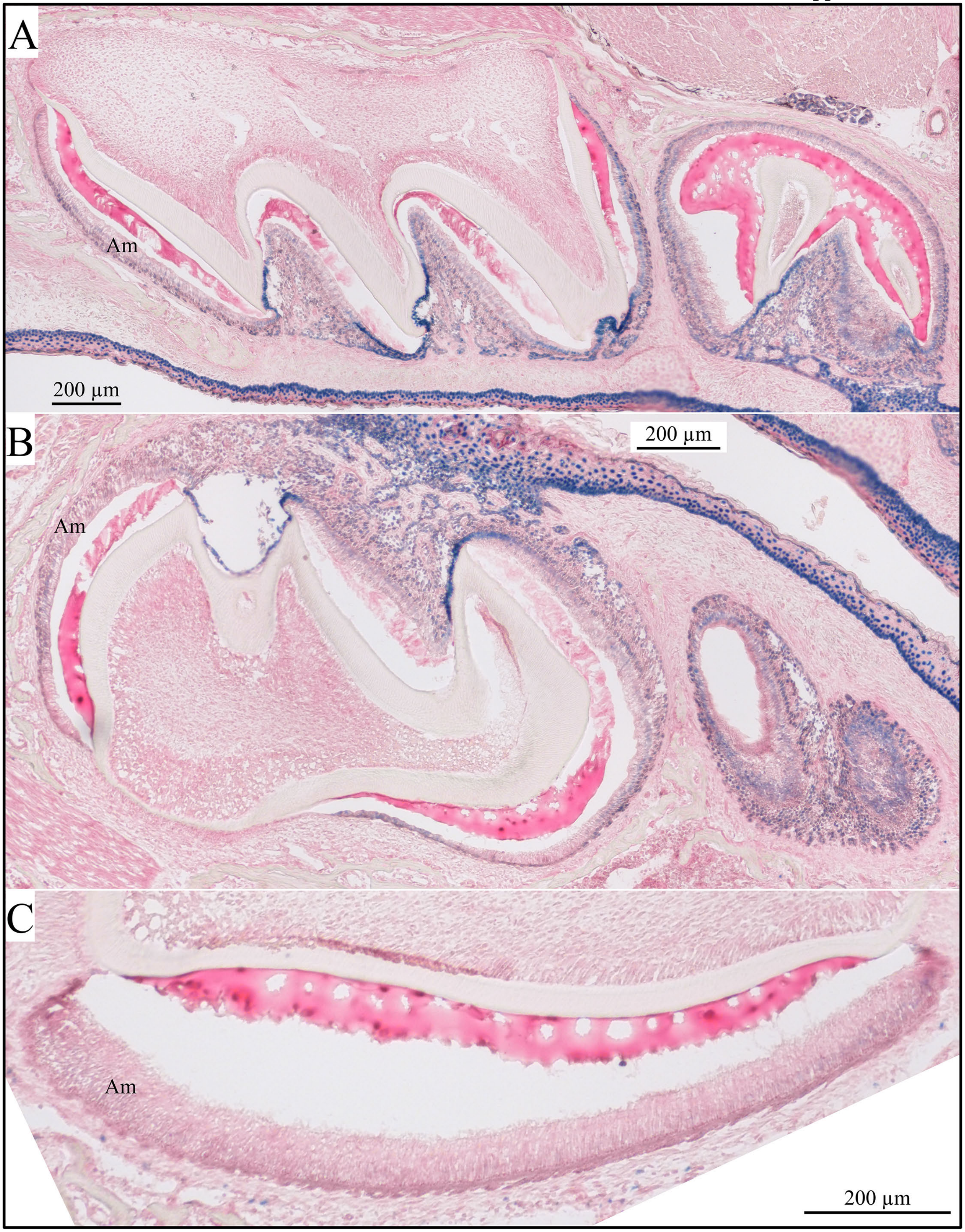

Fig. S16. *Lac*Z Histochemistry of Developing PN11 *Fam83h* Null Mouse Teeth. ***A-B:*** Low Magnification views of the developing maxillary and mandibular first molars, which have ameloblasts (Am) in the maturation stage of amelogenesis. ***C:*** Low magnification view of the basal end developing mandibular incisor. No X-gal stain is observed in the maxillary first molar. Most ameloblasts are negative except at the tip of the distal cusp in the first molar and the cusp slopes of the second molar and on the tip of the mesial cusp.

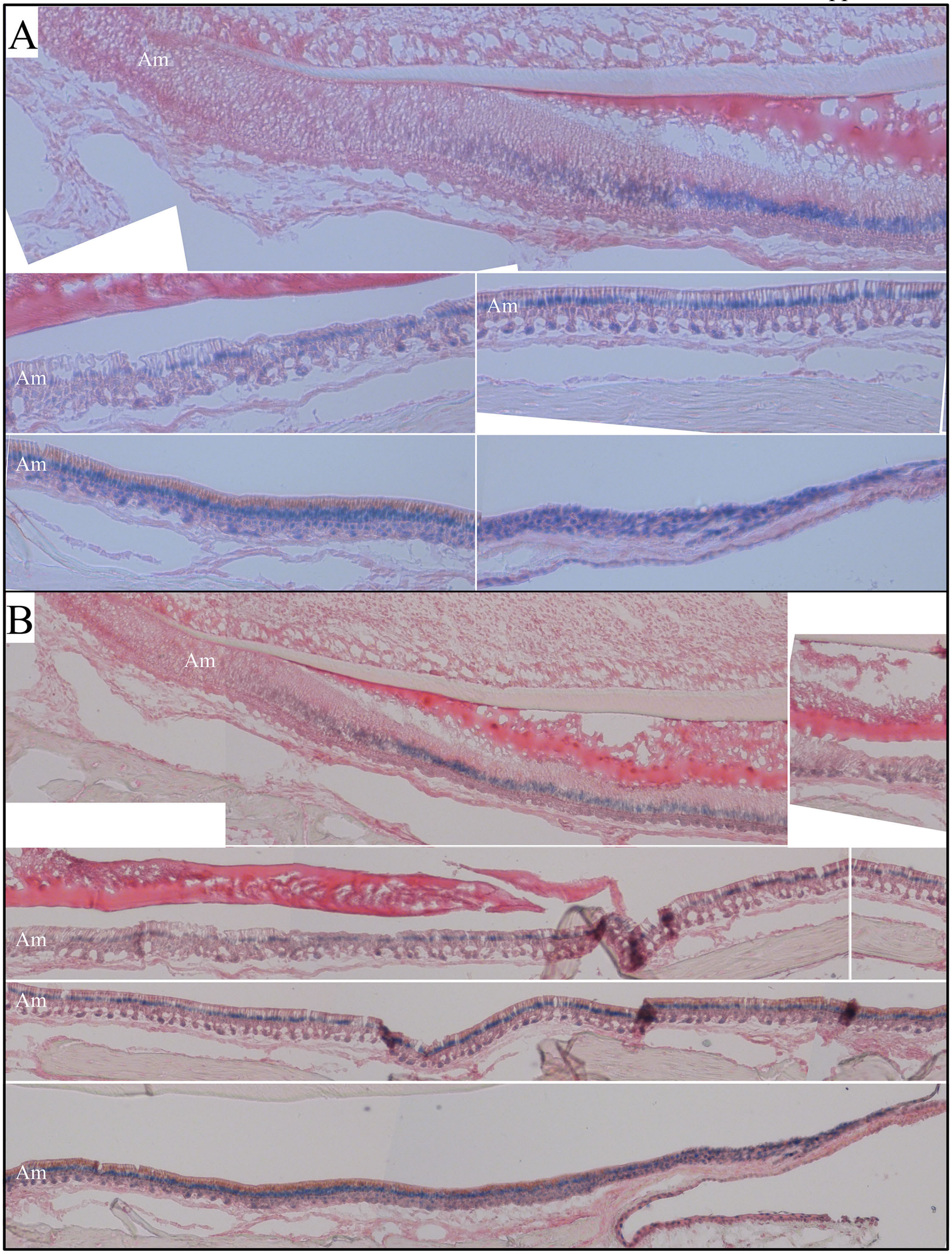

Fig. S17. *Lac*Z Histochemistry of 7-Week *Fam83h*$^{+/-}$ Mandibular Incisors. ***A-B:*** Low Magnification views of the developing maxillary and mandibular first molars, which have ameloblasts (Am) in the maturation stage of amelogenesis. ***C:*** Low magnification view of the basal end developing mandibular incisor. No X-gal stain is observed in the maxillary first molar. Most ameloblasts are negative except at the tip of the distal cusp in the first molar and the cusp slopes of the second molar and on the tip of the mesial cusp.

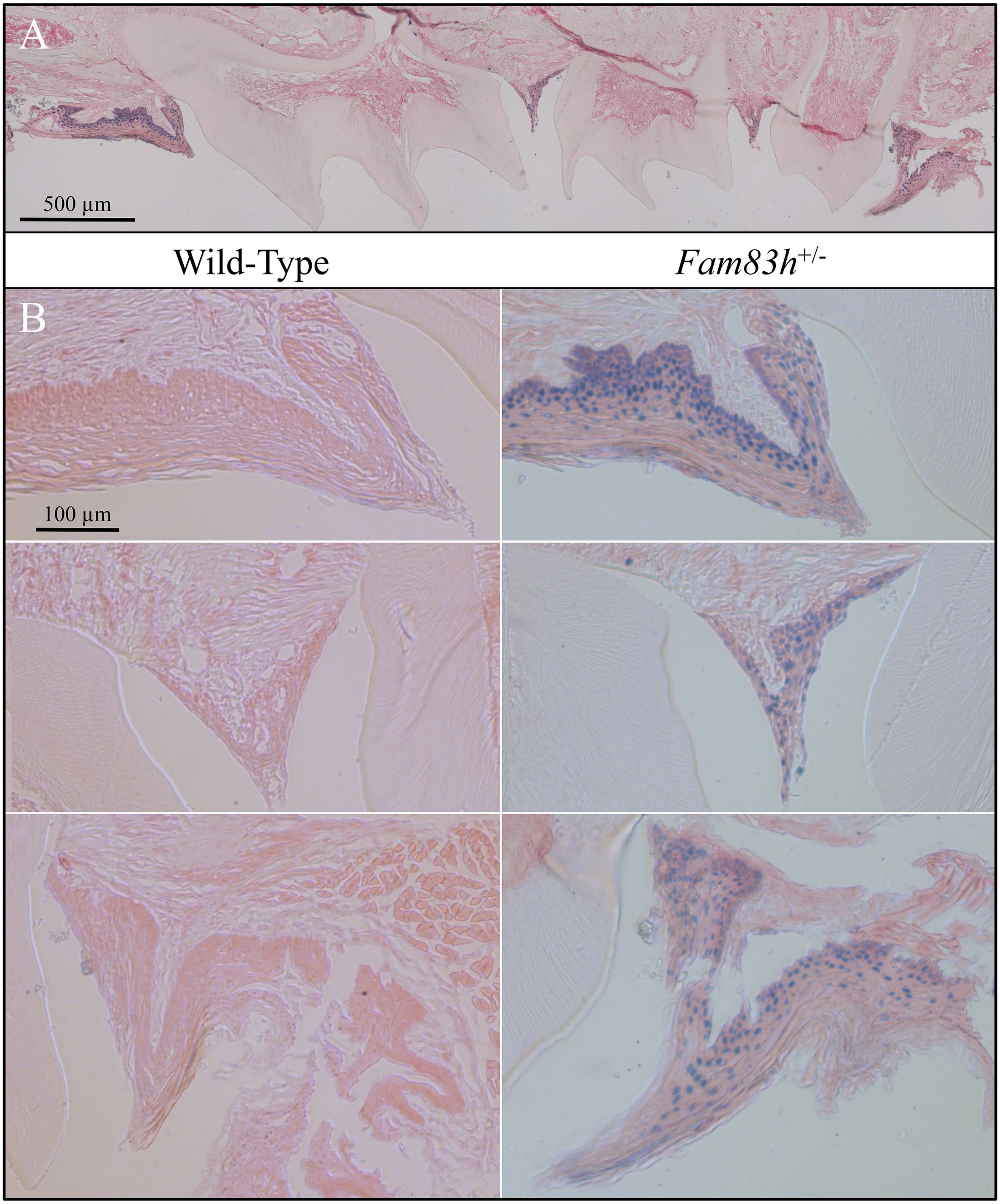

Fig. S18. *Lac*Z Histochemistry of D28 *Fam83h*$^{+/-}$ Dental Papilla. ***A:*** X-gal stained D28 *Fam83h*$^{+/-}$ cryosection of the erupted maxiallary molars. ***B:*** High magnification views X-gal stained cryosections from D28 wild-type and *Fam83h*$^{+/-}$ mice. The gingival epithelium stains positive.

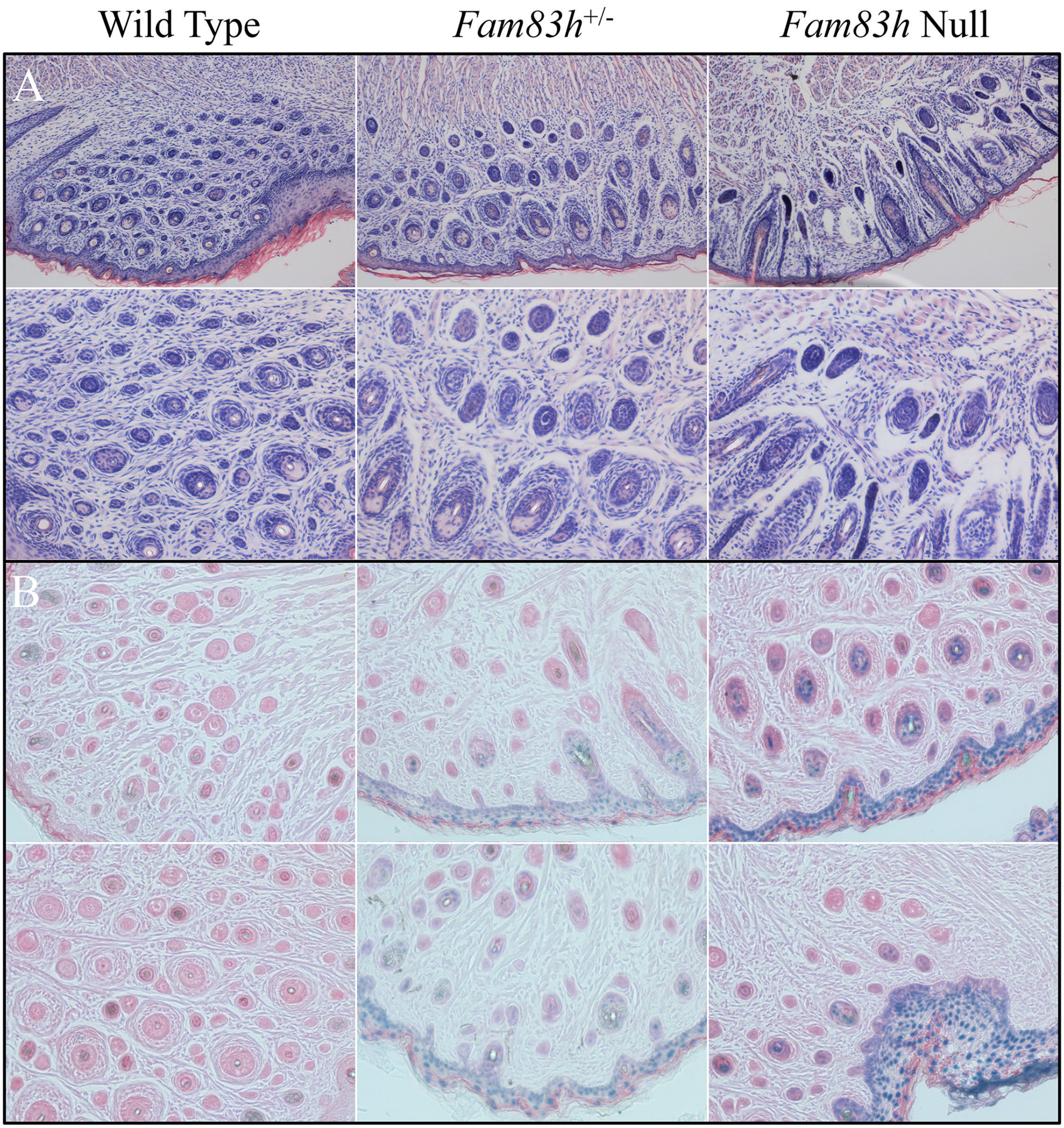

Fig. S19. Histology and *Lac*Z Histochemistry of PN5 Perioral Skin. ***A:*** H&E stained sections showed a decreasing number of vibrissae. The sebaceous glands look under-developed and disorganized. ***B:*** X-gal stained sections reported *Fam83h* expression in the cortex and root sheath areas of the vibrissae. All cell layers of the epidermis reported positive for *Lac*Z stain, including the stratum spinosum and stratum basale.
