# Supplementary material for: Fam83h null mice support a neomorphic mechanism for human ADHCAI
Source: Mol Genet Genomic Med. 2015 Sep 21;4(1):46–67. doi: 10.1002/mgg3.178 (PMC4707031; doi:10.1002/mgg3.178)
Supplement: Supplementary file 2 — Figure S20. LacZ Histochemistry of Tongue at 7 Weeks. Figure S21. LacZ Histochemistry of Submandibular Salivary Gland at 7 Weeks. Figure S22. LacZ Histochemistry of Thymus at 7 Weeks. Figure S23. LacZ Histochemistry of Urinary Bladder at 7 Weeks. Figure S24. LacZ Histochemistry of Oviduct at 7 Weeks. Figure S25. LacZ Histochemistry of Testis at 7 Weeks. Figure S26. LacZ Histochemistry of Uterus at 7 Weeks. Figure S27. LacZ Histochemistry of Ovary at 7 Weeks. Figure S28. LacZ Histochemistry of Prostate at 7 Weeks (Part 1). Figure S29. LacZ Histochemistry of Prostate at 7 Weeks (Part 2). Figure S30. Histology of Mouse Maxillary First Molars at PN5. Figure S31. Histology of Mouse Maxillary First Molars at PN11. Figure S32. Histology of Fam83h null Mandibular Incisor at 7 weeks. Figure S33. SDS‐PAGE and western Blots of enamel proteins. Figure S34. Proteins that immunoprecipitated with FAM83H. Figure S35. Protein sequence alignment of FAM83H orthologs from eight vertebrates. Figure S36. FAM83H phosphorylation by CK1 in vitro. Figure S37. FAM83H Exon 5 PCR primers and reaction conditions. [file MGG3-4-046-s002.pdf]

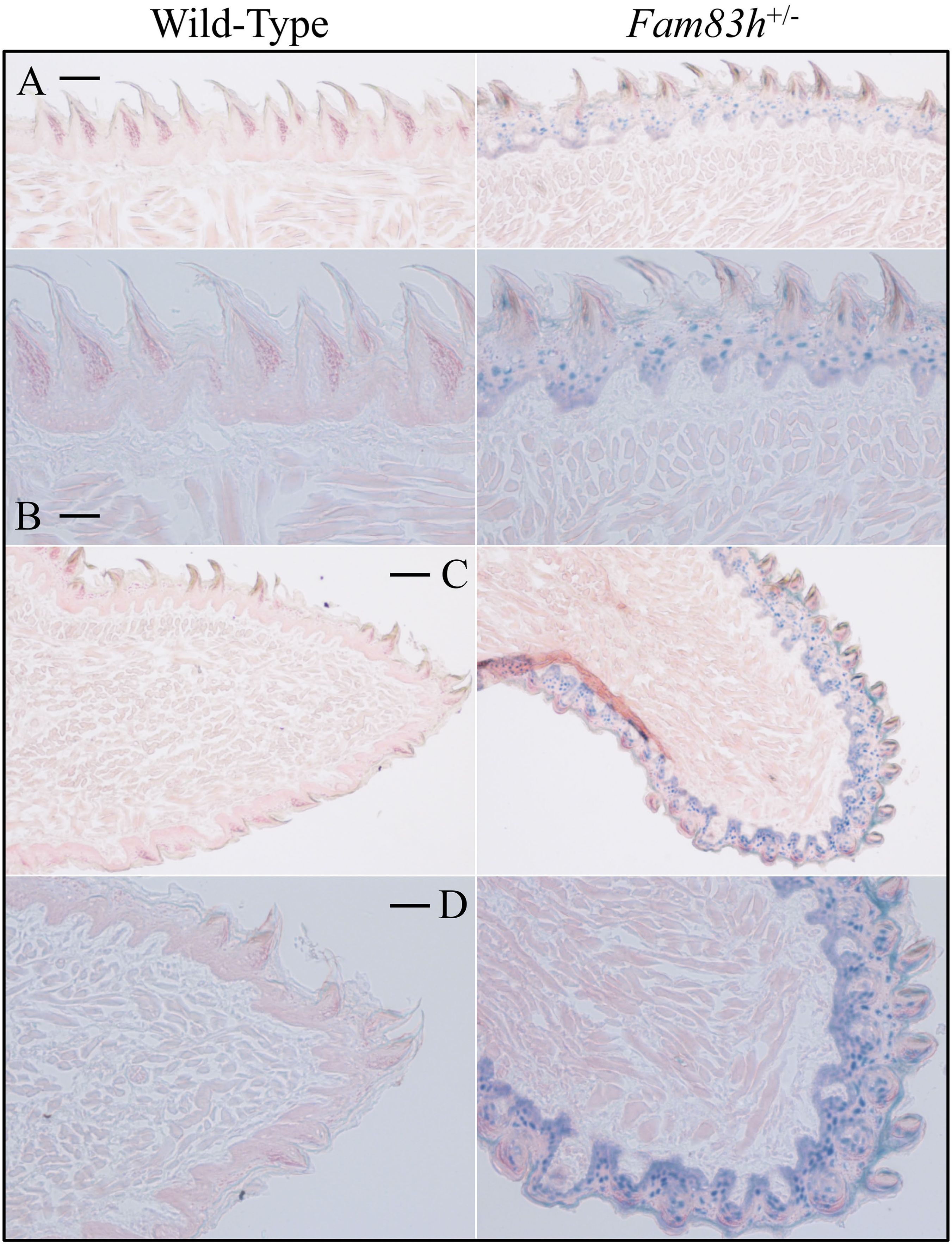

Fig. S20. *Lac*Z Histochemistry of Tongue at 7-Weeks. The tongue dorsal epithelium stained positive. ***A:*** Scale bar 100 μm. ***B:*** Scale bar 50 μm. ***C:*** Scale bar 100 μm. ***D:*** Scale bar 50 μm.

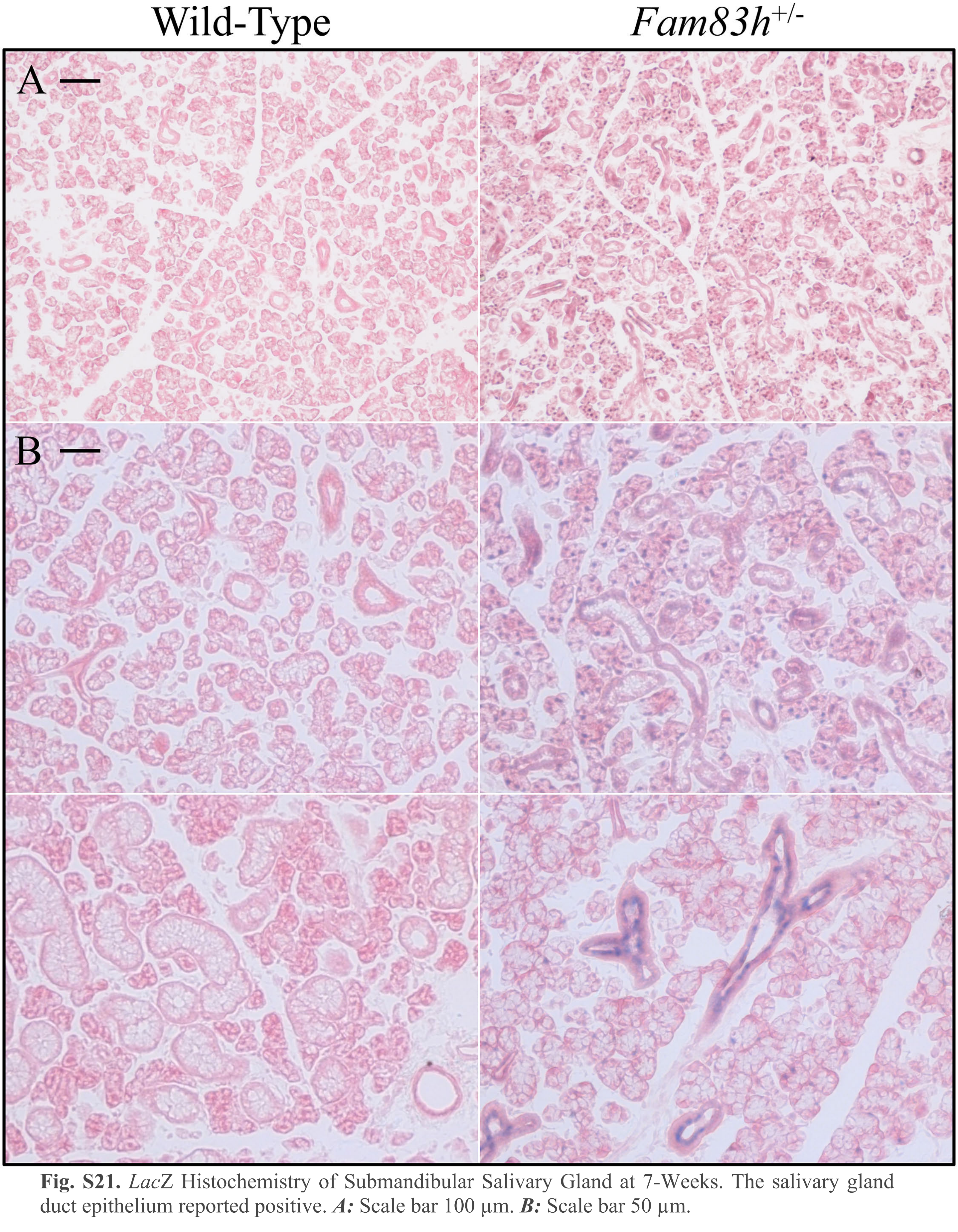

Fig. S21. *Lac*Z Histochemistry of Submandibular Salivary Gland at 7-Weeks. The salivary gland duct epithelium reported positive. ***A:*** Scale bar 100 µm. ***B:*** Scale bar 50 µm.

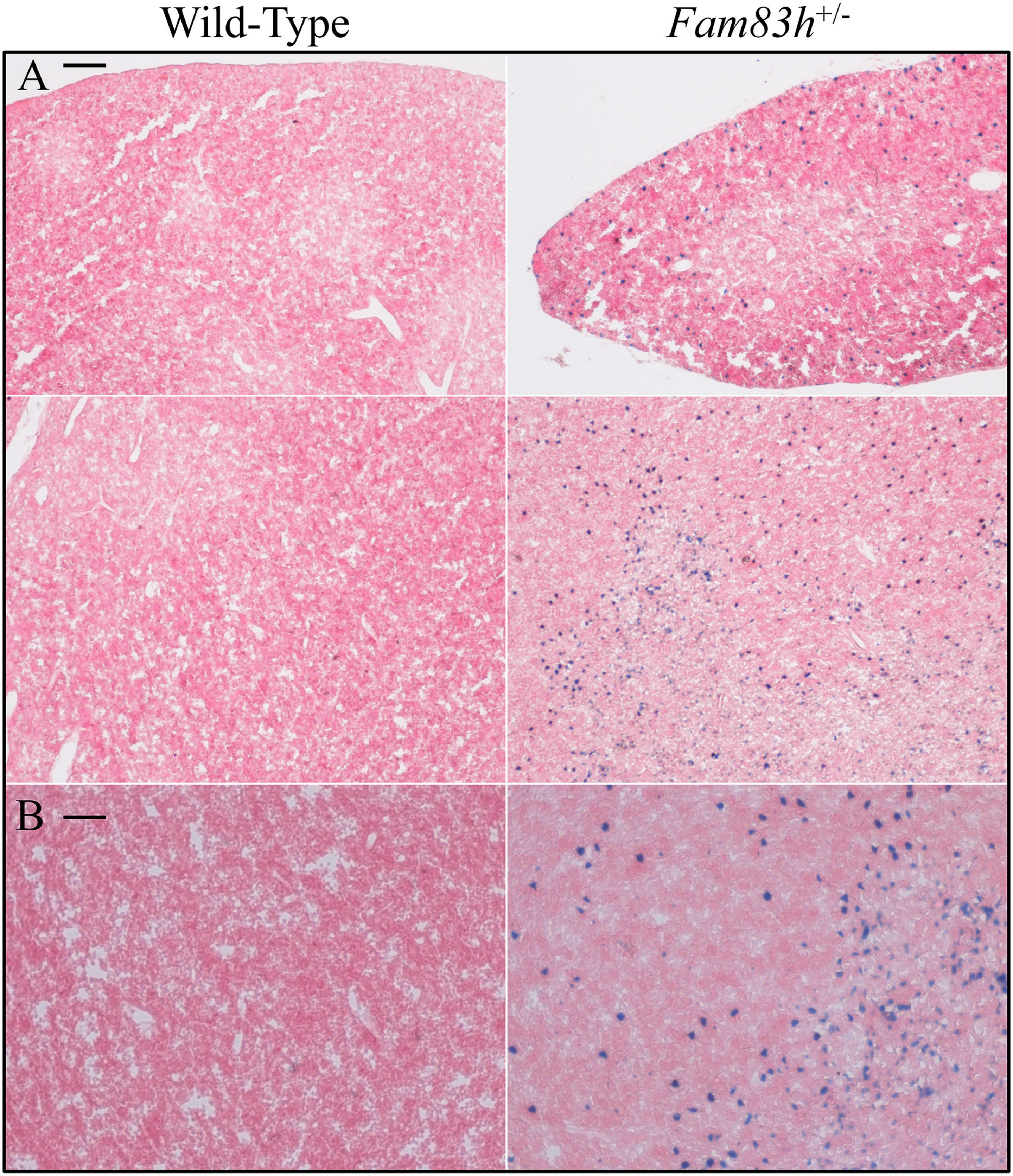

Fig. S22. *Lac*Z Histochemistry of Thymus at 7-Weeks. Selected cells in the thymus reported positive. ***A:*** Scale bar 100 µm. ***B:*** Scale bar 50 µm.

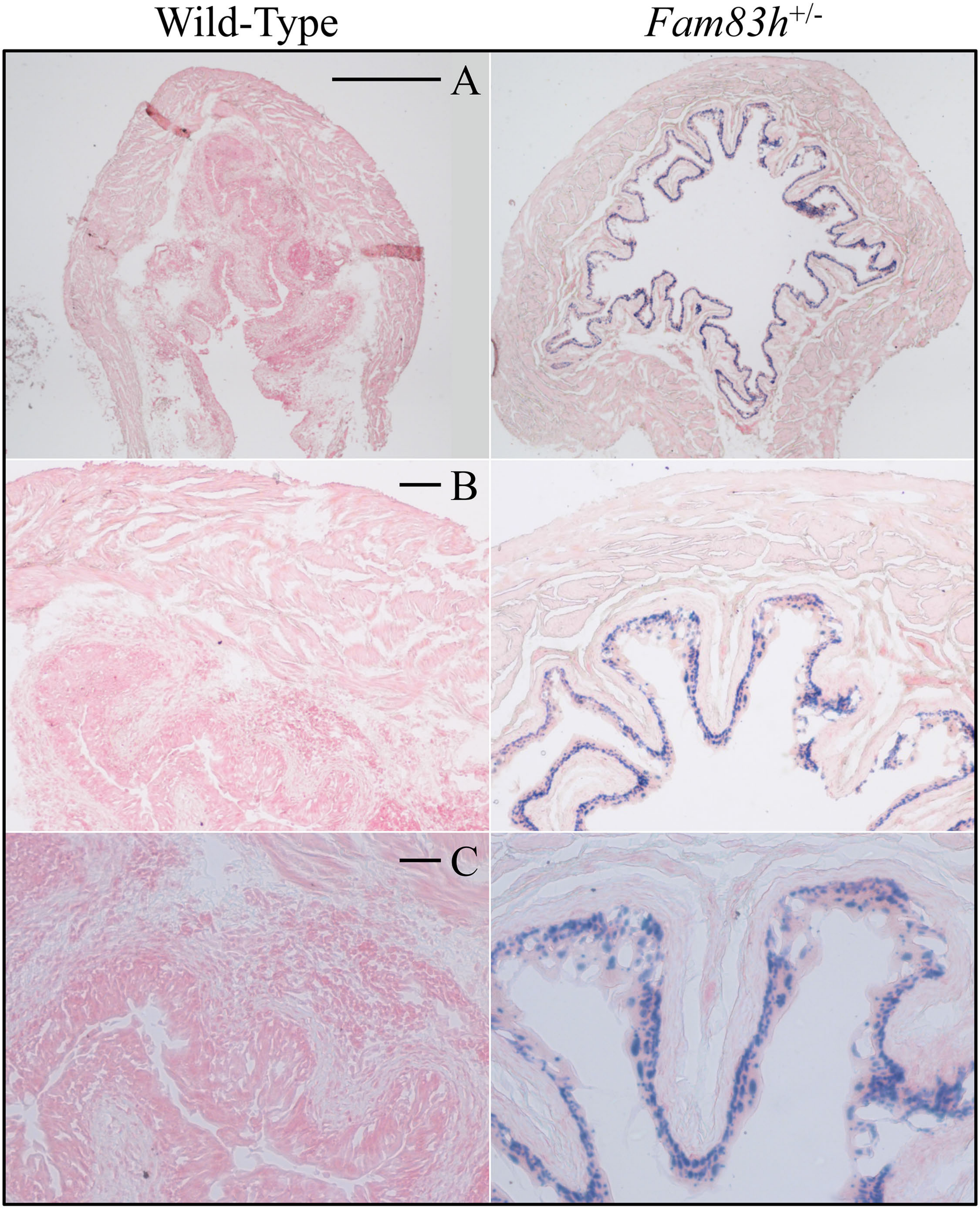

Fig. S23. *LacZ* Histochemistry of Urinary Bladder at 7-Weeks. The urinary epithelium reported postitive. ***A:*** Scale bar 500 µm. ***B:*** Scale bar 100 µm. ***C:*** Scale bar 50 µm.

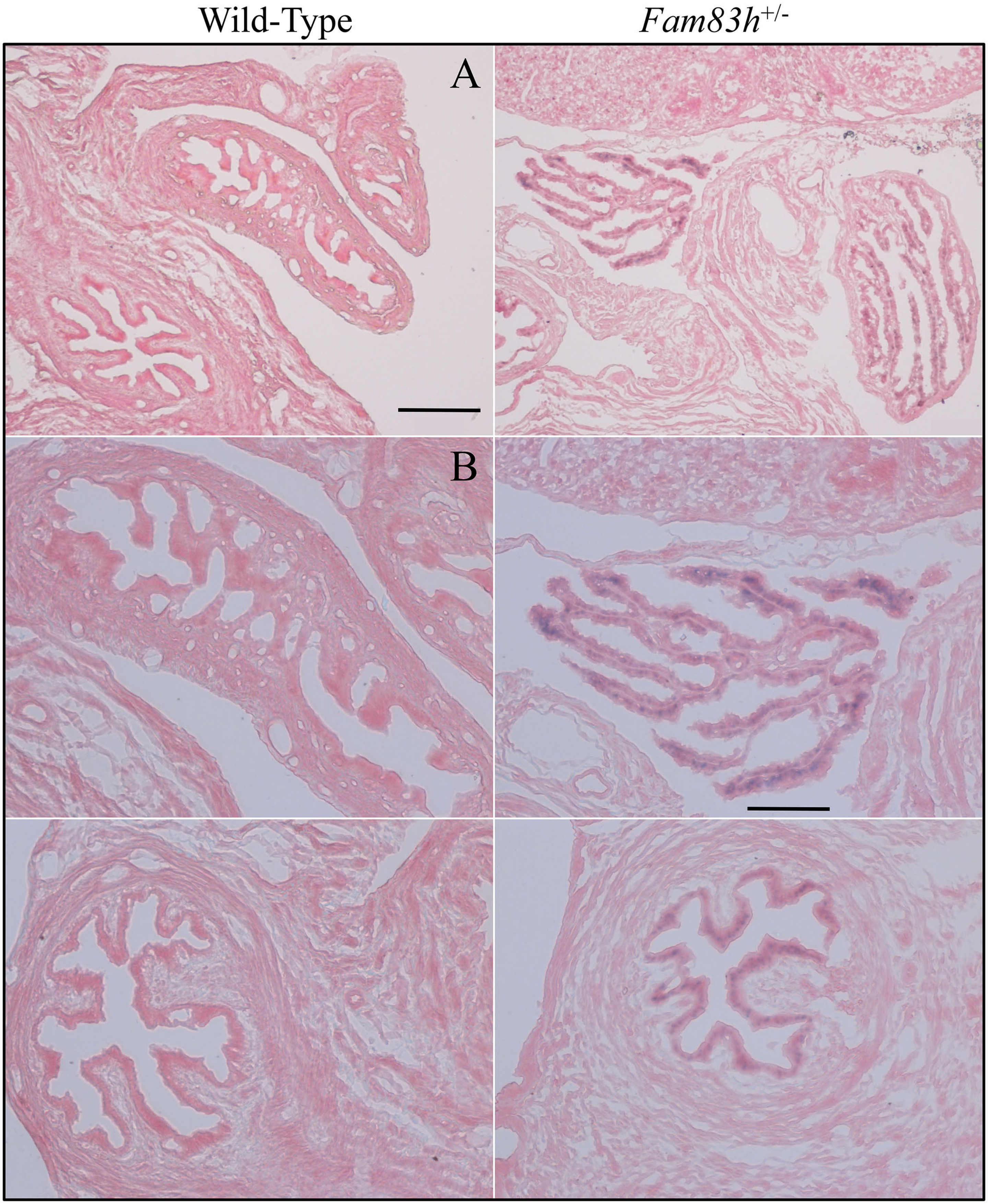

Fig. S24. *Lac*Z Histochemistry of Oviduct at 7-Weeks. Columnary epithelium reported positive. ***A:*** Scale bar 200 µm. ***B:*** Scale bar 100 µm.

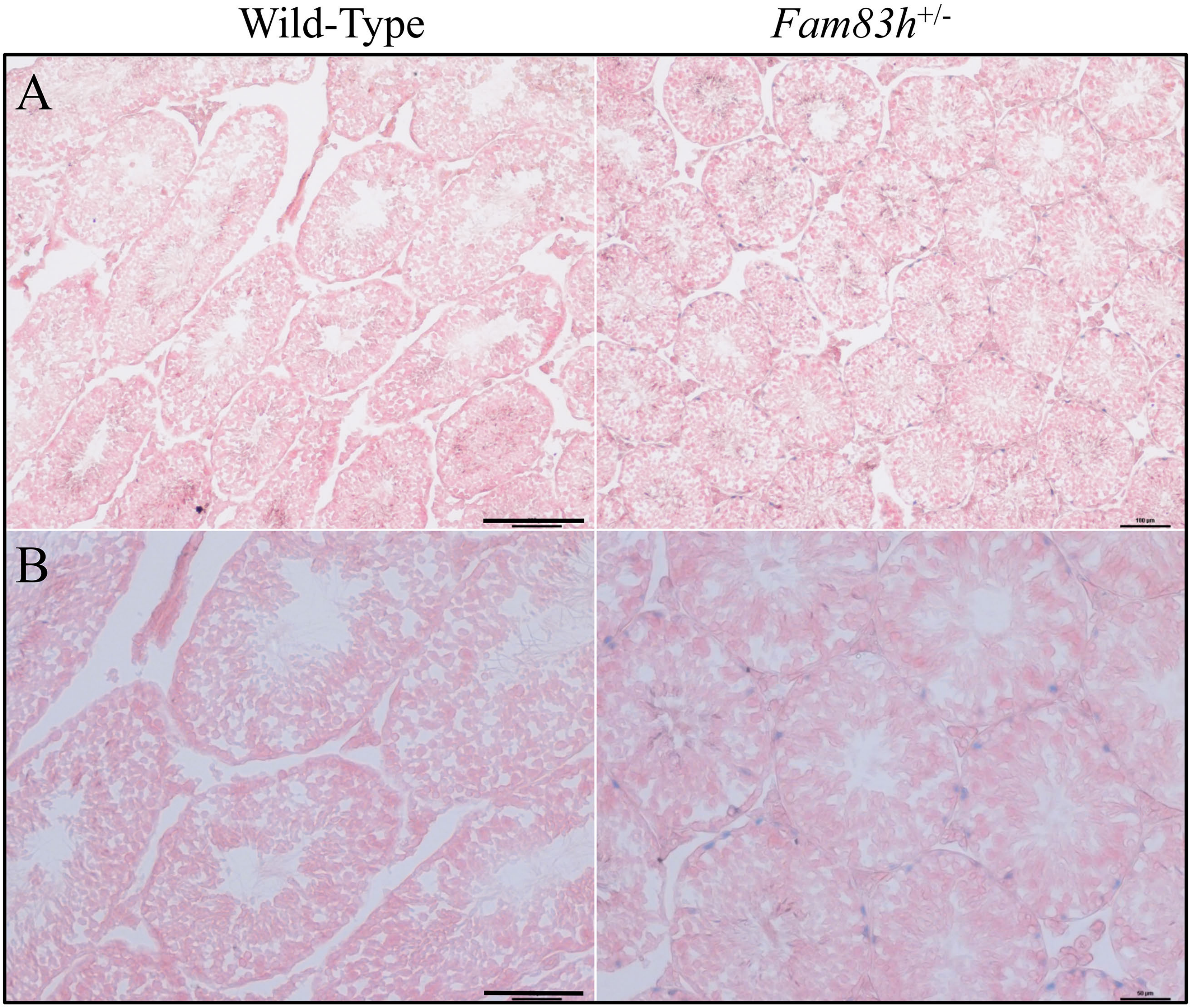

Fig. S25. *Lac*Z Histochemistry of Testis at 7-Weeks. Sertoli cells reported weakly postitive. ***A:*** Scale bar 200 µm. ***B:*** Scale bar 100 µm.

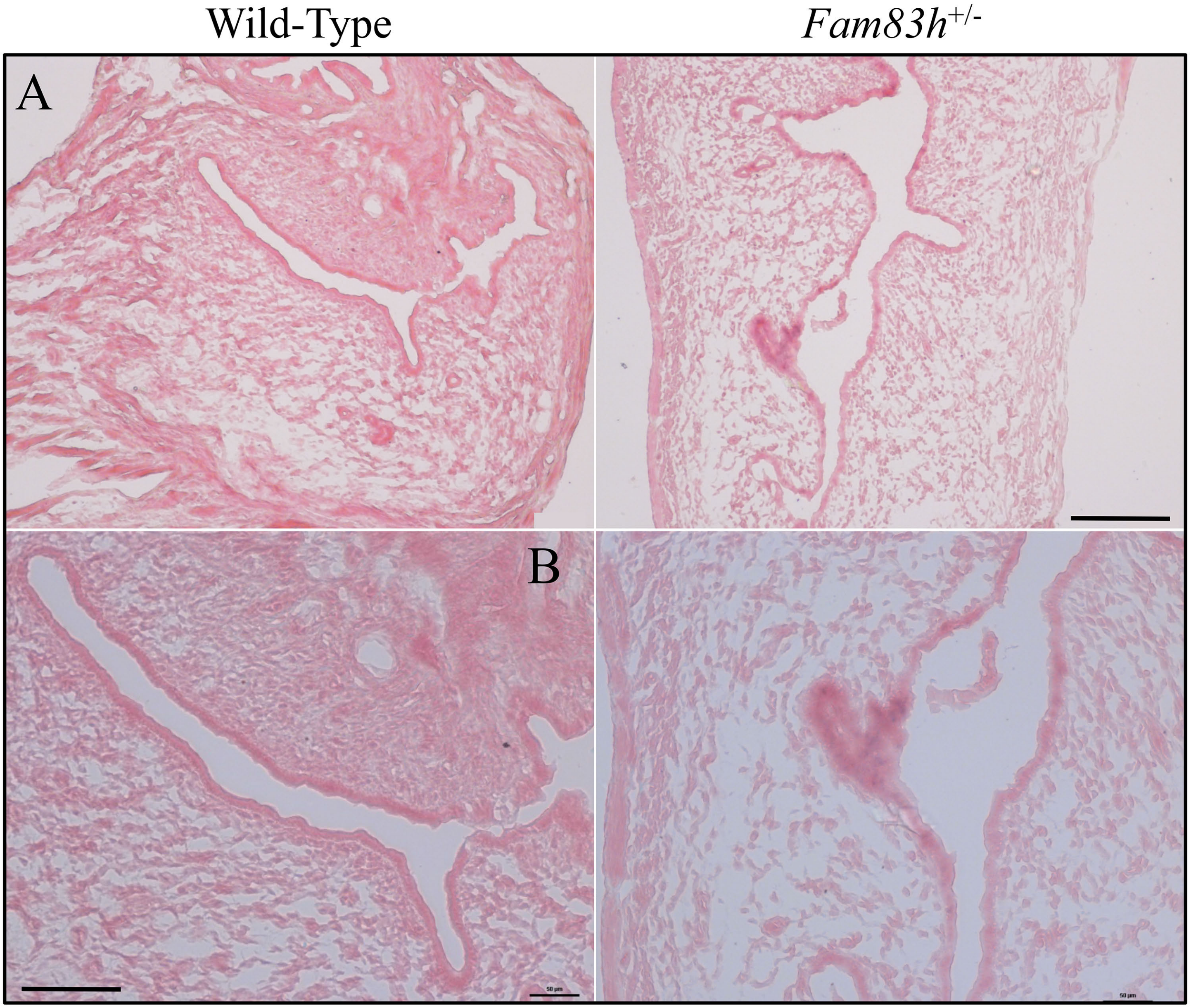

Fig. S26. *Lac*Z Histochemistry of Uterus at 7-Weeks. Trace expression was reported in columnar epithelium. ***A:*** Scale bar 200 µm. ***B:*** Scale bar 100 µm.

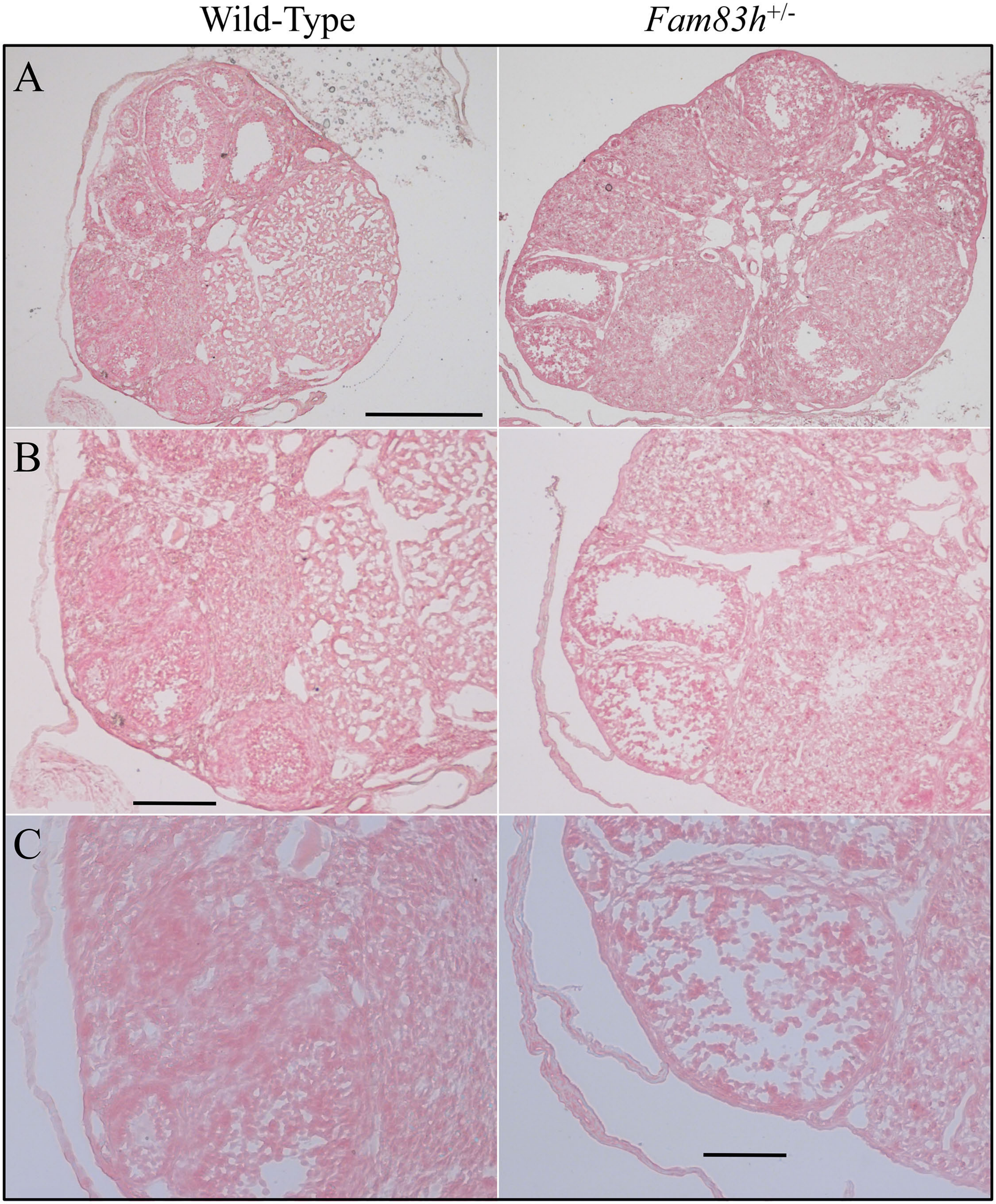

Fig. S27. *Lac*Z Histochemistry of Ovary at 7-Weeks. No staining. ***A:*** Scale bar 500 µm. ***B:*** Scale bar 200 µm. ***C:*** Scale bar 100 µm.

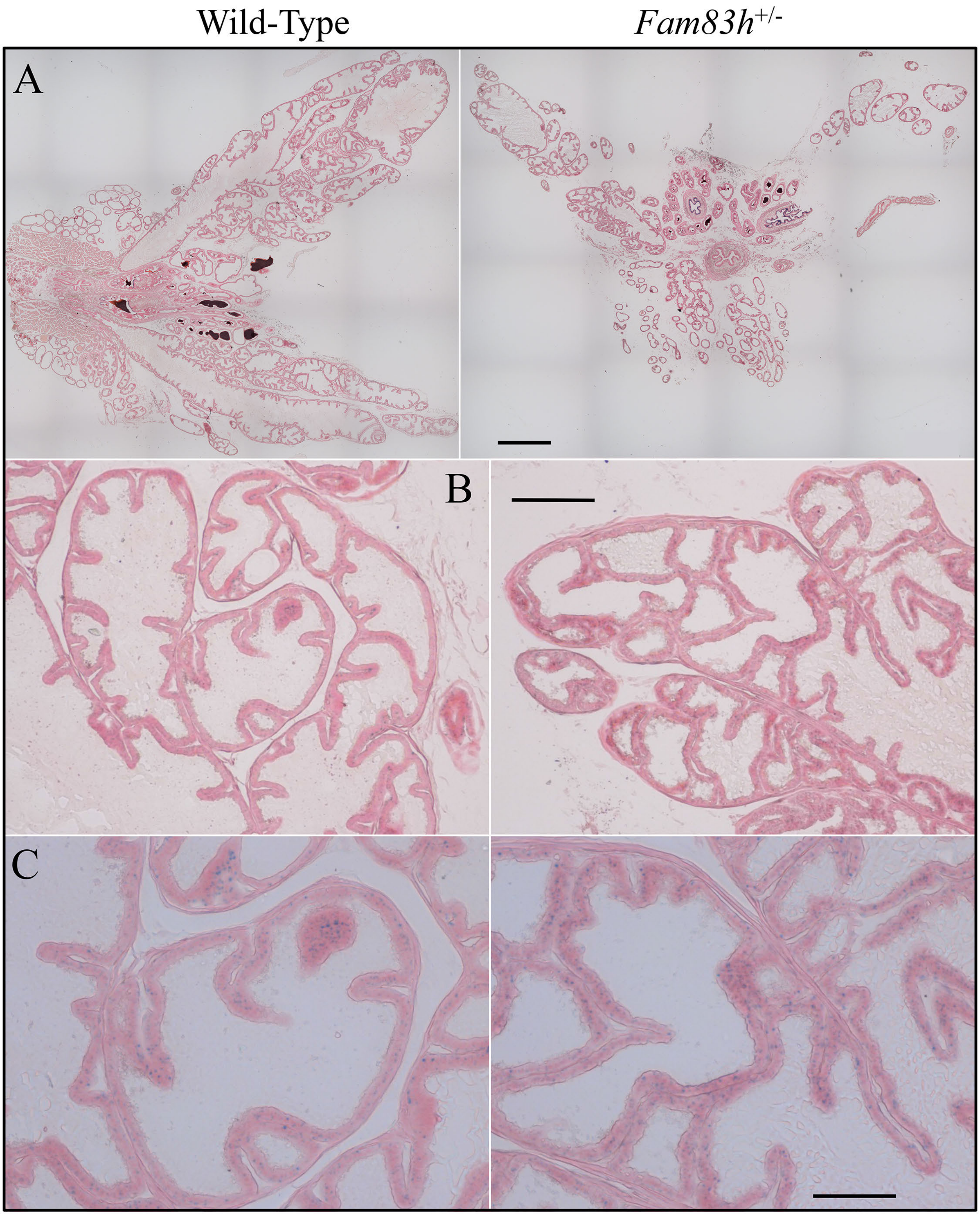

Fig. S28. *Lac*Z Histochemistry of Prostate at 7-Weeks (Part 1). The dorsal prostate cuboidal epithelium cells reported weakly positive in both the wild-type and *Fam83h* mice. ***A:*** Scale bar 1 mm. ***B:*** Scale bar 200 µm. ***C:*** Scale bar 100 µm.

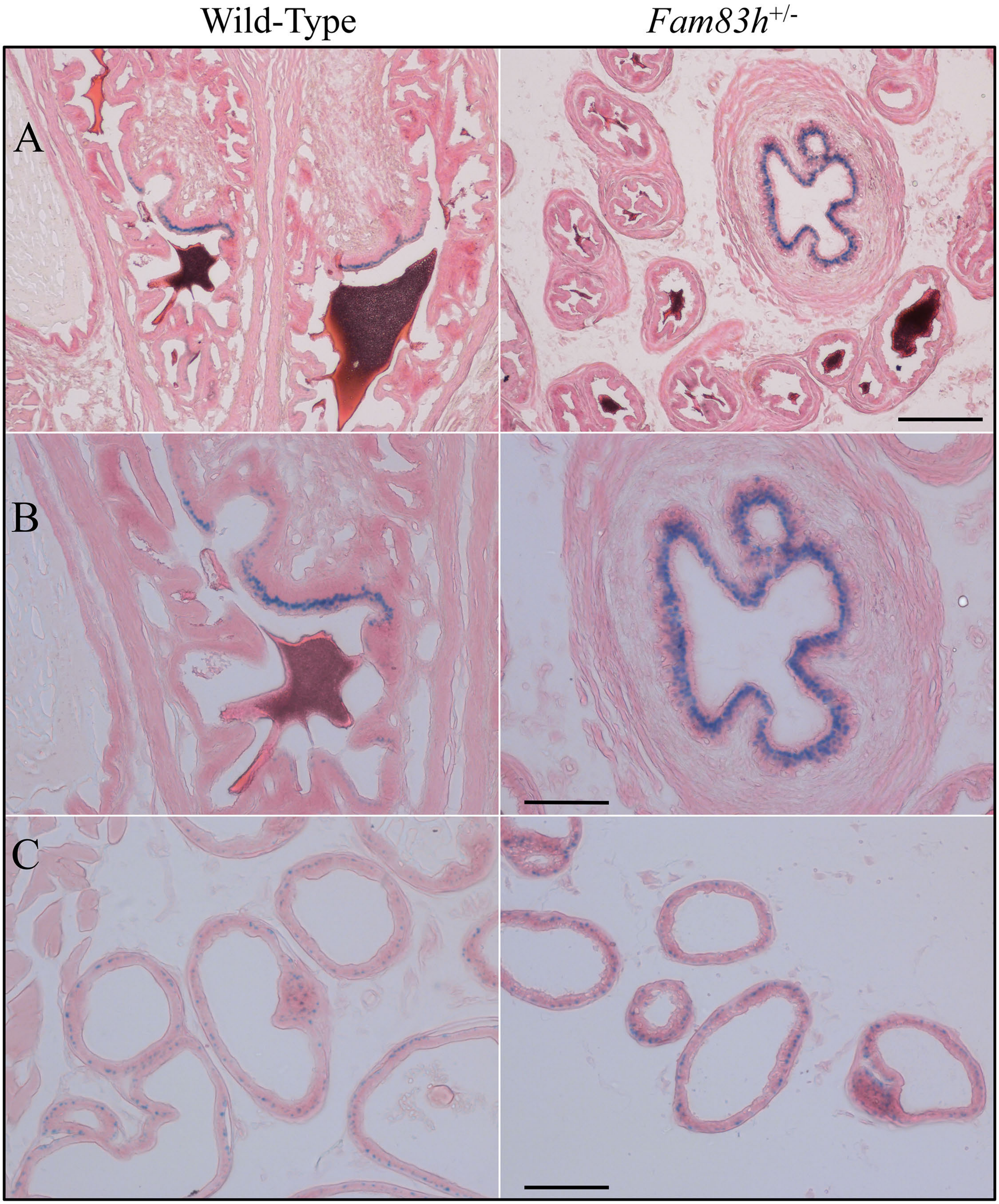

Fig. S29. *Lac*Z Histochemistry of 7 Week Prostate (Part 2). The dorsal prostate cuboidal epithelium reported positive in both the wild-type and *Fam83h* mice. ***A:*** Scale bar 200 µm. ***B:*** Scale bar 100 µm. ***C:*** Scale bar 100 µm.

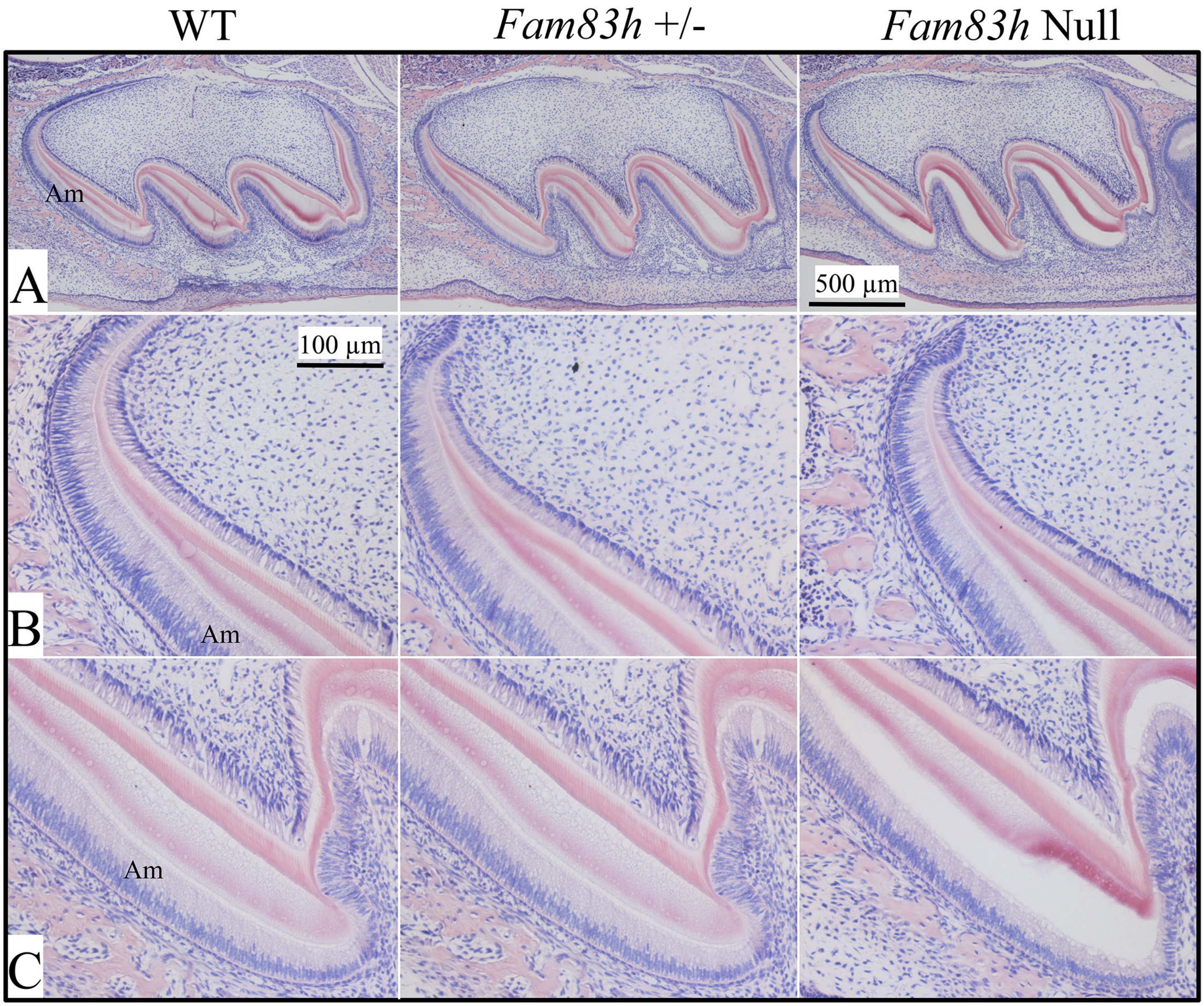

Fig. S30. Histology of Mouse Maxillary First Molars at PN5. Ameloblasts (Am) are in the secretory stage of amelogenesis. ***A:*** Low Magnification views. B: Higher magnification of youngest ameloblasts near the cervical loop. ***C:*** Higher magnification of the older ameloblasts on the cusp slopes and tip. No differences were observed among the ameloblasts from the 3 different genotypes.

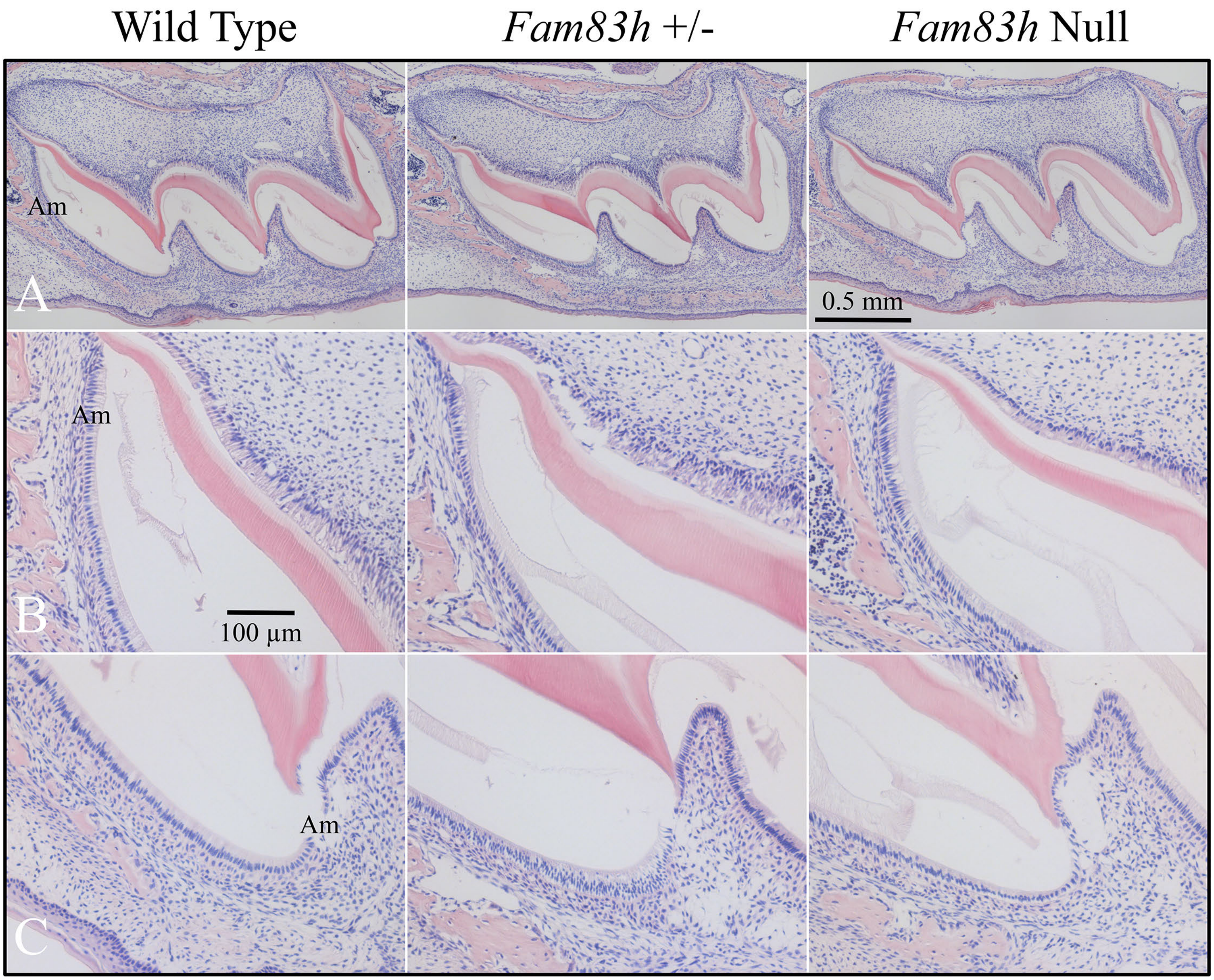

Fig. S31. Histology of Mouse Maxillary First Molars at PN11. Ameloblasts (Am) are in the maturation stage of amelogenesis. ***A:*** Low Magnification views. ***B:*** Higher magnification of youngest maturation stage ameloblasts near the cervical loop. ***C:*** Higher magnification of the older maturation stage ameloblasts on the cusp slopes and tip. No differences were observed among the maturation stage ameloblasts from the 3 different genotypes.

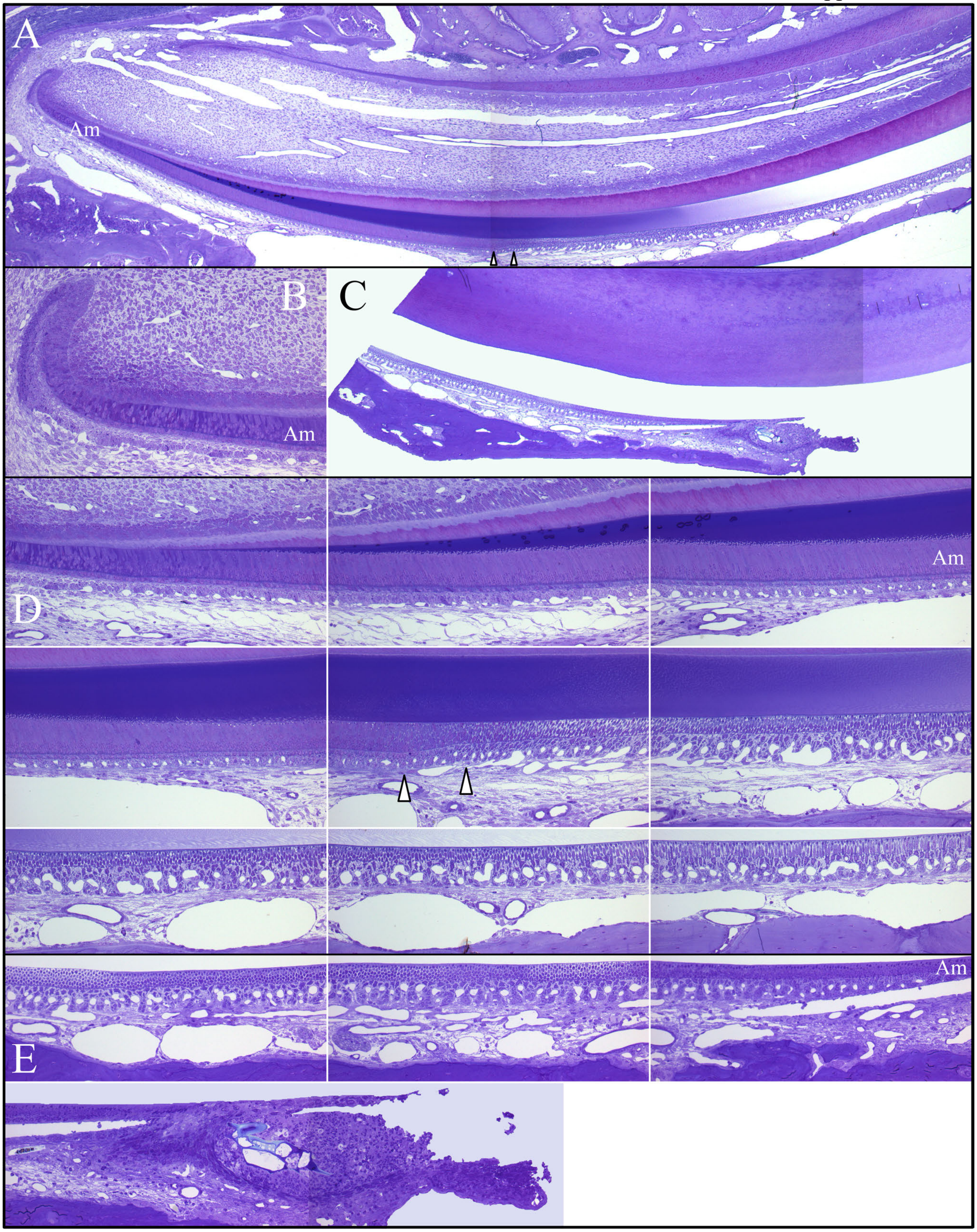

Fig. S32. Histology of *Fam83h* Null Mandibular Incisor at 7-weeks. ***A:*** Low magnification composite image of the mandibular incisor, block 1 and ***B:*** block 2. ***C:*** Basal end of incisor. ***D:*** Higher magnification views of ameloblasts (Am) from block 1 and ***E:*** block 2. Arrowheads mark the boundaries of the transition stage between the secretory and maturation stages of amelogenesis. Am, Ameloblasts.

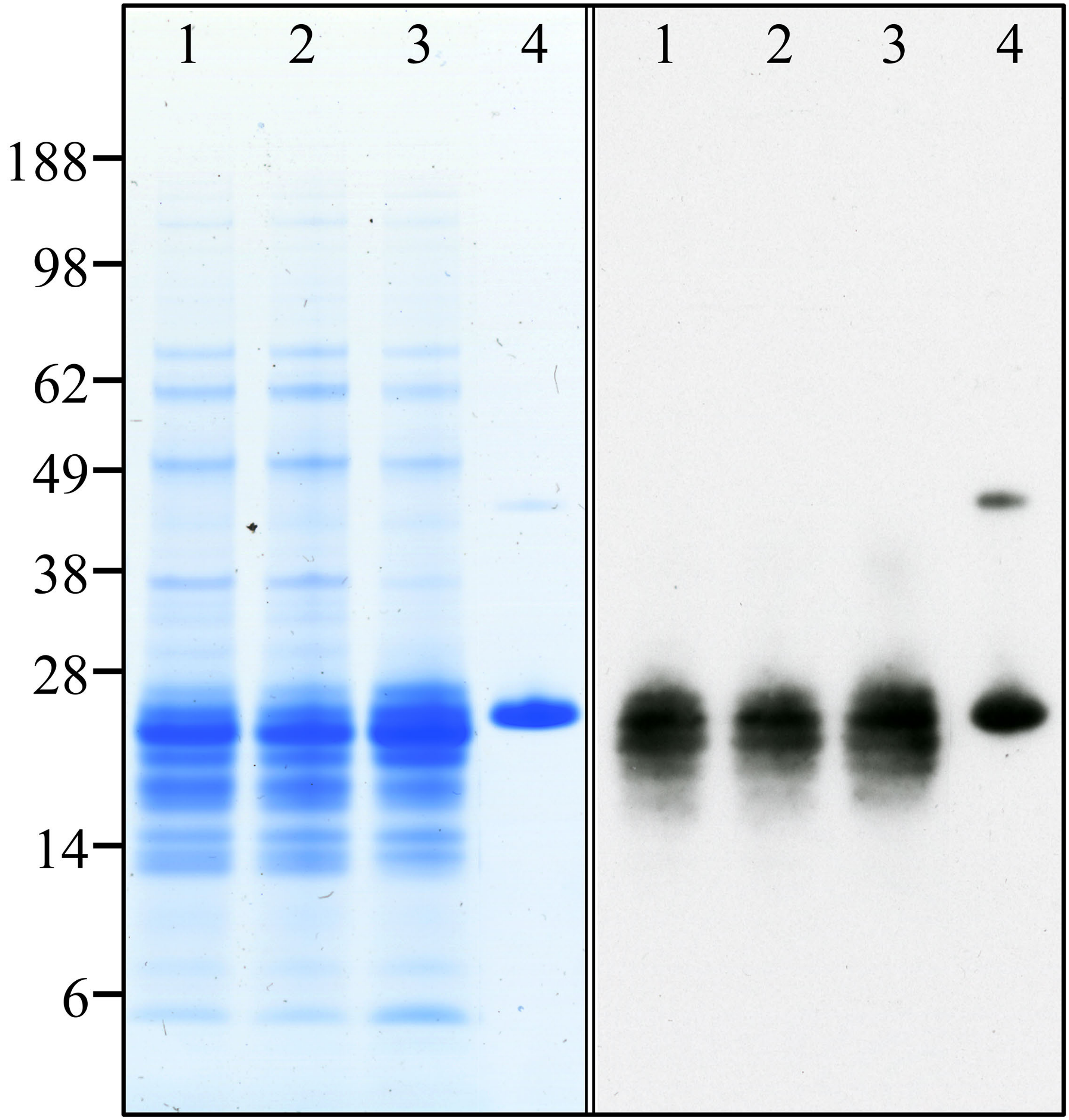

Fig. S33. SDS-PAGE and Western Blots of enamel proteins. ***Lane 1***: wild-type; ***Lane 2***: *Fam83h*$^{+/-}$; *Lane 3*: *Fam83h*$^{-/-}$; ***Lane 4***: rM179. Enamel proteins were extracted from D5 first molars. ~15% of the extracted proteins from a single molar was run down each lane and visualized by Coomassie Brilliant Blue staining. ~5% of a single molar extract was run down each lane and transblotted to a membrane and immunostained using an antibody raised against recombinant mouse amelogenin (rM179).

Protein Symbol	Protein Name	Protein Symbol	Protein Name	Protein Symbol	Protein Name
**ACTB**	actin, beta	**HNRNPA1**	heterogeneous nuclear ribonucleoprotein A1	**RCN1**	reticulocalbin 1, EF-hand calcium binding domain
**ACTG1**	actin, gamma 1	**HNRNPC**	heterogeneous nuclear ribonucleoprotein C (C1/C2)	**RCN2**	reticulocalbin 2, EF-hand calcium binding domain
**ACTR2**	ARP2 actin-related protein 2 homolog (yeast)	**HSPA1A**	heat shock 70kDa protein 1A	**RFC3**	replication factor C (activator 1) 3, 38kDa
**ARF4**	ADP-ribosylation factor 4	**HSPA9**	heat shock 70kDa protein 9 (mortalin)	**RPL22**	ribosomal protein L22
**ATAD3A**	ATPase family, AAA domain containing 3A	**IGF2BP1**	insulin-like growth factor 2 mRNA binding protein 1	**RPL27**	ribosomal protein L27
**ATAD3B**	ATPase family, AAA domain containing 3B	**KRT1**	keratin 1	**RPL28**	ribosomal protein L28
**CAD**	carbamoyl-phosphate synthetase 2, aspartate transcarbamylase, and dihydroorotase	**KRT10**	keratin 10	**RPLP0**	ribosomal protein, large, P0
**CALU**	calumenin	**KRT2**	keratin 2	**RPS13**	ribosomal protein S13
**CAPZA1**	capping protein (actin filament) muscle Z-line, alpha 1	**KRT9**	keratin 9	**RPS14**	ribosomal protein S14
**CKAP5**	cytoskeleton associated protein 5	**MYH10**	myosin, heavy chain 10, non-muscle	**RPS19**	ribosomal protein S19
**CNOT1**	CCR4-NOT transcription complex, subunit 1	**MYH14**	myosin, heavy chain 14	**RPS20**	ribosomal protein S20
**CSNK1A1**	casein kinase 1, alpha 1	**MYH9**	myosin, heavy chain 9, non-muscle	**RPS25**	ribosomal protein S25
**CSNK1E**	casein kinase 1, epsilon	**MYL6**	myosin, light chain 6, alkali, smooth muscle and non-muscle	**SEC16A**	SEC16 homolog A (S. cerevisiae)
**DDX3X**	DEAD (Asp-Glu-Ala-Asp) box polypeptide 3, X-linked	**NES**	nestin	**SLC25A13**	solute carrier family 25, member 13 (citrin)
**DNAJB2**	DnaJ (Hsp40) homolog, subfamily B, member 2	**NPM1**	nucleophosmin (nucleolar phosphoprotein B23, numatrin)	**SNRNP200**	small nuclear ribonucleoprotein 200-kDa (U5)
**DNAJB6**	DnaJ (Hsp40) homolog, subfamily B, member 6	**PCBP1**	poly(rC) binding protein 1	**SNRPD2**	small nuclear ribonucleoprotein D2 polypeptide 16.5kDa
**FAM83H**	family with sequence similarity 83, member H	**PPP1CA**	protein phosphatase 1, catalytic subunit, alpha isoform	**TIMM50**	translocase of inner mitochondrial membrane 50 homolog (S. cerevisiae)
**GAPDH**	glyceraldehyde-3-phosphate dehydrogenase	**RBM14**	RNA binding motif protein 14	**TOMM22**	translocase of outer mitochondrial membrane 22 homolog (yeast)
**HELZ**	helicase with zinc finger	**RBM4**	RNA binding motif protein 4	**TRAFD1**	TRAF-type zinc finger domain containing 1

Fig. S34. Proteins that immunoprecipitated with FAM83H.

```
Hum -------------------------------------------------------MARRS    5
Cow -------------------------------------------------------MARRS    5
Dog -------------------------------------------------------MARRS    5
Rat -------------------------------------------------------MARRS    5
Mos -------------------------------------------------------MARRS    5
Chk -------------------------------------------------------MARRS    5
Xep MGPNLPRASQRHRPRMGPNLPRASQWHRPRMGPICPGLLSGTGPVPPLAPLWCSPMARRS   60
Zef -------------------------------------------------------MARRS    5
                                                           *****

Hum QSSSQGDNPLAPGYLPPHYKEYYRLAVDALAEGGSEAYSRFLATEGAPDFLCPEELEHVS   65
Cow QSSSQGDNPLAPGYLPPHYKEYYRLAVDALAEGGPEAYSRFLASEGAPAFLCPEELEHVS   65
Dog QSSSQGDNPLAPGYLPPHYKEYYRLAVDALAEGGPEAYSRFLASEGAPAFLCPEELEHVS   65
Rat QSSSQGDNPLAPGYLPPHYKEYYRLAVDALTEGGQEAYNRFLASEGAPDFLCPEELEHVS   65
Mos QSSSQGDNPLAPGYLPPHYKEYYRLAVDALTEGGPEAYNRFLASEGAPDFLCPEELEHVS   65
Chk QSSSQGDNPLDPNYLPPHYKEYYRLALDILTEEGKESYERFLAEEGAPDFLCNSEVDHIL   65
Xep QSSSQGENPLDPNYLPPHYKEYYRIAIDALAENGPEAYEQFLMEEGAPDFLCPNEVEHIS  120
Zef QSSSLGDNPLDPNYLRPHYREEYRMAIDALVEDDIEGYYNFLQNANVVDFLSRSEIENIK   65
    **** * *** * ** *** * ** * * * *   * *  **       **   *

Hum RHLRPPQYVTREPP---EGSLLDVDMDGSSGTYWPVNSDQAVPELDLGWPLTFG--FQGT  120
Cow RHLRPPQHVAPEPP---DGSPPNLDFDGSSGTYWPVNSDQAVPELDLGWPLTFG--FQGT  120
Dog RHLRPPQHVAREPP---DGSPPNVDMDGSSGTYWPMNSDQAVPELDLGWPLTFG--FQGT  120
Rat RHLQPPQYVSREPP---EGAPPDVDMDGSSGTYWPVDSDQAVPELDLGWPLTFG--FQGT  120
Mos RHLQPPQYVAREPP---EGTPSDVDMDGSSGTYWPVNSDQAVPELDLGWPLTFG--FQGT  120
Chk QNLQKPQYANQEG---GTDTAGDNDVDGSSGTYWPMNSDLAVPELDLGWPMVFG--FRGT  120
Xep RSLQRPPESGQENPYPDSVYGSQEDADGSSGTYWPMDSDTAAPELDLGWPTIYG--FQGT  178
Zef STVQTPQSAGNVP----ELPYGEIDQDESSDTYWPLHSDLDAPGLDLGWPMQQHSFVGPT  121
         *                  * * ** ****  **   * ******         *

Hum EVTTLVQPPPPDSPSIKDEARRMIRSAQQVVAVVMDMFTDVDLLSEVLEAAARRVPVYIL  180
Cow EVTTLVQPPPPDSPSIKDEARRMIRSAQQVVAVVMDMFTDVDLLSEVLEAAARRVPVYIL  180
Dog EVTTLVQPPPPDSPSIKDEARRMIRSAQQVVAVVMDMFTDVDLLGEVLEAAARRVPVYIL  180
Rat EVTTLVQPPPPDSPSIKDEARRMIRSAQQVVAVVMDMFTDVDLLSEVLEAAARRVPVYIL  180
Mos EVTTLVQPPPPDSPSIKDEARRMIRSAQQVVAVVMDMFTDVDLLSEVLEAAARRVPVYIL  180
Chk EVTTLVQPPPPDNPSIKEEARRMIRAAQQVVAIVMDVFTDVDLLFEVLDAAARRVPVYIL  180
Xep EVTTLMHPPPPDNPTIKEEVRRMIRSAQQVIGIVMDIFTDADILSELLDAANRRIPVYII  238
Zef EVTMLVNPAEPERPSIKEQARRLIKNAHQVIAVVMDIFTDVDIFSDLLEAAARHVPVYIL  181
    *** *  *  *  * **   ** *  * **   *** *** *     * ** *  ****

Hum LDEMNAQHFLDMADKCRVNLQHVDFLRVRTVAGPTYYCRTGKSFKGHVKEKFLLVDCAVV  240
Cow LDEMNAQHFLDMADKCRVNLHHVDFLRVRTVAGPTYYCRTGKSFKGHVKEKFLLVDCAVV  240
Dog LDEMNAQHFLDMADKCRVNLHHVDFLRVRTVAGPTYYCRTGKSFKGHVKEKFLLVDCAVV  240
Rat LDEMNAQHFLDMADKCRVNLHHVDFLRVRTVAGPTYYCRTGKSFKGHLKEKFLLVDCAVV  240
Mos LDEMNAQHFLDMADKCRVNLHHVDFLRVRTVAGPTYYCRTGKSFKGHLKEKFLLVDCAVV  240
Chk LDEMNSQLFLDTAAKCRVNLNYVEFLRVRTVSGPTYYCRTGMSFKGHVKEKFLLVDCMVV  240
Xep LDQMNCQLFLDMAAKYRVNLNYVEFLRVRTVSGPTYFCRKGSTFKGNLQEKFLLVDCTMV  298
Zef LDEQNAHYFVNMVASCKVNLEMIHMMRVRTVSGVTYFCRTGKSFKGQVMDRFLLTDCRAV  241
    **  *   *        ***      ***** * ** ** *  ***     *** **  *
```

```
Hum MSGSYSFMWSFEKIHRSLAHVFQGELVSSFDEEFRILFAQSEPLVPSAAALARMDAYALA  300
Cow MSGSYSFMWSFEKIHRSLAHVFQGELVSSFDEEFRILFAQSEPLVPSAGALARMDTYALA  300
Dog MSGSYSFMWSFEKIHRSLAHVFQGELVSSFDEEFRILFAQSEPLVPSAGALARMDAYTLA  300
Rat MSGSYSFMWSFEKIHRSLAHVFQGELVSSFDEEFRILFAQSEPLVPSAGALARMDAYALT  300
Mos MSGSYSFMWSFEKIHRSLAHVFQGELVSSFDEEFRILFAQSEPLVPSAGALARMDAYALA  300
Chk LSGNYSFMWSFEKIHRSIAHIFQGELVASFDEEFRILFAQSEPLVPPANVLAKAE----N  296
Xep LSGTYSFMWSFEKIHRSIAHIFQGELVSSFDEEFRILFAQSDPLIPSESALAKMD----K  354
Zef ISGNYSFMWSFEKIHRCIAHLFLGELVATFDEEFRILFAQSQPLVVENALVPMPQ-----  296
     ** ************  ** * ****  ************ **

Hum PYAGAGPLVGVPGVGAPTPFSFPKRAHLLFPPPREEGLGFPSF-LDPDRHFLSAFRREEP  359
Cow PYAGAGPLMGSQMPGAPTPFSFPKRAHLLFPPPREEGLGFPSF-LDPDRHFLSAFRREES  359
Dog PYTGAGPLMG-----APTPFSFPKRAHLLFPPPREEGLGFPSF-LDPDRHFLSAFRREEP  354
Rat PYSGAGPLVGVPGVGAPTPFSFPKRAHLLFPPPREEGLGFPSF-LDPDRHFLSAFRRDEL  359
Mos PYSGAGPLVGVPGVGAPTPFSFPKRAHLLFPPPREEGLGFPSF-LDPDRHFLSAFRREEL  359
Chk PFA-MTPFGN------NMPF-FPKKSPLMFQRDDNLFPSFMDR-VDPDRFFLSNFRRDDM  347
Xep SYMGMVPFAG------PRPM-FDRKLHFMFPREENPSQQFPSYGVDPDRHYFQPFRREDM  407
Zef -----DSYLG-------NQFGLKRTQSLRNPRGYLRQPELGGYQYGDRLDSILPFRRDDP  344
                                                          ***

Hum PRMPGGALEPHAGLRPLSRRLEAEAGPAGELAGARGFFQARHLE-MDAFKRHSFAT-EGA  417
Cow SRMPGGALEPHTGLRPLSRRLDAEAGPGGELSGPRGFFQARHLE-MDAFKRYSYAAADGA  418
Dog MRMPGGALEPHAGLRPLARRLDAEAGAGGELAGPRAFFQARHLE-MDAFKRHSFAAADGS  413
Rat QRMPGGALEPHTGLRPLAR--PGEAGPLGELPGPRGFFQSRHLE-MDAFKRHSYTAADGA  416
Mos QRMPGGALEPHTGLRPLAR--PTEAGPFGELAGPRGFFQSRHLE-MDAFKRHSYATPDGA  416
Chk LRHT---VEGSA-----MRMYKKVEMENAQMDPVRGFLRSKQLE-LDAFKRHSFAE----  394
Xep IRQT---MDPGG-----MRMYGKNLGDPMDKMQMS-FVQNKQLEAMEAFKRHSFAE----  454
Zef FRHT---IEPSAGPMQVTKYATQQFRMQQSFLDQGRSMLASRQLEMNAFKRHSYAE----  397
     *                                             **** *

Hum GAVENFAAARQVSRQTFLSHGDDFRFQTSHFHRDQLYQQQYQWDPQLTPARPQGLFEKLR  477
Cow GAVENLAAARQVSRQTFLSHGDDLRFQTSHFHRDQLYQQHYQWDPQLAPTRPQGLFEKLR  478
Dog GAVENFAAARQVSRQTFLSHGDDLRFQTSHFQRDQLYQQHYQWEPQLAPARPQGLFEKLR  473
Rat GAVENFAAARQVSRQTFLSHGDDFRFQTSHFQRDQLYQQHYQWDPQFAPARPQGLFEKLR  476
Mos GAVENFAAARQVSRQTFLSHGDDFRFQTSHFQRDQLYQQHYQWDPQFAPARPQGLFEKLR  476
Chk GTFENFASSKQYARQMFMNNMDEFKIQSSHFQKDQFYQY--QFEHPHLSGRPQGFFDRIR  452
Xep GTFENYTSSRQYSRQMFMNNNDEYRLQSSQVQKSQFMQ----FQSPLGTARPQGLFEKIR  510
Zef GTRETYASSRQYMKQRVMNN---LEETESHYQREQHYYQ-----SEGMHDDRGHYDRFN  449
    *  *      *   *             *     *                  *

Hum GGRAGFADPDD---FTLGAG----PRFPELGPDG-HQRLDYVPSSASREVRHGSDPAFAP  529
Cow AGRPGFPDHDE---FMLGTG----PRFPELGLDG-HQRLDYVPSSASREVRHGSDPAFGP  530
Dog AGRPGFTEHDD---LALGLG----PRFPELGPDG-HQRLDYVPSSASREVRHGSDPAPGP  525
Rat AGRPGFADPDD---FALGAG----HRFPELGADV-HQRLEYVPSSASREVRHGSDPAFGP  528
Mos AGRPGFADPDD---FALGAG----HRFPELGADV-HQRLEYVPSSASREVRHGSDPAFGP  528
Chk GGRPGFNELEEGGPLPYGEGPRYHELESGFPQEGFPLRLDYVPSNSSREVRHGSD-QLNP  511
Xep GGRQGLQEMDEFDSRYPTKG---LPGEGHFALDGPPMRPGYNPSNSSREVRHGSD-QMVI  566
Zef YGLADQHSDSG--------------YPPELEAPG------NINVLSSDDLKSDSEKQYNI  489
     *                                            *      *
```

```
Hum GPRGLEPSGAPRPNLTQRFPCQAAARPGPDPAP--------EAEPERRGGPEGRAGLRRW  581
Cow GPRGLEPGGAPRPNLGQRFSCQAMTRLGPEMAP--------EPEPEHRGGPEGRAGLRHW  582
Dog APRGLEPSGALRANLGQRFPCQAAARPGPEAAP--------DTEQERRGGPEGRAGLRHW  577
Rat SPRGLEPSGASRPNLGQRFPCQATLRQGLDTAT--------EAEPERRGGPEGRAGLRHW  580
Mos SPRGLEPSGASRPNLGQRFPCQATLRQGLDTAS--------EAEPERRGGPEGRAGLRHW  580
Chk AGNGPMGMMLRRQNIGQKFICQTSPTQKQSLEQRLFLQDKDEDQDQDKSTQENRTGLRNW  571
Xep GGEGRFGQRSLGR---QKFMCQISPTQKQGMEPKYFFHD----QDADKKPQENKQGLRSW  619
Zef GGR-YDPQGHKRPAAGHAYACQSSPTQPHPPDQKQLFSTG--DQVRQSQDPSVKQGLRSW  546
                        **                                 *** *

Hum RLASYLSGCHGEDGGDDGLPAPMEAEAYEDDVLA--------------------------  615
Cow RLTSYLSGCHSEDAGDEGLPTPMETEAYDDDVLV--------------------------  616
Dog RLASYLSGCHGDDAGDEGLPAPMDAEAYEDDVLG--------------------------  611
Rat RLASYLSGCHG-DGGEEGLP--MEAEACEDEVLA--------------------------  611
Mos RLASYLSGCHG-DGGEEGLP--MEAEACEDEVLA--------------------------  611
Chk RISSYLSAYQS-EP-EEGLPMPMESEAYNDVLG---------------------------  602
Xep RISSYLSGIQS-DQDEEGLPIPLDPELYDDALVPVERAVPASDTLFKYSMDPVPPYHPGT  678
Zef RINSYLSTYED--GGEEGLHQPMGSDAFEDSHQQPDSRLYGSEGPGIHSN----------  594
    *  ****          **          *

Hum -------------------------------------------PGGRAPAGDLLPSAFRV  632
Cow -------------------------------------------SGGRATAGDLLPSAFRV  633
Dog -------------------------------------------CAGRPSPGDLLPSATRV  628
Rat -------------------------------------------PGGR----DLLPSTFRT  624
Mos -------------------------------------------PGGR----DLLPSAFRT  624
Chk -------------------------------------------DPLTKHPTDLIPAFKSP  619
Xep APHDLPYDRANENPMKFSMDPVQLHRPNVPSQDVPMHLERAGNESLVKYSLDPIPPFKPN  738
Zef --------------------------------------IRERPNIPTKPNLDLRPRFGKP  616
                                                       *  *

Hum PAA------------------------------FPTKVPVPGPGSGG-NGPEREGPEEPG  661
Cow PAP------------------------------FPGKGPAPGSGSGGGDGPEREGLEEVG  663
Dog PAA------------------------------FPAKGLEPCSGR--GDSPERETLEEAG  656
Rat PAP------------------------------FPAKGPKPGSGSGGGDSSEREGPEETG  654
Mos PAA------------------------------FPAKGPKPGSGSGGGDSSEREGPEETS  654
Chk IS-------------------------------FNSK-SLGVENAKEFADPERGG-EEAP  646
Xep VAGTDVPMPLERKPTANEILSRYSVDPIPPYKTFGSTGDLSVEKAKENPPAEKEK-EEGL  797
Zef IIQDRNQVKDNTSDLG-----------PTSTDTLKPAISASSLASSTDNEKELAEPREIS  665
                                                       *     *

Hum LAKQDSFRSRLNPLVQRSSRLRSSLIFSTSQAEGAAG-AAAATEKVQLLHKEQ--TVSET  718
Cow LAKQDSFRSRLNPLIQRSSRLRSSLIFSASQGEGTGGPTGASTEKVQLLHKEQ--TVSEM  721
Dog LAKQDSFRSRLNPLIQRSSRLRSSLIFSASPAEGAGGAPAAATERAQLLHKEQ--AVSET  714
Rat LIKQDSFRSRLNPLIQRSSRLRSSLIFAS-QAEGTAGTTAATTEKVQLMHKEQ--TISET  711
Mos LAKQDSFRSRLNPLIQRSSRLRSSLIFAS-QAEGAVGTAAATTEKVQLMHKEQ--TVSET  711
Chk MMKQDAFRTRINPLIQRSSRLRSSLIFNA----AKLDQPNTTVEKVQMIHKEQ--VSSEL  700
Xep LSRHDSFRTRTNPLIQRGSRLRSSLIFSS----SKLEQHTSTAESVQEMQKEQ--STSEL  851
Zef ITKHESFRTRINPMLQRSSRLRSSLIFSSSKLEQHNSSQAKSGGELQEEKEESEPIRYSS  725
          ** * **  ** *********                   *    *
```

```
Hum LGPGGEAVRSAASTKVAELLEKYKGPARDPGGGAGAITVASHSKAVVSQAWREEVAAPGA  778
Cow LGPGNEAVRSAASTKVAELLEKYKGPSRDPGGVGTAVTAASHSKAVVSQAWREEVSAPGG  781
Dog LGPGGEATRSATSTKVAELLEKYKGPARDAGAVGAAVAVASHSKAVVSQAWREEVVAPGG  774
Rat LGPSGEAVRSSASAKVAELLEKYKGPARDPGGAGGAITASSHSKAVVSQAWREEVVAPGG  771
Mos LGPSGEAVRSSASAKVAELLEKYKGPARDPGGAGGAVTSSSHSKAVVSQAWREEVVAPGG  771
Chk TK-DNETIKTAASSKVAELLEKYKAVGKDAER-----ATVTHTKAVSSFLQEESQNAEKK  754
Xep VS-ENETGRTTS--KVAEILQKYRGINKDANS-----TTVTQAKAASRTIHEESEDGQSV  903
Zef IVAEILEKRRSLSREPFDWNKHKKADEKDVKHASTGDLTTIQDTKEEPIKEKEKPDNPKP  785
                                *                       *

Hum VG--GERRSLESCLLDLRDSFAQQLHQEAERQPGAASLT--AAQLLDTLGRS--------  826
Cow GSG-SERRSLESCLLDLRESFAQQLHQEAERQPGAATLA--STQLLDTLGRGS-------  831
Dog G---SERRSLESCLLDLRDSFAQRLHQEAERQPGAATLT--ATQLLDTLGRG--------  821
Rat AG--AERRSLESCLLDLRDSFAQQLHQEAERHPGAASLT--AAQLLDTLG----------  817
Mos AG--TERRSLESCLLDLRDSFAQQLHQEAERHPGAASLT--AAQLLDTLG----------  817
Chk CTKSVQYKILESRVLESKDSCSTYK--------------MHGETDRTFGMS--------  791
Xep SAEEVAYKAVES-TVDTKGSMSHVQQESQYRSVASSHLESLLGKHQTTLSMSKVEQMTSS  962
Zef EENKVTQPTVPSASQQITSSLNMNDPASRLQYFKDQQEKRKTSKLELDLGTKSQEAAIKK  845
               *       *

Hum -------------GSDRLPSRFLSAQSHSTSPQGLDSPLPLEGSGAHQVLHNESKGSPTS  873
Cow ------------SGTDRLPSRFLSTQGHSTSPQGRDSPPP---EGPG-GQQSEPKGSPTL  875
Dog -------------GTDRLPSRFLSAQGRPGSPQGRDSPPP---EGPRQAPHPEPKGSPAS  865
Rat -------------GTDRLPSRFLSAQGRSLSPQGRDSPPP-EGLGTHQLPYSEPKGSPTP  863
Mos -------------GTDRLPSRFLSAQGRSLSPQGRDSPPP-EGLGTHQLPYSEPKGNPTP  863
Chk ---------------SSTPQLIDALSKDPLAHLG----------TKVDKLSSRFYPMENK  826
Xep IQTIGNISAAPSESGPTVPELSEVHKQSSISHMQQESHYKSVVTSKLEGLLNRDQQVMSM 1022
Zef --------PETLDTATKVPDVLLTSEQSTVKAQEPTVSQTDPVPHRPVIETKPKPSEVSV  897
                      *

Hum AYPERKGSPTP-------------------------------------------GFSTR  889
Cow AYPERKGSPTP-------------------------------------------GFPTR  891
Dog AYPERKGS----------------------------------------------------  873
Rat AYPERKGSPTPAYPERKGSPTPAYPERKGSPTPAYPERKGSPTPAYPERKGSPTSGFPNR  923
Mos AYPERKGSPTPAYPERKGSPTPAYPERKGSPTPAYPERKGSPTQAYPERKGSPTSGFPNR  923
Chk PALPEKESLIF--------------------------------------------VGDT  841
Xep SKVEQTSSTIQ--------------------------------------------TIGN 1037
Zef DRPYTTNKTLTESIADAP-------------------------------KKEPVKEPTKS  926

Hum RGSPTTGFIEQKGSPTSAYPERRGSPVPPVPERRSSPVPPVPERRGSLTLTISGESPKA-  948
Cow RGSPTAGFTEQKGSPTSAYPERRGSPVPPVPERRGSPVPPVPERRGSLTLPFSGESPKA-  950
Dog --------------PTSAFPERRASPVPPVPERRASPVPPVPERRASLTLTFAEESAKT-  918
Rat RGSPTTGLMEQKGSPTSTYPDRRSSPVPPVPERRGSPVPPVPERRG--SLTFAGESSKT-  980
Mos RGSPTTGLMEQKGSPTSTYPDRRGSPVPPVPERRGSPVPPVPERRG--SLTFAGESSKT-  980
Chk QKLALPEKKER-VTFKEDAEKLVSAELK--KPQVRTGATSAIENLSKGQGSDSSLNRSE-  897
Xep ISPAPPDSKESGPTITEVTEATQSSENLPTRPNSAFHFGSALESMSQNPTPSSSLNKSE- 1096
Zef LKPFPSPKFLKPFKSSQSSSRRISCGEEILTDATDAEKSELKKSRSFSTSGMSRTESRES  986
```

```
Hum ----GPAEEGPSGPMEVLRKGSLRLRQLLSPKGERRMEDEGGFPVPQENGQPESPRRLSL 1004
Cow ----GPTEEAVGGPMEVLRKGSARLRQLMSPKGERRADDEGSFPTPQENGQPESPQWPSL 1006
Dog ----GTAEESAGGPMEVLRKGSLRLRQLLSPKGERRAEEDGGFPAPQENGQPESPRRPSL  974
Rat ----GPTEEVSGGPMEVLRKGSLRLRQLLSPKSERRGEDEVSFPAPQENGQPESPRRPSL 1036
Mos ----GPTEEVSSGPMEVLRKGSLRLRQLLSPKNERRGEDEGSFPTPQENGQPESPRRPSL 1036
Chk ----EECSKQEQNTMEFLRKGSLRLKQFLNPKGEKKLEEEPNSEIGKSDKQPMGLKRSSM  953
Xep ----EDLAKTDQN---FFRKGSMRLKQFLQSKAEKKAEEDLASDNAKAEKQHSTLRRLSK 1149
Zef LSSLGNSESKDTKALDFLKKQTQRLKGILGPKGDKKHSGVSNSQEDKSMKTVPEVQEEIS 1046
                       *   **      *

Hum GQGDSTEAATE------------------ERGPRARLSSATANALYSSNLRDDTKAILEQ 1046
Cow VRVDSTEAAAE------------------ERGPRARVASATANALYSSNLRDDTKAILEQ 1048
Dog GRADSTEAAAAA----------------DERGPRARTASATANALYSSNLRDDTKAILEQ 1018
Rat SRGDSTEAAAE------------------ERGSRVRLASATANALYSSNLRDDTKAILEQ 1078
Mos SRGDSTEAAAE------------------ERGSRVRLASATANALYSSNLRDDTKAILEQ 1078
Chk G--DCQEMLGE---EEKNHKFATLLPPKSSQPTQGRFPSSTANILYSSNLRDDTKVILEQ 1008
Xep S--DSQEVAASTDMEEKSAKSLSVSPPKTSSISQSRLSASTSNVIFSSNLRDDTKVILEQ 1207
Zef DKGKPSESISSS---------TAVENKPSAKPTTSRYQSSTSNIIFSSNLRDDTKVILEQ 1097
          *                            *    * *   ********* ****

Hum ISAHGQKHRAVPAPSPGPTHNSPELGRPPAAGVLAPDMSDKDKCSAIFRSDSLGTQGRLS 1106
Cow ISAHGQKHRGVPAPGPTPAHSSPELGHSPVAGGLAPDMSDKDKCSAIFRSDSLGTQGRLS 1108
Dog ISAHGQKHRAVPAPAPGLAHSSPELGRSPTAGGLAPDMSDKDKCSAIFRSDSLGTQGRLS 1078
Rat ISAHGQKHRAVPAP--GPAHNSSDVGRPTTAGDLAPDMSDKDKCSAIFRSDSLGTQGRLS 1136
Mos ISAHGQKHRGVPAP--GPAHSSPDVGRPTTAGDLAPDMSDKDKCSAIFRSDSLGTQGRLS 1136
Chk ISANSQKNRAELAKQLPSTSNP-DLSKSTMSLERKTE---KEKSCNIHRSESFGSQKRNL 1064
Xep ISANSQKNRAEMVKQAQQIQATGDPDPATSKPESKTEGTASTDAAAITRTGSFLSRSRFS 1267
Zef ISANSQKTRQQNEESGKGDGGKDDVANSP-----------------------FQSRNRFS 1134
    ***  ** *                                                *

Hum RTLPASAEERDRLLRRMESMRKEKRVYSRFEVFCKKEEASSPGAGEGPAEEGTRDSKVGK 1166
Cow RTLPAGAEERDRLLRRMESMRKEKRVYSRFEVFCKKEEPGALGAAEGPAEEDARDSKVGK 1168
Dog RTLPASAEDRDRLLRRMESMRKEKRVYSRFEVFCKKEEAGGPGAGEGPAEEDTRDSKVGK 1138
Rat RTLPASAEERDRLLRRMESMRKEKRVYSRFEVFCKKEDAGSSAAGDNLADEDTRDSKMGK 1196
Mos RTLPGSAEERDRLLRRMESMRKEKRVYSRFEVFCKKDEAGSSGAGDNLADEDTRDSKMGK 1196
Chk QRQPS--EDRDTLLKKMENMRKEKRVYSRFEVFCKKDE--HTSQSEEEYDTDAKDKKMGK 1120
Xep RPSPSSPEDRDILLKRMESIRKEKRVYSRFEVFCKKDE--QPSHAD-----DNDDKKAGK 1320
Zef -RAPVNPQERDNLLKRIESMRKEKKVYSRFEVLYRSREECCWERGS--------VEQANQ 1185
       *     ** **   *  **** *******

Hum FVPKILGTFKSKK 1179
Cow FMPKILGTFKSKK 1181
Dog FMPKILGTFKSKK 1151
Rat FVPKILGTFKSKK 1209
Mos FVPKILGTFKSKK 1209
Chk FMPKILGTFKTKK 1133
Xep IIPKLLGNLIKK- 1332
Zef FLIKSIE------ 1192
       *
```

Fig. S35. Protein sequence alignment of FAM83H orthologs from 8 vertebrates. The aligned protein sequences are from human (Hum) (*Homo sapiens*; gi|157311635, ref|NP_940890.3), cow (*Bos taurus*; gi|119906195, ref|XP_603315.3), dog (*Canis lupus familiaris*; gi|374091972, gb|AEY83660.1), rat (*Rattus norvegicus*; gi|194474062, ref|NP_001124037.1), mouse (Mos) (*Mus musculus*; gi|269914118, ref|NP_598848.2), chicken (Chk) (*Gallus gallus*; gi|118087467, ref|XP_423955.2), frog (Xep) (*Xenopus tropicalis*; gi|301629161, ref|XP_002943716.1), and zebrafish (Zef) (*Danio rerio*; gi|113682418, ref|NP_001038555.1). The number at the end of each line corresponds to the number of the amino acid with respect to the N-terminal Methionine. Asterisks (*) mark identical amino acids. The four F-X-X-X-F sequence motifs in human FAM83H that potentially mediate CK1-interactions (13) are highlighted in yellow: (F^{247}-X-X-X-F^{251}; F^{270}-X-X-X-F^{274}; F^{274}-X-X-X-F^{278}; F^{350}-X-X-X-F^{354}). Sites mutated in humans associated with disease-causing truncation mutations (nonsense or frameshift) are in bold and highlighted in cyan. Segments near the FAM83H C-terminus that show high sequence conservation during vertebrate evolution that may be important for FAM83H function are highlighted in green.

A

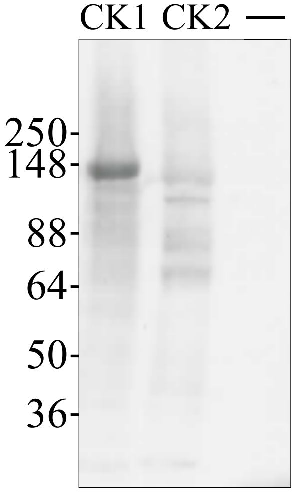

B

MARR**S**QSSSQGDNPLAPGYLPPHYKEYYRLAVDAL**T**EGGPEAYNRFLASEGAPDF
LCPEELEHVSRHLQPPQYVAREPPEGTPSDVDMDGSSGTYWPVNSDQAVPELDLG
WPLTFGFQGTEVTTLVQPPPPDSPSIKDEARRMIRSAQQVVAVVMDMFTDVDLLS
EVLEAAARRVPVYILLDEMNAQHFLDMADKCRVNLHHVDFLRVRTVAGPTYYCRT
GKSFKGHLKEKFLLVDCAVVMSGSYSFMWSFEKIHRSLAHVFQGELVSSFDEEFR
ILFAQSEPLVPSAGALARMDAYALAPYSGAGPLVGVPGVGAPTPFSFPKRAHLLF
PPPREEGLGFPSFLDPDRHFLSAFRREELQRMPGGALEPHTGLRPLARPTEAGPF
GELAGPRGFFQSRHLEMDAFKRHSYATPDGAGAVENFAAARQVSRQTFLSHGDDF
RFQTSHFQRDQLYQQHYQWDPQFAPARPQGLFEKLRAGRPGFADPDDFALGAGHR
FPELGADVHQRLEYVPSSASREVRHG**S**DPAFGPSPRGLEPSGASRPNLGQRFPCQ
ATLRQGLDTA**S**EAEPERRGGPEGRAGLRHWRLASYLSGCHGDGGEEGLPMEAEAC
EDEVLAPGGRDLLP**S**AFRTPAAFPAKGPKPGSGSGGGDS**S**EREGPEETSLAKQDS
FRSRLNPLIQRSSRLRSSLIFASQAEGAVGTAAATTEKVQLMHKEQTVSETLGPS
GEAVRSSASAKVAELLEKYKGPARDPGGAGGAVTSSSHSKAVVSQAWREEVVAPG
GAGTERRSLESCLLDLRDSFAQQLHQEAERHPGAASLTAAQLLDTLGGTDRLPSR
FLSAQGRSLSPQGRDSPPPEGLGTHQLPYSEPKGNPTPAYPERKGSPTPAYPERK
GSPTPAYPERKGSPTPAYPERKGSP**T**QAYPERKGSPTSGFPNRRGSPTTGLMEQK
GSP**T**STYPDRRGSPVPPVPERRGSPVPPVPERRG**S**L**T**FAGE**S**SKTGPTEEV**SS**GP
MEVLRKGSLRLRQLLSPKNERRGEDEG**S**FPTPQENGQPESPRRPSL**S**RGDS**T**EAA
AEERG**S**RVRLASATANALYSSNLRDDTKAILEQI**S**AHGQKHRGVPAPGPAHSSPD
VGRPTTAGDLAPDMSDKDKCSAIFRSDSLGTQGRLSRTLPGSAEERDRLLRRMES
MRKEKRVYSRFEVFCKKDEAG**S**SGAGDNLADEDTRDSKMGKFVPKILGTFKSKK

Fig. S36. FAM83H phosphorylation by CK1 *in vitro*. ***A:*** Autoradiograph of ^{33}P kinase assay. Purified mouse recombinant FAM83H was incubated with CK1, CK2, or no enzyme (–) as well as with radioactive ^{33}P-ATP. The reaction samples were separated by SDS-PAGE, and the dried gel was exposed to film. The CK1 reaction shows strong radioactivity at a specific band of ~130-kDa, demonstrating that CK1 can phosphorylate FAM83H *in vitro*. ***B:*** CK1 phosphorylation sites on FAM83H. The phosphorylation sites were determined by mass spectrometry of CK1-treated mouse FAM83H protein. The CK1-phosphorylated Serines and Threonines, which mainly locate at C-terminus of FAM83H, are marked bold.

Gene	Primer Name and Sequence	Amplicon Size
FAM83H 5.1	Fam5.1F: 5’AGGAGCCCTTGTCCTGTAGA<br>Fam5.1R: 5’GAAGCTTCTCGAACAGGCCT	847 bp
FAM83H 5.2	Fam5.2F: 5’GGCACCTGGAGATGGACG<br>Fam5.2R: 5’AGCCAGCACGTCGTCTTC	647 bp
FAM83H 5.3	Fam5.3F: 5’TTTGGCCTCCTACTTGAGCG<br>Fam5.3R: 5’AAAGGAGTCGCGCAGGTC	646 bp
FAM83H 5.4	Fam5.4F: 5’CGGAGCTGCTGGAGAAGTAC<br>Fam5.4R: 5’GATGGTAAGGGTGAGGCTGC	620 bp
FAM83H 5.5	Fam5.5F: 5’CTTCCCGCTTCCTCTCTGC<br>Fam5.5R: 5’TCGGACATATCTGGGGCCA	763 bp
FAM83H 5.6	Fam5.6F: 5’ACGCCTTGTACAGCAGCA<br>Fam5.6R: 5’CACTCAGCCAAGCCCCAA	593 bp

Fig. S37. *FAM83H* Exon 5 PCR primers and reaction conditions. 5 min at 94 ˚C followed by 35 cycles of denaturing at 94 ˚C for 30 s, annealing at 58 ˚C for 30 s and extension at 73 ˚C for 3 m, and a final extension at 72 ˚C for 5 m.
